# Impacts of platinum-based chemotherapy on subsequent testicular function and fertility in boys with cancer

**DOI:** 10.1093/humupd/dmaa041

**Published:** 2020-09-16

**Authors:** Lim Tian En, Mark F H Brougham, William Hamish B Wallace, Rod T Mitchell

**Affiliations:** MRC Centre for Reproductive Health, The Queen’s Medical Research Institute, The University of Edinburgh, Edinburgh, UK; Department of Paediatric Oncology, Royal Hospital for Sick Children, Edinburgh, UK; Department of Paediatric Oncology, Royal Hospital for Sick Children, Edinburgh, UK; MRC Centre for Reproductive Health, The Queen’s Medical Research Institute, The University of Edinburgh, Edinburgh, UK; Department of Paediatric Endocrinology, Royal Hospital for Sick Children, Edinburgh, UK

**Keywords:** cancer treatment, chemotherapy, human, prepubertal, child, testis, fertility, cisplatin, carboplatin, platinum

## Abstract

**BACKGROUND:**

Children with cancer often face infertility as a long-term complication of their treatment. For boys, compromised testicular function is common after chemotherapy and currently there are no well-established options to prevent this damage. Platinum-based agents are used to treat a wide variety of childhood cancers. However, platinum agents are not currently included in the cyclophosphamide equivalent dose (CED), which is used clinically to assess the risks to fertility posed by combination chemotherapy in children with cancer.

**OBJECTIVE AND RATIONALE:**

This was a systematic search of the literature designed to determine the evidence for effects of platinum-based cancer treatment on the prepubertal human testis in relation to subsequent testicular function and fertility.

**SEARCH METHODS:**

PubMed and EMBASE were searched for articles published in English between 01 January 1966 and 05 April 2020 using search terms including ‘cancer treatment’, ‘chemotherapy’, ‘human’, ‘prepubertal’, ‘testis’, ‘germ cells’, ‘testosterone’ and related terms. Abstracts were screened and full-text articles were obtained for those that met the three major inclusion criteria (age ≤12 years at treatment, exposure to platinum-based chemotherapeutic and measure of reproductive function). Screening of bibliographies for full-text articles was used to identify additional studies.

**OUTCOMES:**

Our initial search identified 1449 articles of which 20 (1.3%) studies (n = 13 759 males) met all inclusion criteria. A control group (healthy individuals or siblings) was included for 5/20 (25%) studies. A total of 10/20 (50%) studies provided sub-analysis of the relative gonadotoxicity of platinum-based agents.

The primary outcome measures were: pregnancies and fatherhood; semen analysis; and hormonal function. For pregnancies and fatherhood, three studies (n = 10 453 males) reported negative associations with platinum-agents, including the largest (n = 5640) controlled study (hazard ratio = 0.56, *P* = 0.0023), whilst two other studies (n = 1781) with platinum sub-analysis reported no association. For semen analysis (based on World Health Organization criteria), platinum-based chemotherapy was associated with azoospermia in one study (n = 129), whilst another (n = 44) found no association and the remainder did not perform platinum-based sub-analysis. For hormone analysis, conflicting results were obtained regarding potential associations between platinum-based agents and elevated FSH (a proxy for impaired spermatogenesis); however, the majority of these studies were based on low numbers of patients receiving platinum-based chemotherapy.

**WIDER IMPLICATIONS:**

Overall, these results indicate that platinum-based chemotherapy should be included in clinical calculators, for example CED, used to determine gonadotoxicity for childhood cancer treatment. These findings have important implications for clinicians regarding counselling patients and their carer(s) on fertility risk, guiding requirements for fertility preservation strategies (e.g. testicular tissue cryopreservation) and modification of treatments to reduce or eliminate the risk of infertility in childhood cancer survivors.

## Introduction

Childhood cancer rates have increased dramatically over recent decades and it is estimated that one in 530 young adults is a survivor of childhood cancer (Ward *et al.*, 2014). The increasing incidence, coupled with remarkable improvements in cure rates (>80% 5-year survival), have resulted in an increase in young adults experiencing treatment-related morbidity (Anderson *et al.*, 2015). Infertility is a well-recognized complication of cancer treatment, although the relative contribution of individual agents to fertility risk in childhood cancer survivors has not been fully elucidated. Amongst chemotherapeutics, alkylating agents (e.g. cyclophosphamide) are known to affect fertility in males and an objective measure of relative gonadotoxicity between chemotherapeutics, the cyclophosphamide equivalent dose (CED), has been established (Green *et al.*, 2014b). This calculation can be used to estimate cumulative gonadotoxicity of multiple agents for a specific regimen.

Platinum-based chemotherapy (cisplatin or carboplatin) agents are used in treatment of a variety of childhood cancers including brain tumours, osteosarcoma, neuroblastoma, hepatoblastoma and germ cell tumours (GCTs). Whilst most of the commonly used chemotherapy drugs are included in the CED calculator, platinum-based chemotherapy is a notable exception. Therefore, the present study aimed to review the literature relating to effects of exposure to platinum-based chemotherapy during childhood on gonadal function and future fertility.

## Methods

We performed a systematic search of the existing literature reporting the effects of platinum-based chemotherapy for childhood cancer on testicular development and function. We followed PRISMA guidelines for reporting systematic reviews (Moher *et al.*, 2009). Given that the search was conducted to identify clinical and laboratory experimental data, the study was not registered with PROSPERO.

### Information source

PubMed and EMBASE were searched (to 05 April 2020) to identify original clinical and experimental studies describing the testicular effects of platinum-based chemotherapy on prepubertal human testis. Additional studies were identified from a bibliographic screen of reference lists of included articles.

### Inclusion criteria

The inclusion criteria for study selection were:

- articles published between 01 January 1966 and 05 April 2020 and written in English;
- clinical and experimental studies; and
- outcomes relating to reproductive function including fatherhood, sperm counts, reproductive hormones (LH, FSH, testosterone, inhibin B and anti-Müllerian hormone, testicular volume and puberty).

### Exclusion criteria

The exclusion criteria for the review were:
  - exposure to cancer treatment during (peri)puberty (≥12 years) and adulthood;
  - studies that did not include human data or experimental studies involving human tissue/cells; and
  - review publications.

### Search and study selection

**Figure 1.**
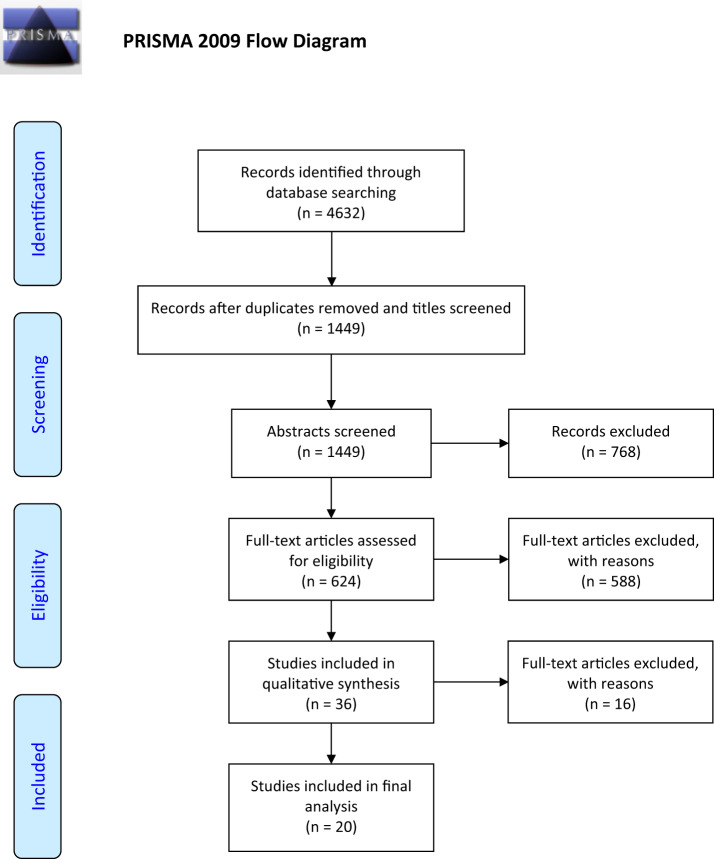
**PRISMA flow diagram for the selection of studies to assess the impacts of platinum-based chemotherapy on subsequent testicular function and fertility in boys with cancer.** PRISMA, preferred reporting items for systematic reviews and meta-analysis.

The search terms and strategy (Supplementary Table SI) used for the systematic review were adapted from a previous study (Skinner *et al.*, 2017). After removing duplicates and screening by titles, we identified 1449 studies. R.T.M. and L.T.E. screened abstracts independently to determine eligibility. For abstracts that were not selected by both authors, these were discussed, and a joint decision made regarding inclusion for full-text screening. Full texts were obtained for 624 studies that met the criteria for inclusion. An initial screen of full texts identified 588 studies that were excluded for not meeting at least one aspect of the inclusion criteria (Supplementary Table SII). Of the 36 remaining papers that were subjected to a detailed assessment, a further 16 papers were excluded for a variety of reasons (Supplementary Table SIII). In total, 20 papers were included in the final analysis. A PRISMA flowchart for the search and study selection is presented in Fig. 1.

### Summary measures

Data were extracted in relation to four categories: pregnancy and fertility; semen analysis; hormones and testicular volume; and experimental studies (tissue and cells). Risk of bias within and between studies was assessed using the principles described for the assessment of risk of bias in non-randomized studies (Sterne *et al.*, 2019). This included an assessment of risk of confounding and bias in selection, information and reporting for each study (Supplementary Table SIV). For each category, risk of bias was assessed as ‘low’, ‘moderate’, ‘serious’, ‘critical’ or ‘no information’. Given the heterogeneity of reported summary measures, all methods of reporting were included.

## Results

### Studies assessing testicular function and fertility after treatment with platinum agents

Overall, 20 studies reported testicular function and fertility following exposure to platinum-based agents (Table I). The risk of bias for each study is presented in Supplementary Table SIV. Overall risk of bias was lower in the studies that included a sub-analysis of platinum exposure, compared with cohort studies that did not include a sub-analysis. A detailed description of the data extracted from these studies is provided in Supplementary Table SV.

**Table I:** Summary of 20 studies including use of platinum agents, prepubertal study participants and an outcome measure relating to fertility.

Study	Study design	Level^*^	Control group	Fertility outcome
Flamant *et al.* (1984)	Multi-centre cohort	3	No	Pregnancy and fertility
Wallace *et al.* (1989)	Single-centre cohort	3	No	Gonadotrophins
Kiltie and Gattamaneni (1995)	Single-centre cohort	3	No	Gonadotrophins
Muller *et al.* (1996)	Single-centre cohort	3	Yes^a^	Semen analysis; testicular volume
Hale *et al.* (1999)	Single-centre cohort	3	No	Gonadotrophins
Relander *et al.* (2000)	Single-centre cohort	3	No	Semen analysis; gonadotrophins; testicular volume
Longhi *et al.* (2003)	Single-centre cohort	3	No	Semen analysis; gonadotrophins
Ridola *et al.* (2009)	Multi-centre cohort	2	No	Pregnancy and fertility; gonadotrophins
Romerius *et al.* (2011)	Single-centre cohort	3	No	Semen analysis
Tromp *et al.* (2011)	Single-centre cohort	3	No	Pregnancy and fertility; gonadotrophins
Odagiri *et al.* (2012)	Single-centre cohort	3	No	Gonadotrophins
Reinmuth *et al.* (2013)	Multi-centre cohort	3	No	Pregnancy and fertility
Green *et al.* (2014a)	Multi-centre cohort	3	No	Semen analysis
Green *et al.* (2014b)	Multi-centre cohort	3	No	Pregnancy and fertility
Wasilewski-Masker *et al.* (2014)	Multi-centre cohort	3	Yes	Pregnancy and fertility
Brignardello *et al.* (2016)	Single-centre cohort	3	No	Gonadotrophins
Chemaitilly *et al.* (2016)	Case report	5	No	Gonadotrophins; testicular volume
Chow *et al.* (2016)	Multi-centre cohort	3	Yes^b^	Pregnancy and fertility
Isaksson *et al.* (2018)	Multi-centre cohort	2	Yes^a^	Gonadotrophins
Utriainen *et al.* (2019)	Multi-centre cohort	2	Yes^a^	Pregnancy and fertility; gonadotrophins

* Level of Evidence (adapted from https://www.cebm.net/2016/05/ocebm-levels-of-evidence/).

1—Properly powered and conducted randomized clinical trial; systematic review with meta-analysis.

2—Well-designed controlled study without randomization; prospective comparative cohort study.

3—Case–control studies; retrospective cohort study.

4—Case series with or without intervention; cross-sectional study.

5—Opinion of respected authorities; case reports.

a Healthy age-matched controls.

b Healthy siblings.

### Fertility and fatherhood

We identified eight papers which included pregnancy and fatherhood as outcome measures of fertility (Table II). Several papers reported data from a multi-centre cohort Childhood Cancer Survivor study (CCSS) (Robison *et al.*, 2009). One questionnaire-based study including 1622 male cancer survivors aged 0–21 years at diagnosis reported that cisplatin exposure was not significantly (*P* > 0.20) associated with infertility, defined as difficulty in getting a female partner pregnant (>1 year) (Wasilewski-Masker *et al.*, 2014).

**Table II:** Summary of studies involving pregnancy and fertility outcome measures and reported to include platinum-including treatment regimens.

Study	Diagnoses	Males (n)	Age at diagnosis (years)	Chemotherapy	Platinum analysis	Summary finding
Flamant *et al.* (1984)	Nonseminomatous GCT	13	1–14	Actinomycin D, Cyclophosphamide, Vincristine, Doxorubicin, Bleomycin, Cisplatin	No	One case of sterility attributed to whole abdominal irradiation.
Ridola *et al.* (2009)	Various	159	9–19	Ifosfamide versus Cyclophosphamide protocols	Yes	Platinum agents reported to have no impact on testicular function but data not shown.
Tromp *et al.* (2011)	Various	565	0–17.8	Various	Yes	73 partner pregnancies (56 natural conception). Platinum sub-analysis for hormones only.
Reinmuth *et al.* (2013)	Various	234	0–15	Cyclophosphamide, Ifosfamide, Cis/Carboplatin, Etoposide	Yes^c^	Higher rate of infertility for platinum-based agents (carboplatin or cisplatin).
Green *et al.* (2014b)	Various	4579	5–20	Various	Yes	Lower rate of fatherhood after cisplatin compared to no cisplatin. Statistical data not shown.
Wasilewski-Masker *et al.* (2014)	Various	1622	9^a^	Various	Yes	Platinum agents did not have independent association with fertility—preliminary modelling.
Chow *et al.* (2016)	Various	5640	≤20	Various	Yes^c^	Cisplatin associated with reduced pregnancy (≥488 mg/m^2^) and livebirth (≥355 mg/m^2^) rates.
Utriainen *et al.* (2019)	Neuroblastoma	9	0.2–3.6^b^	Various, HSCT	Yes	^d^Cisplatin cumulative dose did not correlate with testicular size.

Note that summary findings relate to patients receiving treatment when <12 years of age.

GCT, germ cell tumour; HSCT, haematopoietic stem cell transplant.

a Mean age.

b Interquartile range.

c Multivariate and univariate regression analysis.

d One patient fathered a child (patient received cisplatin).

Other studies from the CCSS have observed an impact on fertility with exposure to cisplatin. A study of 5640 male patients, of whom 455 were treated with cisplatin, reported that higher doses were significantly associated with a lower likelihood of siring pregnancies and livebirths (Chow *et al.*, 2016). Multivariable models found that the upper tertile of cisplatin dose (≥488 mg/m^2^) was associated with a reduced likelihood of siring pregnancies (hazard ratio (HR) = 0.56, *P* = 0.0023), while lower doses of cisplatin (355–487 mg/m^2^) were associated with a reduced likelihood of siring livebirths (HR = 0.64, *P* = 0.071). Another study reported a lower percentage of fatherhood among patients who received cisplatin (10.9%) compared to patients who did not (14.9%), although no statistical data was provided (Green *et al.*, 2014b).

Other studies have drawn conflicting conclusions on the impact of platinum agents on fertility. A study of 159 males ranging from 0 to 21 years at diagnosis using fatherhood and hormone measurements as outcome measures reported that addition of platinum agents to cyclophosphamide or ifosfamide did not increase the risk of gonadal dysfunction (statistical data not shown) (Ridola *et al.*, 2009).

A study of 234 males aged 0–15 years reported an increased likelihood of infertility (unsuccessful at fathering a child or abnormal fertility test) among patients receiving cisplatin or carboplatin (Reinmuth *et al.*, 2013). However, this increased risk was not statistically significant under both univariate and multivariate analysis, even at high doses (≥500 mg/m^2^ for cisplatin, ≥2000 mg/m^2^ for carboplatin). In a small study involving nine males with neuroblastoma ranging from 0.2 to 3.6 years of age at diagnosis, only one male patient was observed to have fathered a child (Utriainen *et al.*, 2019). This patient had received cisplatin and etoposide as induction chemotherapy, followed by high-dose melphalan. However, participants had also received total body irradiation (TBI) and haematopoietic stem cell transplants (HSCTs), which are known to be sterilizing treatments (Freycon *et al.*, 2019). A study of a cisplatin-including regimen for GCT in children also reported a single case of sterility, which was attributed to whole abdominal irradiation (Flamant *et al.*, 1984). In a larger study of 565 males, aged 0–17.8 years at diagnosis, 73 of the participants sired pregnancies and 56 of these men conceived naturally (Tromp *et al.*, 2011). A total of 120 conceptions were reported with 103 livebirths and 14 miscarriages. However, the study did not include an individual analysis of platinum agents with regards to pregnancy and fertility.

### Semen analysis

Semen analysis represents a reliable indicator of fertility status in adult men. We identified five studies reporting the impact of platinum-based chemotherapy regimens for childhood cancer treatment on sperm count in adulthood (Table III). Methods and cut-offs for semen analysis varied between studies (Supplementary Table SV) and 3/5 studies reported the use of World Health Organization standards relevant to the period of study (Table III). All studies combined semen analysis with assessment of gonadotrophins, testicular volume or pubertal staging. A study involving 129 males diagnosed between the ages of 0–17 years reported azoospermia in 4/5 (80%) of those who had received what the authors considered a ‘sterilizing’ dose (>500 mg/m^2^) of cisplatin (without radiotherapy), compared with 0/8 (0%) of those who had received <500 mg/m^2^ of cisplatin (Romerius *et al.*, 2011). Two studies included a control group of age-matched healthy participants (Muller *et al.*, 1996; Utriainen *et al.*, 2019). A study involving 33 males aged 0–19 years with various cancers showed azoospermia in 2/14 (14%) and oligozoospermia in 3/14 (21%) of long-term survivors compared with normospermia in 8/8 (100%) of healthy age-matched controls (Muller *et al.*, 1996). Patients with azoospermia were significantly more likely to have been exposed to alkylating agents than those with normospermia. However, the contribution of cisplatin to the semen parameters was not reported (Muller *et al.*, 1996). The second study involving a control group investigated nine male childhood cancer survivors who commenced treatment with alkylating-based chemotherapy, with or without cisplatin, between the ages of 0–3 years (Utriainen *et al.*, 2019). Only one patient had a sperm count performed in adulthood. He had received cisplatin and had fathered a child, although interestingly he had a low total sperm count (0.8 × 10^6^/ml) with oligo-asthenozoo-spermia (Utriainen *et al.*, 2019). Specific correlation of semen analysis with platinum exposure was only reported in one study (Green *et al.*, 2014a): this study involved 214 subjects treated for various cancers from the age of 0–19 years. Sub-analysis of those receiving cisplatin for neuroblastoma or osteosarcoma did not reveal a statistically significant effect of cisplatin on the risk of azoospermia (Green *et al.*, 2014a). However, there were only 44 patients in this subgroup and these individuals had also received alkylating agents as part of their regimen, which may have affected the potential to detect a significant effect (Green *et al.*, 2014a). A study involving 96 males, of whom 11 were prepubertal at diagnosis, reported one case of a prepubertal boy (>12 years old) in which semen analysis was performed in adulthood (Longhi *et al.*, 2003). This individual was azoospermic and had received cisplatin treatment, in addition to ifosfamide, as part of his regimen. None of the patients who were ≤12 years old at diagnosis had semen analysis performed (Longhi *et al.*, 2003).

**Table III:** Summary of studies involving semen analysis in childhood cancer survivors exposed to treatment regimens that are reported to include platinum-based chemotherapy.

Study	Diagnoses	Age at diagnosis (years)	Males (n)	Chemotherapy treatment	Platinum analysis	Summary finding
Muller *et al.* (1996)	Various	7–17	33	Various	No	Differences between azoospermic and normospermic survivors were not detected for non-alkylating agents.
Longhi *et al.* (2003) ^a^	Osteosarcoma	10–42	96	Methotrexate, Cisplatin, Doxorubicin ± Ifosfamide ± Etoposide	No	None of the patients <12-years-old at diagnosis had a sperm count performed.
Romerius *et al*. (2011 ^a^	Various	0–17	129	Alkylating agents/Cisplatin	No	Azoospermia in 4/5 (80%) versus 0/8 (0%) based on a threshold dose of >500 mg/m^2^ cisplatin.
Green *et al.* (2014a) ^a^	Various	0–19	214	Various	Yes^b^	No increase in azoospermia in subgroup receiving cisplatin treatment for neuroblastoma/osteosarcoma.
Utriainen *et al.* (2019)	Neuroblastoma	0–3	9	Various	Yes	Sperm count performed in one patient who had received cisplatin—oligozoospermia although reported to have fathered a child.

Note that summary findings relate to patients receiving treatment when <12 years of age.

a Semen analysis reported to be based on World Health Organization criteria.

b Sub-group analysis for cisplatin in osteosarcoma and neuroblastoma survivors.

### Hormone analysis and puberty

Gonadotrophin measurements can be used to identify hypogonadism in adulthood. We identified 13 studies of gonadotrophins, testicular volume and puberty in childhood cancer survivors exposed to treatment regimens that are reported to include platinum-based chemotherapy (Table IV). A study including 33 pubertal males (aged 7–17 years at diagnosis) with a range of malignancies demonstrated a significant increase in basal FSH and LH when compared with healthy age-matched controls, indicating impaired spermatogenesis in the cancer survivor group. Of these, 29 males had received chemotherapy, which included alkylating agents for 24 patients (Muller *et al.*, 1996). FSH levels correlated with testicular volumes and sperm counts (see above), providing further evidence to indicate impaired spermatogenesis. Despite the higher LH, suggesting a degree of Leydig cell failure, no differences in testosterone were identified between cancer survivors and controls (Muller *et al.*, 1996). Whilst some patients had received cisplatin, the relationship between cumulative cisplatin dose and testicular function was not reported.

**Table IV:** Summary of studies involving gonadotrophins, testicular volume and puberty in childhood cancer survivors exposed to treatment regimens that are reported to include platinum-based chemotherapy.

Study	Diagnoses	Males (n)	Age at diagnosis (years)	Chemotherapy	Cranial irradiation	Platinum analysis	Summary finding
Wallace *et al.* (1989)	Various	8	7–14	Multiple regimens	No	No	^d^Gonadotrophins remained low at follow-up (≤3 years post-diagnosis) in keeping with retained prepubertal status.
Kiltie and Gattamaneni (1995)	IGCT	18	3–15	Multiple regimens	Yes (n = 18)	No	^d^Precocious puberty in two boys prior to treatment and normal puberty in another two. Specific impact of cisplatin not investigated.
Muller *et al.* (1996)	Various	33	7–17	Multiple regimens	Yes (n = 6)	No	Overall increase in basal FSH and LH compared with healthy controls. Specific impact of cisplatin not investigated.
Hale *et al.* (1999)	GCT	26	0–16	Multiple regimens	No	No	^d^Puberty normal in all patients. Specific impact of cisplatin not investigated.
Relander *et al.* (2000)	Various	77	0–17	Multiple regimens	Yes (n = 28)	No	Puberty and testosterone normal in 98% and 95%, respectively. Raised FSH in 14% of entire cohort. Only one patient received cisplatin.
Ridola *et al.* (2009)	Various	159	0–20	Cyclophosphamide versus Ifosfamide	No	Yes	Inclusion of platinum-based agents reported to have no effect on testicular function but data not shown.
Romerius *et al*. (2011)	Various	129	0–17	Multiple regimens	Yes (n = 61)^a^	No	Elevated FSH in 32.5% of the entire cohort. Specific impact of cisplatin not investigated.
Tromp *et al.* (2011)	Various	565	0–17	Multiple regimens	Yes (n = 135)	Yes^c^	Raised FSH in 33% and low testosterone in 12%. No association between cisplatin and FSH.
Odagiri *et al.* (2012)	IGCT	14	8–19	Carboplatin and Etoposide	Yes (n = 14)	No	No impairment of gonadotrophin secretion at 2–11 years post-diagnosis.
Brignardello *et al.* (2016)	Various	199	0–18	Various	Yes (n = 71)	Yes^d^	FSH or low Inhibin B in 35% (alkylators) versus 60% (alkylators + platinum agent). Compared with 12% (no alkylators or platinum agents).
Chemaitilly *et al.* (2016)	Glioma	1	2	Carboplatin, Vincristine, Temozolomide, Lomustine, Thioguanine, Procarbazine	No	No	^d^Case report aged 11 years. Tanner stage 4 with testicular volumes 4.5 ml indicating impaired spermatogenesis.
Isaksson *et al*. (2018)	Various	121	5–15	Multiple regimens	Yes (n = 12)	No	Raised FSH in 5.8% of childhood cancer survivors compared with 1% of healthy age-matched controls.
Utriainen *et al.* (2019)	Neuroblastoma	9	0–3	Multiple regimens	Yes (n = 4)	Yes	^d^Significantly raised FSH and reduced TV compared with healthy controls. No correlation between cumulative cisplatin dose and TV.

IGCT, intracranial germ cell tumour; TV, testicular volume.

a Includes all non-testicular irradiation.

b Multivariate and univariate regression analysis.

c Comparison of alkylators ± platinum agent.

d Summary includes only patients receiving treatment <12 years of age.

Assessment of the hypothalamic–pituitary–gonadal (HPG) axis was conducted on a cohort of 14 young males (aged 8–19 years) with intracranial GCT (IGCT), of whom six were <12 years at diagnosis (Odagiri *et al.*, 2012). All patients were treated with carboplatin and etoposide. However, patients also received cranial irradiation, which can impair pituitary gonadotrophin secretion. None of the six boys had impairment of gonadotrophin secretion by last assessment, which was 2–11 years after diagnosis (Odagiri *et al.*, 2012). Whether this related only to hypogonadotrophic hypogonadism is not clear as no definition of what constituted abnormal gonadotrophin production was given.

Another study of patients with IGCT, including 18 boys aged 3–15 years, reported pubertal timing in four boys treated before the age of 12 years (Kiltie and Gattamaneni, 1995). Of these, two had precocious puberty prior to therapy, whilst two entered puberty at a normal age (11–12 years). Some patients in this study received platinum-based treatment; however, specific treatment regimens for individual patients were not described.

A case report of an 11-year-old boy treated with alkylating agents and carboplatin for a glioma at the age of 2 years reported puberty with evidence of impaired spermatogenesis (Chemaitilly *et al.*, 2016). He was Tanner stage 4 with a testosterone of 8.9 nmol/l, although his testicular volumes were consistent with early puberty (4.5 ml). Subsequent follow-up into adulthood was not reported.

Normal puberty has been reported in 26 males (aged 0–18 years) treated for GCT (Hale *et al.*, 1999). Treatment regimens were varied in terms of exposure to radiotherapy and/or platinum-based chemotherapy and the relative contribution of cisplatin cannot be concluded (Hale *et al.*, 1999).

In a study involving 129 childhood cancer survivors (Romerius *et al.*, 2011), gonadotrophins correlated with risk of azoospermia. Elevated FSH levels (>10.9 IU/l) were identified in 42 (32.5%) of patients of whom 22 were azoospermic giving a positive predictive value of 50% and a negative predictive value of 99%. Similar associations were seen between combined testicular volume ≤24 ml and azoospermia (Romerius *et al.*, 2011). However, the study did not distinguish between impacts attributed to cisplatin and alkylating agents.

Gonadotrophins were evaluated in 159 boys (aged 4–20 years) treated for various malignancies (Ridola *et al.*, 2009). Patients were grouped according to whether they received ifosfamide or cyclophosphamide as their sole alkylating agent in the treatment regimen and 42 had also received a platinum-based agent (cisplatin or carboplatin). Only two patients had a low testosterone within this cohort. Higher doses of cyclophosphamide (>12 g/m^2^) were associated with an increased risk of elevated FSH compared with lower doses and with all doses of ifosfamide. The inclusion of platinum-based agents to regimens was reported to have no impact on testicular function, although the data to support this conclusion was not shown (Ridola *et al.*, 2009).

A study of 77 men who had been treated for a variety of malignancies included gonadotrophin and testosterone measurements in 66 (Relander *et al.*, 2000). Testosterone was in the normal adult range for 65/66 and normal puberty was reported for 62/66 men. FSH was normal in 42/66 (63%) and in nine patients FSH was raised, indicating impaired spermatogenesis. Of these nine patients, seven had a sperm count performed and this was abnormal (five azoospermia and two oligozoospermia) in all cases. However, few patients in this study had received cisplatin. One patient with Ewing Sarcoma received cisplatin and cyclophosphamide and he was found to have testicular volumes <15 ml, whilst two patients with testicular GCT had received cisplatin but these patients were aged >14 years at diagnosis (Relander *et al.*, 2000).

Long-term follow-up of gonadotrophin levels was conducted in a large cohort of 565 survivors of childhood cancer diagnosed between the ages 0–17 years and surviving for a minimum of 5 years (Tromp *et al.*, 2011). The majority (90%) had received chemotherapy. FSH levels were obtained in 488 (86%) patients and were raised (>10 IU/l) in 121 (33%) cases. Raised LH and low testosterone, indicating primary Leydig cell failure, were reported in 2.9% and 12.4% of cases, respectively. The impact of platinum agents on FSH level was assessed using linear regression analysis and no significant association was identified either by univariate or multivariate analysis with odds ratios of 0.68 (0.35–1.32) and 2.29 (0.89–5.89), respectively. However, the number of patients receiving platinum-based chemotherapy represented a very small proportion of the cohort.

Cumulative exposure to cisplatin has been reported in relation to gonadotrophin data in a cohort of 20 patients (nine males) diagnosed with high-risk neuroblastoma at between 0 and 3 years of age (Utriainen *et al.*, 2019). Cisplatin was part of their initial treatment and carboplatin was used for conditioning for HSCT, which included TBI in 10 (50%) cases. After a median follow-up time of 15 years, male survivors (n = 6) had significantly higher FSH (26.3 vs 3.6 IU, respectively; *P* < 0.001) and lower cumulative testicular volumes (8.5 vs 39 ml, respectively; *P* < 0.001) compared with healthy controls. Despite the high FSH, 3/5 patients in the non-TBI group had testicular volumes >15 ml, whereas all (4/4) patients receiving TBI had testicular volumes <10 ml. No correlation between cumulative cisplatin dose and testicular volume was identified, which may reflect the fact that these individuals also had HSCT and in many cases TBI. Of the nine patients, eight entered puberty spontaneously (three early), whilst one patient in the TBI group required pubertal induction. All four patients in the TBI group required testosterone replacement, whilst 0/5 in the no-TBI group required testosterone.

A study of 199 childhood cancer survivors aged <18 years at diagnosis (minimum follow-up of 5 years) had their HPG axis assessed (Brignardello *et al.*, 2016). Impaired spermatogenesis, defined as FSH >10 IU/l or inhibin B <100 pg/ml, was identified in 52/130 (35%) patients treated with alkylating agents, compared to 14/23 (60%) patients treated with a combination of alkylating and platinum-based chemotherapy. For those treated with chemotherapy other than alkylating or platinum-based agents, impaired spermatogenesis only occurred in 2/17 (11.8%). Biochemical evidence for impaired spermatogenesis was confirmed by semen analysis in all patients (41/68) who provided a sample; however, the proportion that had received platinum-based chemotherapy was not described. In addition to impairment of spermatogenesis, Leydig cell failure (defined as raised LH and total testosterone <3 ng/dl) occurred in 13/147 (8.8%) of patients receiving alkylating chemotherapy, compared with 2/23 (8.7%) of those receiving alkylating agents in combination with platinum-based chemotherapy (Brignardello *et al.*, 2016).

In a study of 14 male patients treated for CNS GCT, four patients were aged <12 years at diagnosis (Buckner *et al.*, 1999). All four patients received chemotherapy, including etoposide and cisplatin, and localized radiotherapy. The study reported no post-treatment effects on growth and development; however, hormone levels were not reported.

Whilst not specifically reporting effects of cisplatin treatment, impaired spermatogenesis has also been shown in a cohort of 121 male childhood cancer survivors (Isaksson *et al.*, 2018). Patients were diagnosed before the age of 18 years and were a minimum of 3 years post-treatment. Primary hypogonadism (definition includes FSH >10 IU/l) was reported in 7/121 (5.8%), compared to 1/122 (0.8%) of the control patients.

A study by Wallace *et al.* (1989) included eight male patients aged 7–12 years at diagnosis, who had received cisplatin treatment. Three were described as prepubertal at diagnosis. In the prepubertal patients, serum levels of gonadotrophins were low at follow-up, in keeping with their prepubertal status. However, no further follow-up data were available for these patients.

### Experimental evidence for effects on testicular development and function

No laboratory studies were identified that investigated the effect of platinum-based chemotherapy on human testicular tissues or cells.

## Discussion

The data on the impact of platinum-based chemotherapy in males on partner pregnancy rates provide conflicting results. Whilst one large study reported no effect of platinum-based chemotherapy on pregnancy rates (Wasilewski-Masker *et al.*, 2014), the CCSS demonstrated a significant reduction in pregnancy rates based on a dose threshold of ∼500 mg/m^2^ (Green *et al.*, 2014b) and another study involving the CCSS cohort reported similar findings with a lower threshold of ∼350 mg/m^2^ (Chow *et al.*, 2016). A major limitation of the studies is the use of self-reporting of fertility using questionnaires. These may be prone to selection bias and variations in interpretation of the terms used to define infertility. In addition, it does not account for co-existing morbidities that may impact fertility e.g. obesity, or individual wish to father a child.

Semen analysis can provide an accurate objective measure of fertility potential in males and may be less prone to bias. However, obtaining a semen sample is challenging from a practical perspective and such studies are prone to participation bias. The majority of the studies that include sperm counts are limited by low numbers of patients providing a sample or by lack of sub-analysis to determine the contribution of platinum-based chemotherapy to azoospermia. The only study to specifically assess this did not find an association, although this may be confounded by the concomitant use of alkylating agents and low numbers of patients receiving platinum-based treatments (Green *et al.*, 2014a).

Whilst the study of Isaksson *et al.* (2018) did not specifically report on cisplatin exposure in the childhood cancer survivor cohort, it did report a cohort of adult testicular cancer patients in whom there was a significantly increased risk of hypogonadism in those who received >4 cycles of cisplatin-based chemotherapy. This is in keeping with the studies reporting cisplatin-induced fertility impacts on men with testicular GCT (Lampe *et al.*, 1997; Brydoy *et al.*, 2010). The finding of persistent azoospermia in men after platinum-based chemotherapy suggests damage to the spermatogonial stem cell (SSC) population, which is also the likely target for damage by platinum-based chemotherapy during childhood, leading to impairment of fertility in adulthood. A reduced number of spermatogonia, including SSCs, has been reported in several recent studies involving histological examination of testicular biopsies in boys with cancer (Poganitsch-Korhonen *et al.*, 2017; Stukenborg *et al.*, 2018; Valli-Pulaski *et al.*, 2019; Portela *et al.*, 2020). However, these studies did not include data on the contribution of cisplatin to the germ cell loss.

Gonadotrophins provide an indirect assessment of testicular function and spermatogenesis. Raised gonadotrophins in the context of chemotherapy or radiotherapy indicate primary gonadal failure and an FSH threshold can be applied to identify azoospermia in adults with good sensitivity and specificity (Kelsey *et al.*, 2017). However, the use of gonadotrophins in determining impairment to spermatogenesis and fertility in childhood cancer survivors is limited because these need to be measured post-puberty due to the quiescence of the HPG axis during prepuberty. The specific contribution of cisplatin was only assessed in 2/12 studies reporting gonadotrophin data, with conflicting results. One study showing no association was limited by the low numbers receiving cisplatin (Tromp *et al.*, 2011). The other study did report an increased frequency of raised FSH in those receiving a combination of alkylators and cisplatin, compared to alkylators alone (Brignardello *et al.*, 2016); however, the specific regimens and cumulative doses are not reported.

The major limitations of clinical studies involving childhood cancer survivors relate to the number of patients receiving specific regimens and the challenges of identifying the relative contribution of an individual agent to infertility in adulthood. Experimental studies can contribute to the understanding of the impact of platinum-based chemotherapy by exposing human testis tissues to single agent chemotherapy in human-relevant model systems. Such systems have recently been developed utilizing *in vitro* and xenograft approaches and can be applied to the study of exposure to pharmacological agents on germ and somatic cells of the testis (van den Driesche *et al.*, 2015; Jorgensen *et al.*, 2018). The *in vitro* approach has recently been used to expose prepubertal rodent testis tissue to a variety of chemotherapy drugs, including cisplatin (Smart *et al.*, 2018; Allen *et al.*, 2020). These studies report significant reductions in total germ cell and putative SSCs following exposure to concentrations of platinum-based chemotherapy that reflect serum levels in patients treated with these agents. To date, such experimental studies have not been replicated using human prepubertal testcicular tissues from patients who are having tissue stored for fertility preservation. In addition to conducting such studies, retrospective examination of archived testicular tissues that have already been exposed to platinum-based chemotherapy may be performed, similar to previous studies investigating the effects of exposure to alkylating agents (Poganitsch-Korhonen *et al.*, 2017).

Overall, the results of this study indicate that platinum-based chemotherapy should be taken into consideration by clinicians when counselling patients and their carer(s) on the fertility risk associated with cancer treatment. Platinum-exposure should also be factored into discussions regarding fertility preservation options (e.g. testicular tissue cryopreservation) in prepubertal boys. However, it should be emphasized that identifying clear thresholds for testicular dysfunction following exposure to platinum-based chemotherapy in childhood requires further study. Children receiving treatment for osteosarcoma, hepatoblastoma and extracranial GCT may represent appropriate groups to determine the individual contribution of platinum agents, as these patients will often receive platinum-based chemotherapy without concomitant exposure to alkylating agents. These studies should include detailed clinical histories and drug exposures (including cumulative doses) for all patients. Regular follow-up of reproductive function, including pubertal assessment, hormone measurements and semen analysis, should also be performed.

## Conclusion

Whilst many studies report the reproductive outcomes of childhood cancer survivors who have received platinum-based chemotherapy, relatively few have investigated the individual contribution of these agents to gonadal function, especially fertility. In addition, human-relevant experimental model systems in which to test the impact of specific chemotherapeutics on testicular development and function are lacking. Whilst this study indicates that platinum-based agents should be included in fertility risk assessments in prepubertal boys, it also highlights the importance of conducting large-scale prospective follow-up studies in these patients.

## Supplementary Material

dmaa041_Supplementary_Data
