# Supplementary material for: Impacts of platinum-based chemotherapy on subsequent testicular function and fertility in boys with cancer
Source: Hum Reprod Update. 2020 Sep 16;26(6):874–85. doi: 10.1093/humupd/dmaa041 (PMC7600277; doi:10.1093/humupd/dmaa041)
Supplement: dmaa041_Supplementary_Data [file dmaa041_supplementary_data.zip › dmaa041-suppl_data/Supplementary_Table_SI final.docx]

**Supplementary Table SI** Search terms and strategy for identification of publications relating to fertility outcomes after platinum-based chemotherapy in childhood cancer survivors. Search strategy adapted from Skinner et al, (2017).

**Cisplatin and Germ Cell**

(((((male[tiab] OR males OR boy OR boys OR boyfriend OR boyhood)) AND ((((((((((((((((((((((alkylating) OR antineoplastic alkylating agents) OR alkylating agents) OR alkylating agent*) OR Carboplatin) OR NSC 241240) OR Carboplat) OR carboplatin*) OR Platinum) OR 41575-94-4) OR Cisplatin) OR Platinum Diamminodichloride) OR Platinol) OR Platidiam) OR Platino) OR NSC-119875) OR Biocisplatinum) OR Cddp) OR Capc) OR cisplatin*) OR cis-DDP 3) OR 15663-27-1)) AND (((((((((((((((((((((((((((((((((((((((((((((((((((((((((((((((((((((((leukemia) OR leukemic*) OR leukaemi*) OR childhood ALL) OR AML) OR lymphoma) OR lymphom*) OR Hodgkin) OR hodgkin*) OR T-cell) OR B-cell) OR non-hodgkin) OR sarcoma) OR sarcom*) OR sarcoma) OR Ewing's) OR Ewing*) OR osteosarcoma) OR osteosarcom*) OR wilms tumor) OR wilms*) OR nephroblastom*) OR neuroblastoma) OR neuroblastom*) OR rhabdomyosarcoma) OR rhabdomyosarcoma*) OR teratoma) OR teratom*) OR hepatoma) OR hepatom*) OR hepatoblastoma) OR hepatoblastom*) OR PNET) OR Medulloblastoma) OR medulloblastom*) OR PNET*) OR Neuroectodermal tumors, primitive) OR Retinoblastoma) OR retinoblastom*) OR meningioma) OR meningiom*) OR glioma) OR gliom*) OR pediatric oncology) OR paediatric oncology) OR childhood cancer) OR childhood tumor) OR childhood tumors) OR brain tumor*) OR brain tumour*) OR brain neoplasms) OR central nervous system neoplasm) OR central nervous system neoplasms) OR central nervous system tumor*) OR central nervous system tumour*) OR brain cancer*) OR brain neoplasm*) OR intracranial neoplasm*) OR testis neoplasm) OR neoplasm, testicular) OR testicular neoplasm) OR testicular neoplasms) OR testis cancer) OR testicular cancer) OR testis tumor) OR testicular cancer) OR cancer of testis) OR testis neoplasm*) OR testis tumour*) OR testis tumor*) OR leukemia, lymphocytic, acute))) AND ((((((((((((((((((((((((((((((((((((((((((((((((Spermatogenesis) OR gonadal disorder) OR spermiogenesis) OR spermatocytogenesis) OR spermatogenic failure) OR azoospermia) OR oligospermia) OR asthenozoospermia) OR teratozoospermia) OR oligoasthenoteratozoospermia) OR dysspermia) OR normozoospermic) OR semen) OR semen analysis) OR semen quality) OR sperm) OR sperm count) OR sperm motility) OR spermatozoa) OR progeny) OR offspring) OR posterity) OR fertility) OR infertility) OR subfertility) OR reproduction) OR fertilization) OR conception) OR paternity) OR fatherhood) OR parenthood) OR pregnancy outcome) OR fertile) OR infertile) OR subfertile) OR sperm maturation) OR aspermia) OR spermatozoon abnormality) OR germ cell) OR spermatogonia) OR spermatogonial) OR spermatogonium) OR meiosis) OR gonocyte) OR spermatid) OR spermatids) OR follicle stimulating hormone) OR FSH)

**Cisplatin and Sertoli Cell**

(((((((Sertoli) OR anti-mullerian) OR AMH) OR inhibin) OR inhibin B) OR androgen receptor)) AND ((((male[tiab] OR males OR boy OR boys OR boyfriend OR boyhood)) AND ((((((((((((((((((((((alkylating) OR antineoplastic alkylating agents) OR alkylating agents) OR alkylating agent*) OR Carboplatin) OR NSC 241240) OR Carboplat) OR carboplatin*) OR Platinum) OR 41575-94-4) OR Cisplatin) OR Platinum Diamminodichloride) OR Platinol) OR Platidiam) OR Platino) OR NSC-119875) OR Biocisplatinum) OR Cddp) OR Capc) OR cisplatin*) OR cis-DDP 3) OR 15663-27-1)) AND (((((((((((((((((((((((((((((((((((((((((((((((((((((((((((((((((((((((leukemia) OR leukemic*) OR leukaemi*) OR childhood ALL) OR AML) OR lymphoma) OR lymphom*) OR Hodgkin) OR hodgkin*) OR T-cell) OR B-cell) OR non-hodgkin) OR sarcoma) OR sarcom*) OR sarcoma) OR Ewing's) OR Ewing*) OR osteosarcoma) OR osteosarcom*) OR wilms tumor) OR wilms*) OR nephroblastom*) OR neuroblastoma) OR neuroblastom*) OR rhabdomyosarcoma) OR rhabdomyosarcoma*) OR teratoma) OR teratom*) OR hepatoma) OR hepatom*) OR hepatoblastoma) OR hepatoblastom*) OR PNET) OR Medulloblastoma) OR medulloblastom*) OR PNET*) OR Neuroectodermal tumors, primitive) OR Retinoblastoma) OR retinoblastom*) OR meningioma) OR meningiom*) OR glioma) OR gliom*) OR pediatric oncology) OR paediatric oncology) OR childhood cancer) OR childhood tumor) OR childhood tumors) OR brain tumor*) OR brain tumour*) OR brain neoplasms) OR central nervous system neoplasm) OR central nervous system neoplasms) OR central nervous system tumor*) OR central nervous system tumour*) OR brain cancer*) OR brain neoplasm*) OR intracranial neoplasm*) OR testis neoplasm) OR neoplasm, testicular) OR testicular neoplasm) OR testicular neoplasms) OR testis cancer) OR testicular cancer) OR testis tumor) OR testicular cancer) OR cancer of testis) OR testis neoplasm*) OR testis tumour*) OR testis tumor*) OR leukemia, lymphocytic, acute))

**Cisplatin and Leydig Cell**

((((((((((((((((((((((((((((androgen hormone insufficiency) OR leydig cell) OR leydig failure) OR testicular failure) OR interstitial cell failure) OR gonadal failure) OR hypogonadism) OR low testosterone) OR testosterone deficiency) OR leydig cell insufficiency) OR androgen deficiency) OR low testosterone*) OR hypogonadism*) OR leydig cell*) OR testosterone) OR luteinising hormone) OR LH) OR steroidogenesis) OR puberty) OR pubertal) OR testicular volume) OR testis volume) OR tanner stage) OR tanner staging) OR androgen) OR androgens) OR androgenic)) AND ((((male[tiab] OR males OR boy OR boys OR boyfriend OR boyhood)) AND ((((((((((((((((((((((alkylating) OR antineoplastic alkylating agents) OR alkylating agents) OR alkylating agent*) OR Carboplatin) OR NSC 241240) OR Carboplat) OR carboplatin*) OR Platinum) OR 41575-94-4) OR Cisplatin) OR Platinum Diamminodichloride) OR Platinol) OR Platidiam) OR Platino) OR NSC-119875) OR Biocisplatinum) OR Cddp) OR Capc) OR cisplatin*) OR cis-DDP 3) OR 15663-27-1)) AND (((((((((((((((((((((((((((((((((((((((((((((((((((((((((((((((((((((((leukemia) OR leukemic*) OR leukaemi*) OR childhood ALL) OR AML) OR lymphoma) OR lymphom*) OR Hodgkin) OR hodgkin*) OR T-cell) OR B-cell) OR non-hodgkin) OR sarcoma) OR sarcom*) OR sarcoma) OR Ewing's) OR Ewing*) OR osteosarcoma) OR osteosarcom*) OR wilms tumor) OR wilms*) OR nephroblastom*) OR neuroblastoma) OR neuroblastom*) OR rhabdomyosarcoma) OR rhabdomyosarcoma*) OR teratoma) OR teratom*) OR hepatoma) OR hepatom*) OR hepatoblastoma) OR hepatoblastom*) OR PNET) OR Medulloblastoma) OR medulloblastom*) OR PNET*) OR Neuroectodermal tumors, primitive) OR Retinoblastoma) OR retinoblastom*) OR meningioma) OR meningiom*) OR glioma) OR gliom*) OR pediatric oncology) OR paediatric oncology) OR childhood cancer) OR childhood tumor) OR childhood tumors) OR brain tumor*) OR brain tumour*) OR brain neoplasms) OR central nervous system neoplasm) OR central nervous system neoplasms) OR central nervous system tumor*) OR central nervous system tumour*) OR brain cancer*) OR brain neoplasm*) OR intracranial neoplasm*) OR testis neoplasm) OR neoplasm, testicular) OR testicular neoplasm) OR testicular neoplasms) OR testis cancer) OR testicular cancer) OR testis tumor) OR testicular cancer) OR cancer of testis) OR testis neoplasm*) OR testis tumour*) OR testis tumor*) OR leukemia, lymphocytic, acute))
