# Supplementary material for: Impacts of platinum-based chemotherapy on subsequent testicular function and fertility in boys with cancer
Source: Hum Reprod Update. 2020 Sep 16;26(6):874–85. doi: 10.1093/humupd/dmaa041 (PMC7600277; doi:10.1093/humupd/dmaa041)
Supplement: dmaa041_Supplementary_Data [file dmaa041_supplementary_data.zip › dmaa041-suppl_data/Supplementary_Table_SII final.docx]

**Supplementary Table SII** Publications subjected to initial full text screening and that were excluded for not meeting at least one aspect of the inclusion criteria.

| **AUTHOR(S)** | **YEAR** | **TITLE** | **JOURNAL** | **VOLUME** | **PAGES** | **DOI** |
| --- | --- | --- | --- | --- | --- | --- |
| Mackenzie AR | 1966 | The chemotherapy of metastatic seminoma. | The Journal of Urology | 96 | 790-793 | <http://dx.doi.org/10.1016/s0022-5347(17)63351-2> |
| Steinfeld JL, Solomon J, Marsh AA, Hazen JG and Bateman JR | 1966 | Chemical therapy of patients with advanced metastatic germinal tumors. | The Journal of Urology | 96 | 933-940 | <http://dx.doi.org/10.1016/s0022-5347(17)63351-3> |
| Solomon J, Steinfeld JL and Bateman JR | 1967 | Chemotherapy of germinal tumors. | Cancer | 20 | 747-750 | <http://dx.doi.org/10.1016/s0022-5347(17)63351-4> |
| Abrahamova J, Jakoubkova J and Havrankova N | 1970 | Combined chemotherapy of epithelial tumours. | Neoplasma | 17 | 299-310 |  |
| Hanham IW, Newton KA and Westbury G | 1971 | Seventy-five cases of solid tumours treated by a modified quadruple chemotherapy regime. | British Journal of Cancer | 25 | 462-478 | <http://dx.doi.org/10.1038/bjc.1971.59> |
| DeVita VTJ, Canellos GP and Moxley JHr | 1972 | A decade of combination chemotherapy of advanced Hodgkin's disease. | Cancer | 30 | 1495-1504 | [http://dx.doi.org/10.1002/1097-0142(197212)30:6<1495::aid-cncr2820300613>3.0.co;2-i](http://dx.doi.org/10.1002/1097-0142(197212)30:6%3c1495::aid-cncr2820300613%3e3.0.co;2-i) |
| Goldstein DP and Piro AJ | 1972 | Combination chemotherapy in the treatment of germ cell tumors containing choriocarcinoma in males and females. | Surgery, Gynecology & Obstetrics | 134 | 61-66 |  |
| DeVita VT, Arseneau JC, Sherins RJ, Canellos GP and Young RC | 1973 | Intensive chemotherapy for Hodgkin's disease: long-term complications. | National Cancer Institute Monograph | 36 | 447-454 |  |
| Higby DJ, Wallace HJJ and Holland JF | 1973 | Cis-diamminedichloroplatinum (NSC-119875): a phase I study. | Cancer Chemotherapy Reports | 57 | 459-463 |  |
| Hinkes E and Plotkin D | 1973 | Reversible drug-induced sterility in a patient with acute leukemia. | JAMA | 223 | 1490-1491 |  |
| Smith JP | 1973 | Testicular tumors in infants and children. | Urology | 2 | 353-360 | <http://dx.doi.org/10.1016/0090-4295(73)90003-4> |
| Andaloro VAJ and Babott D | 1974 | Testicular involvement in plasma-cell leukemia. | Urology | 3 | 636-638 | <http://dx.doi.org/10.1016/s0090-4295(74)80266-9> |
| Brosman SA, Cohen A and Fay R | 1974 | Rhabdomyosarcoma of testis and spermatic cord in children. | Urology | 3 | 568-572 | <http://dx.doi.org/10.1016/s0090-4295(74)80249-9> |
| Exelby PR | 1974 | Management of embryonal rhabdomyosarcoma in children. | The Surgical Clinics of North America | 54 | 849-857 | <http://dx.doi.org/10.1016/s0039-6109(16)40388-9> |
| Higby DJ, Wallace HJJ, Albert DJ and Holland JF | 1974 | Diaminodichloroplatinum: a phase I study showing responses in testicular and other tumors. | Cancer | 33 | 1215-1219 | [http://dx.doi.org/10.1002/1097-0142(197405)33:5<1219::aid-cncr2820330505>3.0.co;2-u](http://dx.doi.org/10.1002/1097-0142(197405)33:5%3c1219::aid-cncr2820330505%3e3.0.co;2-u) |
| Cox JD | 1975 | Primary malignant germinal tumors of the mediastinum. A study of 24 cases. | Cancer | 36 | 1162-1168 | [http://dx.doi.org/10.1002/1097-0142(197509)36:3<1162::aid-cncr2820360351>3.0.co;2-0](http://dx.doi.org/10.1002/1097-0142(197509)36:3%3c1162::aid-cncr2820360351%3e3.0.co;2-0) |
| Gottlieb JA and Drewinko B | 1975 | Review of the current clinical status of platinum coordination complexes in cancer chemotherapy. | Cancer Chemotherapy Reports | 59 | 621-628 |  |
| Wollner N, Lieberman P, Exelby P, D'angio G, Burchenal J, Fang S and Murphy ML | 1975 | Non-Hodgkin's lymphoma in children: results of treatment with LSA2-L2 protocol. | The British Journal of Cancer. Supplement | 2 | 337-342 |  |
| Pommatau E, Brunat M, Chassard JL and Mayer M | 1976 | The valve of chemotherapy in the treatment of testicular dysembryoplastic tumors. | Journal of Surgical Oncology | 8 | 211-215 | <http://dx.doi.org/10.1002/jso.2930080305> |
| Samuels ML, Lanzotti VJ, Holoye PY, Boyle LE, Smith TL and Johnson DE | 1976 | Combination chemotherapy in germinal cell tumors. | Cancer Treatment Reviews | 3 | 185-204 |  |
| Dekernion JB and Lupu AN | 1977 | The response of metastatic retroperitoneal seminoma to chemotherapy. | The Journal of Urology | 117 | 736-738 | <http://dx.doi.org/10.1016/s0022-5347(17)58605-x> |
| Garrett MJ, Das S, Smith JD and Freedman LS | 1977 | Long term experience with combination chemotherapy in advanced Hodgkin's disease. | Clinical Oncology | 3 | 145-154 |  |
| Shalet SM, Beardwell CG, Twomey JA, Jones PH and Pearson D | 1977 | Endocrine function following the treatment of acute leukemia in childhood. | The Journal of Pediatrics | 90 | 920-923 | <http://dx.doi.org/10.1016/s0022-3476(77)80559-3> |
| Weichselbaum RR, Cassady JR, Jaffe N and Filler R | 1977 | The evolution of combination therapy of genitourinary rhabdomyosarcoma in children: a preliminary report. | International Journal of Radiation Oncology, Biology, Physics | 2 | 267-272 | <http://dx.doi.org/10.1016/0360-3016(77)90084-0> |
|  | 1978 | Testicular disease in acute lymphoblastic leukaemia in childhood. Report on behalf of the Medical Research Council's Working Party on leukaemia in childhood. | British Medical Journal | 1 | 334-338 | <http://dx.doi.org/10.1136/bmj.1.6109.334> |
| Lendon M, Hann IM, Palmer MK, Shalet SM and Jones PH | 1978 | Testicular histology after combination chemotherapy in childhood for acute lymphoblastic leukaemia. | Lancet (London, England) | 2 | 439-441 | <http://dx.doi.org/10.1016/s0140-6736(78)91442-3> |
| Raney RBJ, Hays DM, Lawrence WJ, Soule EH, Tefft M and Donaldson MH | 1978 | Paratesticular rhabdomyosarcoma in childhood. | Cancer | 42 | 729-736 | [http://dx.doi.org/10.1002/1097-0142(197808)42:2<729::aid-cncr2820420246>3.0.co;2-r](http://dx.doi.org/10.1002/1097-0142(197808)42:2%3c729::aid-cncr2820420246%3e3.0.co;2-r) |
| Sherins RJ, Olweny CL and Ziegler JL | 1978 | Gynecomastia and gonadal dysfunction in adolescent boys treated with combination chemotherapy for Hodgkin's disease. | The New England Journal of Medicine | 299 | Dec-16 | <http://dx.doi.org/10.1056/NEJM197807062990103> |
| DeWys WD, Begg C, Slayton R, Hahn RG and Brodsky I | 1979 | Chemotherapy for advanced germinal cell neoplasms: preliminary report of an Eastern Cooperative Oncology Group Study. | Cancer Treatment Reports | 63 | 1675-1680 |  |
|  | 1979 | Pregnancy after chemotherapy for Hodgkin's disease. | Lancet (London, England) | 2 | 93 | <http://dx.doi.org/10.1016/s0140-6736(79)90139-9> |
| Kimball JC and Cangir A | 1979 | Occurrence of testicular metastasis in a child with bilateral retinoblastoma. | Cancer Treatment Reports | 63 | 803-804 |  |
| Merrin CE | 1979 | Treatment of genitourinary tumours with cis-dichlorodiammineplatinum(II): experience in 250 patients. | Cancer Treatment Reports | 63 | 1579-1584 |  |
| Einhorn LH and Williams SD | 1980 | Chemotherapy of disseminated seminoma. | Cancer Clinical Trials | 3 | 307-313 |  |
| Feun LG, Samson MK and Stephens RL | 1980 | Vinblastine (VLB), bleomycin (BLEO), cis-diamminedichloroplatinum (DDP) in disseminated extragonadal germ cell tumors. A Southwest Oncology Group Study. | Cancer | 45 | 2543-2549 | [http://dx.doi.org/10.1002/1097-0142(19800515)45:10<2543::aid-cncr2820451012>3.0.co;2-h](http://dx.doi.org/10.1002/1097-0142(19800515)45:10%3c2543::aid-cncr2820451012%3e3.0.co;2-h) |
| Holbrook CT, Crist WM, Cain W and Bueschen A | 1980 | Successful chemotherapy for childhood metastatic embryonal cell carcinoma of the testicle: a preliminary report. | Medical and Pediatric Oncology | 8 | 75-81 | <http://dx.doi.org/10.1002/mpo.2950080111> |
| Sarna G, Skinner DG, Smith RB, Zighelboim J, Goodnight JE and Feig S | 1980 | cis-Diamminedichloroplatinum(II) alone and in combination in the treatment of testicular and other malignancies. | Cancer Treatment Reports | 64 | 1077-1082 |  |
| van Hoesel QG and Pinedo HM | 1980 | Complete remission of mediastinal germ-cell tumors with cis- dichlorodiammineplatinum(II) combination chemotherapy. | Cancer Treatment Reports | 64 | 319-321 |  |
| Wang C, Ng RP, Chan TK and Todd D | 1980 | Effect of combination chemotherapy on pituitary-gonadal function in patients with lymphoma and leukemia. | Cancer | 45 | 2030-2037 | [http://dx.doi.org/10.1002/1097-0142(19800415)45:8<2030::aid-cncr2820450807>3.0.co;2-s](http://dx.doi.org/10.1002/1097-0142(19800415)45:8%3c2030::aid-cncr2820450807%3e3.0.co;2-s) |
| Brodeur GM, Howarth CB, Pratt CB, Caces J and Hustu HO | 1981 | Malignant germ cell tumors in 57 children and adolescents. | Cancer | 48 | 1890-1898 | [http://dx.doi.org/10.1002/1097-0142(19811015)48:8<1890::aid-cncr2820480830>3.0.co;2-d](http://dx.doi.org/10.1002/1097-0142(19811015)48:8%3c1890::aid-cncr2820480830%3e3.0.co;2-d) |
| Duffner PK, Cohen ME, Heffner RR and Freeman AI | 1981 | Primitive neuroectodermal tumors of childhood. An approach to therapy. | Journal of NeuroSurgery | 55 | 376-381 | <http://dx.doi.org/10.3171/jns.1981.55.3.0376> |
| Kirshner JJ, Ginsberg SJ, Fitzpatrick AV and Comis RL | 1981 | Treatment of a primary intracranial germ cell tumor with systemic chemotherapy. | Medical and Pediatric Oncology | 9 | 361-365 | <http://dx.doi.org/10.1002/mpo.2950090408> |
| Monfardini S | 1981 | Chemotherapy of testicular carcinomas. | AntiCancer Research | 1 | 309-312 |  |
| Vugrin D, Whitemore WJJ and Batata M | 1981 | Chemotherapy of disseminated seminoma with combination of cis- diamminedichloroplatinum (II) and cyclophosphamide. | Cancer Clinical Trials | 4 | 423-427 |  |
|  | 1981 | Leydig cell dysfunction after combination chemotherapy. | Lancet (London, England) | 2 | 529 | <http://dx.doi.org/10.1016/s0140-6736(81)90915-6> |
| Arshad RR, Woo SY, Abbassi V, Hoy GR and Sinks LF | 1982 | Virilizing hepatoblastoma: precocious sexual development and partial response of pulmonary metastases to cis-platinum. | CA: a Cancer Journal for Clinicians | 32 | 293-300 | <http://dx.doi.org/10.3322/canjclin.32.5.293> |
| Economou JS, Trump DL, Holmes EC and Eggleston JE | 1982 | Management of primary germ cell tumors of the mediastinum. | The Journal of Thoracic and Cardiovascular Surgery | 83 | 643-649 |  |
| Hainsworth JD, Einhorn LH, Williams SD, Stewart M and Greco FA | 1982 | Advanced extragonadal germ-cell tumors. Successful treatment with combination chemotherapy. | Annals of Internal Medicine | 97 | 7-Nov | <http://dx.doi.org/10.7326/0003-4819-97-1-7> |
| Levi JA, Aroney RS and Dalley DN | 1982 | Significant factors in the optimal management of advanced stage germ cell carcinoma. | Australian and New Zealand Journal of Medicine | 12 | 147-152 | <http://dx.doi.org/10.1111/j.1445-5994.1982.tb02447.x> |
| Mortimer J, Bukowski RM, Montie J, Hewlett JS and Livingston RB | 1982 | VP16-213, cisplatinum, and adriamycin salvage therapy of refractory and/or recurrent nonseminomatous germ cell neoplasms. | Cancer Chemotherapy and pharmacology | 7 | 215-218 | <http://dx.doi.org/10.1007/bf00254553> |
| Ward Platt MP, Mott MG and Eden OB | 1982 | Intermittent combined chemotherapy with doxorubicin in recurrent childhood acute lymphoblastic leukemia. | Medical and Pediatric Oncology | 10 | 563-568 | <http://dx.doi.org/10.1002/mpo.2950100606> |
| Waxman JH, Terry YA, Wrigley PF, Malpas JS, Rees LH, Besser GM and Lister TA | 1982 | Gonadal function in Hodgkin's disease: long-term follow-up of chemotherapy. | British Medical Journal (Clinical Research ed.) | 285 | 1612-1613 | <http://dx.doi.org/10.1136/bmj.285.6355.1612> |
| Whitehead E, Shalet SM, Jones PH, Beardwell CG and Deakin DP | 1982 | Gonadal function after combination chemotherapy for Hodgkin's disease in childhood. | Archives of Disease in Cildhood | 57 | 287-291 | <http://dx.doi.org/10.1136/adc.57.4.287> |
| Williams SD and Einhorn LH | 1982 | Cis-platinum in the treatment of testicular and other cancers. | Advances in Internal Medicine | 27 | 531-545 |  |
| Daugaard G, Rorth M and Hansen HH | 1983 | Therapy of extragonadal germ-cell tumors. | European Journal of Cancer & Clinical Oncology | 19 | 895-899 | <http://dx.doi.org/10.1016/0277-5379(83)90054-8> |
| Garnick MB, Canellos GP and Richie JP | 1983 | Treatment and surgical staging of testicular and primary extragonadal germ cell cancer. | JAMA | 250 | 1733-1741 |  |
| Lederman GS, Garnick MB, Canellos GP and Richie JP | 1983 | Chemotherapy of refractory germ cell cancer with Etoposide. | Journal of Clinical Oncology : official Journal of the American Society of Clinical Oncology | 1 | 706-709 | <http://dx.doi.org/10.1200/JCO.1983.1.11.706> |
| Mauch PM, Weinstein H, Botnick L, Belli J and Cassady JR | 1983 | An evaluation of long-term survival and treatment complications in children with Hodgkin's disease. | Cancer | 51 | 925-932 | [http://dx.doi.org/10.1002/1097-0142(19830301)51:5<925::aid-cncr2820510527>3.0.co;2-o](http://dx.doi.org/10.1002/1097-0142(19830301)51:5%3c925::aid-cncr2820510527%3e3.0.co;2-o) |
| Ragni G, Lombardi C, Santoro A, Bestetti O and Wyssling H | 1983 | Male infertility caused by the chemotherapy of Hodgkin's disease: MOPP versus ABVD. | Acta Europaea Fertilitatis | 14 | 221-222 |  |
| Reddel RR, Thompson JF, Raghavan D, Tattersall MH, Levi JA, Coupland GA, Fox RM, Coates AS, Woods RL and Ng AB | 1983 | Surgery in patients with advanced germ cell malignancy following a clinical partial response to chemotherapy. | Journal of Surgical Oncology | 23 | 223-227 | <http://dx.doi.org/10.1002/jso.2930230403> |
| Thomas PR, Griffith KD, Fineberg BB, Perez CA and Land VJ | 1983 | Late effects of treatment for Wilms' tumor. | International Journal of Radiation Oncology, Biology, Physics | 9 | 651-657 | <http://dx.doi.org/10.1016/0360-3016(83)90230-4> |
| Cornbleet MA, Leonard RC and Smyth JF | 1984 | High-dose alkylating agent therapy: a review of clinical experiences. | Cancer drug delivery | 1 | 227-238 |  |
| Dein RA, Mennuti MT, Kovach P and Gabbe SG | 1984 | The reproductive potential of young men and women with Hodgkin's disease. | Obstetrical & Gynecological Survey | 39 | 474-482 |  |
| Fossa SD, Klepp O, Ous S, Lien H, Stenwig JT, Abeler V, Eliassen G and Host H | 1984 | Multi-modality treatment in males with advanced malignant germ cell tumours. II. Experience with surgery and radiotherapy following cis- platinum-based chemotherapy. | Scandinavian Journal of Urology and Nephrology | 18 | 21-26 | <http://dx.doi.org/10.3109/00365598409182159> |
|  | 1984 | Treatment of extragonadal germ cell tumors. | Journal of Clinical Oncology : official Journal of the American Society of Clinical Oncology | 2 | 712-713 | <http://dx.doi.org/10.1200/jco.1984.2.6.712> |
| Johnson DH, Hainsworth JD, Linde RB and Greco FA | 1984 | Testicular function following combination chemotherapy with cis-platin, vinblastine, and bleomycin. | Medical and Pediatric Oncology | 12 | 233-238 | <http://dx.doi.org/10.1002/mpo.2950120403> |
| Klepp O, Fossa SD, Ous S, Lien H, Stenwig JT, Abeler V, Eliassen G and Host H | 1984 | Multi-modality treatment of advanced malignant germ cell tumours in males. I. Experience with cis-platinum-based combination chemotherapy. | Scandinavian Journal of Urology and Nephrology | 18 | 13-19 | <http://dx.doi.org/10.3109/00365598409182158> |
| Phillips GL, Herzig RH, Lazarus HM, Fay JW, Wolff SN, Mill WB, Lin H, Thomas PR, Glasgow GP and Shina DC | 1984 | Treatment of resistant malignant lymphoma with cyclophosphamide, total body irradiation, and transplantation of cryopreserved autologous marrow. | The New England Journal of Medicine | 310 | 1557-1561 | <http://dx.doi.org/10.1056/NEJM198406143102403> |
| Specht L, Geisler C, Hansen MM and Skakkebaek NE | 1984 | Testicular function in young men in long-term remission after treatment for the early stages of Hodgkin's disease. | Scandinavian Journal of Haematology | 33 | 356-362 |  |
| Bosl GJ, Yagoda A, Golbey RB, Whitmore WJ, Herr H, Sogani P, Morse M, Vogelzang N and MacDonald G | 1985 | Role of etoposide-based chemotherapy in the treatment of patients with refractory or relapsing germ cell tumors. | The American Journal of Medicine | 78 | 423-428 | <http://dx.doi.org/10.1016/0002-9343(85)90333-x> |
| Chun H, Bosl GJ and Golbey RB | 1985 | Phase II trial of 1,2-diaminocyclohexane-(4-carboxyphthalato) platinum(II) in patients with refractory germ cell tumors. | Cancer Treatment Reports | 69 | 459-460 |  |
| Guazzieri S, Ferro G, Lembo A, Merlo F, Sperandio P and Pagano F | 1985 | Our experience in regard to the relationship between the size of retroperitoneal lymph node metastases and the effect of chemotherapy in non seminomatous germ cell tumors. | Progress in Clinical and Biological Research | 203 | 457-462 |  |
| Hainsworth JD, Williams SD, Einhorn LH, Birch R and Greco FA | 1985 | Successful treatment of resistant germinal neoplasms with VP-16 and cisplatin: results of a Southeastern Cancer Study Group trial. | Journal of Clinical Oncology : official Journal of the American Society of Clinical Oncology | 3 | 666-671 | <http://dx.doi.org/10.1200/JCO.1985.3.5.666> |
| Israel A, Bosl GJ, Golbey RB, Whitmore WJ and Martini N | 1985 | The results of chemotherapy for extragonadal germ-cell tumors in the cisplatin era: the Memorial Sloan-Kettering Cancer Center experience (1975 to 1982). | Journal of Clinical Oncology : official Journal of the American Society of Clinical Oncology | 3 | 1073-1078 | <http://dx.doi.org/10.1200/JCO.1985.3.8.1073> |
| Kreuser ED, Jaeger N, Altwein JE, Egghart G, Hartlapp J, Gaus W and Schreml W | 1985 | Bulky germinal tumors: comparison of different induction regimens and significance of residual disease. | European Urology | 11 | 163-169 | <http://dx.doi.org/10.1159/000472483> |
| Newlands ES | 1985 | VP-16 in combinations for first-line treatment of malignant germ-cell tumors and gestational choriocarcinoma. | Seminars in Oncology | 12 | 37-41 |  |
| Peckham MJ and Hendry WF | 1985 | Clinical stage II non-seminomatous germ cell testicular tumours. Results of management by primary chemotherapy. | British Journal of Urology | 57 | 763-768 | <http://dx.doi.org/10.1111/j.1464-410x.1985.tb07050.x> |
| Peckham MJ, Horwich A and Hendry WF | 1985 | Advanced seminoma: treatment with cis-platinum-based combination chemotherapy or carboplatin (JM8). | British Journal of Cancer | 52 | Jul-13 | <http://dx.doi.org/10.1038/bjc.1985.141> |
| Rosenberg SA and Kaplan HS | 1985 | The evolution and summary results of the Stanford randomized clinical trials of the management of Hodgkin's disease: 1962-1984. | International Journal of Radiation Oncology, Biology, Physics | 11 | May-22 | <http://dx.doi.org/10.1016/0360-3016(85)90357-8> |
| Trump DL and Hortvet L | 1985 | Etoposide and very high dose cisplatin: salvage therapy for patients with advanced germ cell neoplasms. | Cancer Treatment Reports | 69 | 259-261 |  |
| Wilkinson PM | 1985 | Chemotherapy for non-seminomatous germ-cell tumours. | Journal of the Royal Society of Medicine | 78 Suppl 6 | 43-47 |  |
| Bosl GJ, Gluckman R, Geller NL, Golbey RB, Whitmore WFJ, Herr H, Sogani P, Morse M, Martini N and Bains M | 1986 | VAB-6: an effective chemotherapy regimen for patients with germ-cell tumors. | Journal of Clinical Oncology : official Journal of the American Society of Clinical Oncology | 4 | 1493-1499 | <http://dx.doi.org/10.1200/JCO.1986.4.10.1493> |
| Daugaard G and Rrth M | 1986 | High-dose cisplatin and VP-16 with bleomycin, in the management of advanced metastatic germ cell tumors. | European Journal of Cancer & Clinical Oncology | 22 | 477-485 | <http://dx.doi.org/10.1016/0277-5379(86)90115-x> |
| Einhorn LH | 1986 | VP 16 plus ifosfamide plus cisplatin as salvage therapy in refractory testicular cancer. | Cancer Chemotherapy and Pharmacology | 18  Suppl 2 | S45-50 | <http://dx.doi.org/10.1007/bf00647451> |
| Flamant F, Nihoul-Fekete C, Patte C and Lemerle J | 1986 | Optimal treatment of clinical stage I yolk sac tumor of the testis in children. | Journal of Pediatric Surgery | 21 | 108-111 | <http://dx.doi.org/10.1016/s0022-3468(86)80059-8> |
| Hall KS, Fossa SD and Aas M | 1986 | High-dose cis-platinum combination chemotherapy in advanced nonseminomatous malignant germ cell tumours with emphasis on nephrotoxicity. | Cancer Chemotherapy and Pharmacology | 18 | 74-77 | <http://dx.doi.org/10.1007/bf00253069> |
| Hansen SW, Daugaard G and Rorth M | 1986 | Treatment of persistent or relapsing advanced germ cell neoplasms with cisplatin, etoposide and bleomycin. | European Journal of Cancer & Clinical Oncology | 22 | 595-599 | <http://dx.doi.org/10.1016/0277-5379(86)90049-0> |
| Ise T, Kishi K, Imashuku S, Tsukada M, Tsukimoto I, Tsujino G, Bessho F, Tanaka H, Miyazaki S and Sakurai M | 1986 | Testicular histology and function following long-term chemotherapy of acute leukemia in children and outcome of the patients who received testicular biopsy. | The American Journal of Pediatric Hematology/Oncology | 8 | 288-293 |  |
| Kida Y, Kobayashi T, Yoshida J, Kato K and Kageyama N | 1986 | Chemotherapy with cisplatin for AFP-secreting germ-cell tumors of the central nervous system. | Journal of NeuroSurgery | 65 | 470-475 | <http://dx.doi.org/10.3171/jns.1986.65.4.0470> |
| Koziner B, Myers J, Cirrincione C, Redman J, Cunningham I, Caravelli J, Nisce LZ, McCormick B, Straus DJ and Mertelsmann R | 1986 | Treatment of stages I and II Hodgkin's disease with three different therapeutic modalities. | The American Journal of Medicine | 80 | 1067-1078 | <http://dx.doi.org/10.1016/0002-9343(86)90667-4> |
| Lederman GS and Garnick MB | 1986 | Possible benefit of doxorubicin treatment in patients with refractory germ cell cancer. | Cancer | 58 | 2393-2398 | [http://dx.doi.org/10.1002/1097-0142(19861201)58:11<2393::aid-cncr2820581107>3.0.co;2-i](http://dx.doi.org/10.1002/1097-0142(19861201)58:11%3c2393::aid-cncr2820581107%3e3.0.co;2-i) |
| Loehrer PJ, Mandelbaum I, Hui S, Clark S, Einhorn LH, Williams SD and Donohue JP | 1986 | Resection of thoracic and abdominal teratoma in patients after cisplatin- based chemotherapy for germ cell tumor. Late results. | The Journal of Thoracic and Cardiovascular Surgery | 92 | 676-683 |  |
| Logothetis CJ, Samuels ML, Selig DE, Ogden S, Dexeus F, Swanson D, Johnson D and von Eschenbach A | 1986 | Cyclic chemotherapy with cyclophosphamide, doxorubicin, and cisplatin plus vinblastine and bleomycin in advanced germinal tumors. Results with 100 patients. | The American Journal of Medicine | 81 | 219-228 | <http://dx.doi.org/10.1016/0002-9343(86)90255-x> |
| Newlands ES, Bagshawe KD, Begent RH, Rustin GJ, Crawford SM and Holden L | 1986 | Current optimum management of anaplastic germ cell tumours of the testis and other sites. | British Journal of Urology | 58 | 307-314 | <http://dx.doi.org/10.1111/j.1464-410x.1986.tb09061.x> |
| Pinkerton CR, Pritchard J and Spitz L | 1986 | High complete response rate in children with advanced germ cell tumors using cisplatin-containing combination chemotherapy. | Journal of Clinical Oncology : official Journal of the American Society of Clinical Oncology | 4 | 194-199 | <http://dx.doi.org/10.1200/JCO.1986.4.2.194> |
| Pizzocaro G, Salvioni R, Piva L, Zanoni F, Milani A and Faustini M | 1986 | Cisplatin combination chemotherapy in advanced seminoma. | Cancer | 58 | 1625-1629 | [http://dx.doi.org/10.1002/1097-0142(19861015)58:8<1625::aid-cncr2820580807>3.0.co;2-b](http://dx.doi.org/10.1002/1097-0142(19861015)58:8%3c1625::aid-cncr2820580807%3e3.0.co;2-b) |
| Rustin GJ, Newlands ES, Bagshawe KD, Begent RH and Crawford SM | 1986 | Successful management of metastatic and primary germ cell tumors in the brain. | Cancer | 57 | 2108-2113 | [http://dx.doi.org/10.1002/1097-0142(19860601)57:11<2108::aid-cncr2820571103>3.0.co;2-z](http://dx.doi.org/10.1002/1097-0142(19860601)57:11%3c2108::aid-cncr2820571103%3e3.0.co;2-z) |
| Sanders JE, Pritchard S, Mahoney P, Amos D, Buckner CD, Witherspoon RP, Deeg HJ, Doney KC, Sullivan KM and Appelbaum FR | 1986 | Growth and development following marrow transplantation for leukemia. | Blood | 68 | 1129-1135 |  |
| Allen JC, Kim JH and Packer RJ | 1987 | Neoadjuvant chemotherapy for newly diagnosed germ-cell tumors of the central nervous system. | Journal of NeuroSurgery | 67 | 65-70 | <http://dx.doi.org/10.3171/jns.1987.67.1.0065> |
| Antman K, Eder JP and Frei Er | 1987 | High-dose chemotherapy with bone marrow support for solid tumors. | Important Advances in Oncology |  | 221-235 |  |
| Atkinson CH, Horwich A and Peckham MJ | 1987 | Methotrexate for relapse of metastatic non-seminomatous germ-cell tumours. | Medical Oncology and Tumor Pharmacotherapy | 4 | 33-37 |  |
| Bennett JM, Moloney WC, Greene MH and Boice JDJ | 1987 | Acute myeloid leukemia and other myelopathic disorders following treatment with alkylating agents. | Hematologic Pathology | 1 | 99-104 |  |
| Bosl GJ and Bajorunas D | 1987 | Pituitary and testicular hormonal function after treatment for germ cell tumours. | International Journal of Andrology | 10 | 381-384 |  |
| Bosl GJ, Geller NL, Vogelzang NJ, Carey R, Auman J, Whitmore WF, Herr H, Morse M, Sogani P and Chan E | 1987 | Alternating cycles of etoposide plus cisplatin and VAB-6 in the treatment of poor-risk patients with germ cell tumors. | Journal of Clinical Oncology : official Journal of the American Society of Clinical Oncology | 5 | 436-440 | <http://dx.doi.org/10.1200/JCO.1987.5.3.436> |
| Byrne J, Mulvihill JJ, Myers MH, Connelly RR, Naughton MD, Krauss MR, Steinhorn SC, Hassinger DD, Austin DF and Bragg K | 1987 | Effects of treatment on fertility in long-term survivors of childhood or adolescent cancer. | The New England Journal of Medicine | 317 | 1315-1321 | <http://dx.doi.org/10.1056/NEJM198711193172104> |
| Daugaard G, Hansen HH and Rorth M | 1987 | Management of advanced metastatic germ cell tumours. | International Journal of Andrology | 10 | 319-324 |  |
| Fossa SD, Borge L, Aass N, Johannessen NB, Stenwig AE and Kaalhus O | 1987 | The treatment of advanced metastatic seminoma: experience in 55 cases. | Journal of Clinical Oncology : official Journal of the American Society of Clinical Oncology | 5 | 1071-1077 | <http://dx.doi.org/10.1200/JCO.1987.5.7.1071> |
| Horwich A, Easton D, Husband J, Nicholas D and Peckham MJ | 1987 | Prognosis following chemotherapy for metastatic malignant teratoma. | British Journal of Urology | 59 | 578-583 | <http://dx.doi.org/10.1111/j.1464-410x.1987.tb04881.x> |
|  | 1987 | Cyclic chemotherapy with cyclophosphamide, doxorubicin, and cisplatin plus vinblastine and bleomycin in advanced germinal tumors. | The American Journal of Medicine | 82 | 575-576 | <http://dx.doi.org/10.1016/0002-9343(87)90475-x> |
| Logothetis CJ, Samuels ML, Ogden SL, Dexeus FH and Chong CD | 1987 | Cyclophosphamide and sequential cisplatin for advanced seminoma: long- term followup in 52 patients. | The Journal of Urology | 138 | 789-794 | <http://dx.doi.org/10.1016/s0022-5347(17)43376-3> |
| Newlands ES, Bagshawe KD, Begent RH, Rustin GJ, Crawford SM and Holden L | 1987 | Treatment of patients with poor prognosis anaplastic germ cell tumours (AGCT) of the testis and other sites. | International Journal of Andrology | 10 | 301-309 |  |
| Ozols RF | 1987 | Treatment of poor prognosis germ cell tumours with high dose cisplatin regimens. | International Journal of Andrology | 10 | 291-300 |  |
| Bosl GJ, Geller NL, Bajorin D, Leitner SP, Yagoda A, Golbey RB, Scher H, Vogelzang NJ, Auman J and Carey R | 1988 | A randomized trial of etoposide + cisplatin versus vinblastine + bleomycin + cisplatin + cyclophosphamide + dactinomycin in patients with good-prognosis germ cell tumors. | Journal of Clinical Oncology : official Journal of the American Society of Clinical Oncology | 6 | 1231-1238 | <http://dx.doi.org/10.1200/JCO.1988.6.8.1231> |
| Bukowski RM, Smith GW and Montie JE | 1988 | Combination chemotherapy including VP-16 for poor prognosis germ cell neoplasms. | Urology | 31 | 403-407 | <http://dx.doi.org/10.1016/0090-4295(88)90735-2> |
| Clayton PE, Shalet SM, Price DA and Campbell RH | 1988 | Testicular damage after chemotherapy for childhood brain tumors. | The Journal of Pediatrics | 112 | 922-926 | <http://dx.doi.org/10.1016/s0022-3476(88)80218-x> |
| Cullen MH, Harper PG, Woodroffe CM, Kirkbride P and Clarke J | 1988 | Chemotherapy for poor risk germ cell tumours. An independent evaluation of the POMB/ACE regime. | British Journal of Urology | 62 | 454-460 | <http://dx.doi.org/10.1111/j.1464-410x.1988.tb04396.x> |
| Ghosn M, Droz JP, Theodore C, Pico JL, Baume D, Spielmann M, Ostronoff M, Moran A, Salloum E and Kramar A | 1988 | Salvage chemotherapy in refractory germ cell tumors with etoposide (VP-16) plus ifosfamide plus high-dose cisplatin. A VIhP regimen. | Cancer | 62 | 24-27 | [http://dx.doi.org/10.1002/1097-0142(19880701)62:1<24::aid-cncr2820620106>3.0.co;2-y](http://dx.doi.org/10.1002/1097-0142(19880701)62:1%3c24::aid-cncr2820620106%3e3.0.co;2-y) |
| Graham J, Harding M, Mill L, Kerr DJ, Rankin E, Calman KC and Kaye SB | 1988 | Results of treatment of non seminomatous germ cell tumours; 122 consecutive cases in the West of Scotland, 1981-1985. | British Journal of Cancer | 57 | 182-185 | <http://dx.doi.org/10.1038/bjc.1988.38> |
| Jaffe N, Sullivan MP, Ried H, Boren H, Marshall R, Meistrich M, Maor M and da Cunha M | 1988 | Male reproductive function in long-term survivors of childhood cancer. | Medical and Pediatric Oncology | 16 | 241-247 |  |
| Levi JA, Thomson D, Sandeman T, Tattersall M, Raghavan D, Byrne M, Gill G, Harvey V, Burns I and Snyder R | 1988 | A prospective study of cisplatin-based combination chemotherapy in advanced germ cell malignancy: role of maintenance and long-term follow- up. | Journal of Clinical Oncology : official Journal of the American Society of Clinical Oncology | 6 | 1154-1160 | <http://dx.doi.org/10.1200/JCO.1988.6.7.1154> |
| Livesey EA and Brook CG | 1988 | Gonadal dysfunction after treatment of intracranial tumours. | Archives of Disease in Childhood | 63 | 495-500 | <http://dx.doi.org/10.1136/adc.63.5.495> |
| McLeod DG, Taylor HG, Skoog SJ, Knight RD, Dawson NA and Waxman JA | 1988 | Extragonadal germ cell tumors. Clinicopathologic findings and treatment experience in 12 patients. | Cancer | 61 | 1187-1191 | [http://dx.doi.org/10.1002/1097-0142(19880315)61:6<1187::aid-cncr2820610622>3.0.co;2-8](http://dx.doi.org/10.1002/1097-0142(19880315)61:6%3c1187::aid-cncr2820610622%3e3.0.co;2-8) |
| Mulder PO, de Vries EG, Koops HS, Splinter T, Maas A, van der Geest S, Mulder NH and Sleijfer DT | 1988 | Chemotherapy with maximally tolerable doses of VP 16-213 and cyclophosphamide followed by autologous bone marrow transplantation for the treatment of relapsed or refractory germ cell tumors. | European Journal of Cancer & Clinical Oncology | 24 | 675-679 | <http://dx.doi.org/10.1016/0277-5379(88)90298-2> |
| Rivkees SA and Crawford JD | 1988 | The relationship of gonadal activity and chemotherapy-induced gonadal damage. | JAMA | 259 | 2123-2125 |  |
| Roth BJ, Greist A, Kubilis PS, Williams SD and Einhorn LH | 1988 | Cisplatin-based combination chemotherapy for disseminated germ cell tumors: long-term follow-up. | Journal of Clinical Oncology : official Journal of the American Society of Clinical Oncology | 6 | 1239-1247 | <http://dx.doi.org/10.1200/JCO.1988.6.8.1239> |
| Tsatsoulis A, Shalet SM, Robertson WR, Morris ID, Burger HG and De Kretser DM | 1988 | Plasma inhibin levels in men with chemotherapy-induced severe damage to the seminiferous epithelium. | Clinical Endocrinology | 29 | 659-665 |  |
| Walker RW and Allen JC | 1988 | Cisplatin in the treatment of recurrent childhood primary brain tumors. | Journal of Clinical Oncology : official Journal of the American Society of Clinical Oncology | 6 | 62-66 | <http://dx.doi.org/10.1200/JCO.1988.6.1.62> |
| Weiss RB, Stablein DM, Muggia FM, Einhorn LH, Golbey RB and DeWys WD | 1988 | Toxicity comparisons between two chemotherapy regimens as adjuvant or salvage treatment in nonseminomatous testicular cancer. | Cancer | 62 | 18-23 | [http://dx.doi.org/10.1002/1097-0142(19880701)62:1<18::aid-cncr2820620105>3.0.co;2-q](http://dx.doi.org/10.1002/1097-0142(19880701)62:1%3c18::aid-cncr2820620105%3e3.0.co;2-q) |
| Wilkinson PM, Read G and Magee B | 1988 | The treatment of advanced seminoma with chemotherapy and radiotherapy. | British Journal of Cancer | 57 | 100-104 | <http://dx.doi.org/10.1038/bjc.1988.18> |
| Aubier F, Flamant F, Brauner R, Caillaud JM, Chaussain JM and Lemerle J | 1989 | Male gonadal function after chemotherapy for solid tumors in childhood. | Journal of Clinical Oncology : official Journal of the American Society of Clinical Oncology | 7 | 304-309 | <http://dx.doi.org/10.1200/JCO.1989.7.3.304> |
| Colombat P, Linassier C, Binet C, Desbois I, Chazard M, Reisenleiter M, Haillot O and Lamagnere JP | 1989 | High dose chemotherapy including vepeside, cyclophosphamide and carboplatin with autologous bone marrow transplantation in one case of chemoresistant testicular cancer. | BioMedicine & Pharmacotherapy = Biomedecine & Phrmacotherapie | 43 | 455-456 |  |
| Diez B and Richard L | 1989 | Malignant germ cell sacrococcygeal tumors in children. Improved prognosis after introduction of cisplatin-containing multiple drug treatment. | Acta Oncologica (Stockholm, Sweden) | 28 | 249-251 | <http://dx.doi.org/10.3109/02841868909111257> |
| Droz JP, Ribrag V, Ghosn M, Guimares J, Theodore C, Azab M, Ostronoff M, Mahjoubi M, Moran A and Pico J | 1989 | Phase II trial with etoposide (VP16) plus ifosfamide plus high-dose cisplatin (VIhP regimen) in refractory germ cell tumors. | Progress in Clinical and Biological Research | 303 | 739-747 |  |
| Fernandes ET, Etcubanas E, Rao BN, Kumar AP, Thompson EI and Jenkins JJ | 1989 | Two decades of experience with testicular tumors in children at St Jude Children's Research Hospital. | Journal of Pediatric Surgery | 24 | 677-81; discussion 682 | <http://dx.doi.org/10.1016/s0022-3468(89)80718-3> |
| Gobel U, Calaminus G, Haas RJ, Jurgens H, Niethammer D, Ritter J, Spaar HJ and Harms D | 1989 | Combination chemotherapy in malignant non-seminomatous germ-cell tumors: results of a cooperative study of the German Society of Pediatric Oncology (MAKEI 83). | Cancer Chemotherapy and Pharmacology | 24 Suppl 1 | S34-9 | <http://dx.doi.org/10.1007/bf00253238> |
| Hayashi Y, Ohi R, Konno T, Tsuchiya S, Muraosa Y, Yaoita S and Watanabe T | 1989 | Effect of VAB-6 combination chemotherapy on malignant germ cell tumours in childhood. | Zeitschrift fur Kinderchirurgie : organ der Deutschen, der Schweizerischen und der Osterreichischen Gesellschaft fur Kinderchirurgie = Surgery in infancy and childhood | 44 | 208-212 | <http://dx.doi.org/10.1055/s-2008-1043236> |
| Hijiya N, Horikawa R, Matsushita T, Yamaguchi M and Noda E | 1989 | Malignant mediastinal germ-cell tumors in childhood: a report of two cases achieving long-term Disease-free survival. | The American Journal of Pediatric Hematology/Oncology | 11 | 437-440 |  |
| Hitchins RN, Newlands ES, Smith DB, Begent RH, Rustin GJ and Bagshawe KD | 1989 | Long-term outcome in patients with germ cell tumours treated with POMB/ACE chemotherapy: comparison of commonly used classification systems of good and poor prognosis. | British Journal of Cancer | 59 | 236-242 | <http://dx.doi.org/10.1038/bjc.1989.48> |
| Horwich A, Dearnaley DP, Duchesne GM, Williams M, Brada M and Peckham MJ | 1989 | Simple nontoxic treatment of advanced metastatic seminoma with carboplatin. | Journal of Clinical Oncology : official Journal of the American Society of Clinical Oncology | 7 | 1150-1156 | <http://dx.doi.org/10.1200/JCO.1989.7.8.1150> |
| Mackintosh JF, Hawson GA, Johnston NG and Matar KS | 1989 | Cisplatinum-based chemotherapy in malignant mediastinal teratoma. | The Australian and New Zealand Journal of Surgery | 59 | 399-404 | <http://dx.doi.org/10.1111/j.1445-2197.1989.tb01594.x> |
| Mann JR, Pearson D, Barrett A, Raafat F, Barnes JM and Wallendszus KR | 1989 | Results of the United Kingdom Children's Cancer Study Group's malignant germ cell tumor studies. | Cancer | 63 | 1657-1667 | [http://dx.doi.org/10.1002/1097-0142(19900501)63:9<1657::aid-cncr2820630902>3.0.co;2-8](http://dx.doi.org/10.1002/1097-0142(19900501)63:9%3c1657::aid-cncr2820630902%3e3.0.co;2-8) |
| Shebib S, Sabbah RS, Sackey K, Akhtar M and Aur RJ | 1989 | Endodermal sinus (yolk sac) tumor in infants and children. A clinical and pathologic study: an 11 year review. | The American Journal of Pediatric Hematology/Oncology | 11 | 36-39 |  |
| Dearnaley DP, A'Hern RP, Whittaker S and Bloom HJ | 1990 | Pineal and CNS germ cell tumors: Royal Marsden Hospital experience 1962-1987. | International Journal of Radiation Oncology, Biology, Physics | 18 | 773-781 | <http://dx.doi.org/10.1016/0360-3016(90)90396-2> |
| Falkson G, Chasen MR and Falkson HC | 1990 | Ifosfamide and mesna in combination with other cytostatic drugs in the treatment of patients with advanced cancer. | Investigational New drugs | 8 | 215-219 |  |
| Gobel U, Haas RJ, Calaminus G, Bamberg M, Bokkerink EB, Engert J, Gadner H, Jurgens H, Spaar HJ and Weissach L | 1990 | Treatment of germ cell tumors in children: results of European trials for testicular and non-testicular primary sites. | Critical Reviews in Oncology/Hematology | 10 | 89-98 |  |
| Huddart SN, Mann JR, Gornall P, Pearson D, Barrett A, Raafat F, Barnes JM and Wallendsus KR | 1990 | The UK Children's Cancer Study Group: testicular malignant germ cell tumours 1979-1988. | Journal of Pediatric Surgery | 25 | 406-410 | <http://dx.doi.org/10.1016/0022-3468(90)90381-i> |
| Kotake T and Miki T | 1990 | Combination salvage chemotherapy using cisplatin and teniposide for patients with refractory germinal testicular tumors. | Cancer Chemotherapy and Pharmacology | 27 | 85-88 | <http://dx.doi.org/10.1007/bf00689088> |
| Kovnar EH, Kellie SJ, Horowitz ME, Sanford RA, Langston JW, Mulhern RK, Jenkins JJ, Douglass EC, Etcubanas EE and Fairclough DL | 1990 | Preirradiation cisplatin and etoposide in the treatment of high-risk medulloblastoma and other malignant embryonal tumors of the central nervous system: a phase II study. | Journal of Clinical Oncology : official Journal of the American Society of Clinical Oncology | 8 | 330-336 | <http://dx.doi.org/10.1200/JCO.1990.8.2.330> |
| Lefkowitz IB, Packer RJ, Siegel KR, Sutton LN, Schut L and Evans AE | 1990 | Results of treatment of children with recurrent medulloblastoma/primitive neuroectodermal tumors with lomustine, cisplatin, and vincristine. | Cancer | 65 | 412-417 | [http://dx.doi.org/10.1002/1097-0142(19900201)65:3<412::aid-cncr2820650306>3.0.co;2-4](http://dx.doi.org/10.1002/1097-0142(19900201)65:3%3c412::aid-cncr2820650306%3e3.0.co;2-4) |
| Miller JC and Einhorn LH | 1990 | Phase II study of daily oral etoposide in refractory germ cell tumors. | Seminars in Oncology | 17 | 36-39 |  |
| Motzer RJ, Cooper K, Geller NL, Bajorin DF, Dmitrovsky E, Herr H, Morse M, Fair W, Sogani P and Russo P | 1990 | The role of ifosfamide plus cisplatin-based chemotherapy as salvage therapy for patients with refractory germ cell tumors. | Cancer | 66 | 2476-2481 | [http://dx.doi.org/10.1002/1097-0142(19901215)66:12<2476::aid-cncr2820661206>3.0.co;2-d](http://dx.doi.org/10.1002/1097-0142(19901215)66:12%3c2476::aid-cncr2820661206%3e3.0.co;2-d) |
| Motzer RJ, Cooper K, Geller NL, Pfister DG, Lin SY, Bajorin D, Scher HI, Herr H, Fair W and Morse M | 1990 | Carboplatin, etoposide, and bleomycin for patients with poor-risk germ cell tumors. | Cancer | 65 | 2465-2470 | [http://dx.doi.org/10.1002/1097-0142(19900601)65:11<2465::aid-cncr2820651112>3.0.co;2-7](http://dx.doi.org/10.1002/1097-0142(19900601)65:11%3c2465::aid-cncr2820651112%3e3.0.co;2-7) |
| Motzer RJ, Geller NL and Bosl GJ | 1990 | The effect of a 7-day delay in chemotherapy cycles on complete response and event-free survival in good-risk disseminated germ cell tumor patients. | Cancer | 66 | 857-861 | [http://dx.doi.org/10.1002/1097-0142(19900901)66:5<857::aid-cncr2820660508>3.0.co;2-g](http://dx.doi.org/10.1002/1097-0142(19900901)66:5%3c857::aid-cncr2820660508%3e3.0.co;2-g) |
| Nichols CR, Saxman S, Williams SD, Loehrer PJ, Miller ME, Wright C and Einhorn LH | 1990 | Primary mediastinal nonseminomatous germ cell tumors. A modern single institution experience. | Cancer | 65 | 1641-1646 | [http://dx.doi.org/10.1002/1097-0142(19900401)65:7<1641::aid-cncr2820650731>3.0.co;2-u](http://dx.doi.org/10.1002/1097-0142(19900401)65:7%3c1641::aid-cncr2820650731%3e3.0.co;2-u) |
| Ortin TT, Shostak CA and Donaldson SS | 1990 | Gonadal status and reproductive function following treatment for Hodgkin's Disease in childhood: the Stanford experience. | International Journal of Radiation Oncology, Biology, Physics | 19 | 873-880 |  |
| Ozkaynak MF, Ortega JA, Laug W, Gilsanz V and Isaacs HJ | 1990 | Role of chemotherapy in pediatric pulmonary blastoma. | Medical and Pediatric Oncology | 18 | 53-56 | <http://dx.doi.org/10.1002/mpo.2950180111> |
| Pinkerton CR, Broadbent V, Horwich A, Levitt J, McElwain TJ, Meller ST, Mott M, Oakhill A and Pritchard J | 1990 | 'JEB'--a carboplatin based regimen for malignant germ cell tumours in children. | British Journal of Cancer | 62 | 257-262 | <http://dx.doi.org/10.1038/bjc.1990.272> |
| Siimes MA and Rautonen J | 1990 | Small testicles with impaired production of sperm in adult male survivors of childhood malignancies. | Cancer | 65 | 1303-1306 | [http://dx.doi.org/10.1002/1097-0142(19900315)65:6<1303::aid-cncr2820650608>3.0.co;2-d](http://dx.doi.org/10.1002/1097-0142(19900315)65:6%3c1303::aid-cncr2820650608%3e3.0.co;2-d) |
| Wright CD, Kesler KA, Nichols CR, Mahomed Y, Einhorn LH, Miller ME and Brown JW | 1990 | Primary mediastinal nonseminomatous germ cell tumors. Results of a multimodality approach. | The Journal of Thoracic and Cardiovascular Surgery | 99 | 210-217 |  |
| Ablin AR, Krailo MD, Ramsay NK, Malogolowkin MH, Isaacs H, Raney RB, Adkins J, Hays DM, Benjamin DR and Grosfeld JL | 1991 | Results of treatment of malignant germ cell tumors in 93 children: a report from the Childrens Cancer Study Group. | Journal of Clinical Oncology : official Journal of the American Society of Clinical Oncology | 9 | 1782-1792 | <http://dx.doi.org/10.1200/JCO.1991.9.10.1782> |
|  | 1991 | Treatment of good-risk disseminated non-seminomatous germ cell tumours: the less bleomycin, the more cisplatin? | European Journal of Cancer (Oxford, England : 1990) | 27 | 1715 | <http://dx.doi.org/10.1016/0277-5379(91)90458-p> |
| Douek E, Kingston JE, Malpas JS and Plowman PN | 1991 | Platinum-based chemotherapy for recurrent CNS tumours in young patients. | Journal of neUrology, NeuroSurgery, and psychiatry | 54 | 722-725 | <http://dx.doi.org/10.1136/jnnp.54.8.722> |
| Harstrick A, Schmoll HJ, Wilke H, Kohne-Wompner CH, Stahl M, Schober C, Casper J, Bruderek L, Schmoll E and Bokemeyer C | 1991 | Cisplatin, etoposide, and ifosfamide salvage therapy for refractory or relapsing germ cell carcinoma. | Journal of Clinical Oncology : official Journal of the American Society of Clinical Oncology | 9 | 1549-1555 | <http://dx.doi.org/10.1200/JCO.1991.9.9.1549> |
| Kushner BH, Hajdu SI, Gulati SC, Erlandson RA, Exelby PR and Lieberman PH | 1991 | Extracranial primitive neuroectodermal tumors. The Memorial Sloan- Kettering Cancer Center experience. | Cancer | 67 | 1825-1829 | [http://dx.doi.org/10.1002/1097-0142(19910401)67:7<1825::aid-cncr2820670702>3.0.co;2-3](http://dx.doi.org/10.1002/1097-0142(19910401)67:7%3c1825::aid-cncr2820670702%3e3.0.co;2-3) |
| Lewis CR, Fossa SD, Mead G, ten Bokkel Huinink W, Harding MJ, Mill L, Paul J, Jones WG, Rodenburg CJ and Cantwell B | 1991 | BOP/VIP--a new platinum-intensive chemotherapy regimen for poor prognosis germ cell tumours. | Annals of Oncology : official Journal of the European Society for Medical Oncology | 2 | 203-211 | <http://dx.doi.org/10.1093/oxfordjournals.annonc.a057906> |
| Longo DL, Glatstein E, Duffey PL, Young RC, Hubbard SM, Urba WJ, Wesley MN, Raubitschek A, Jaffe ES and Wiernik PH | 1991 | Radiation therapy versus combination chemotherapy in the treatment of early-stage Hodgkin's Disease: seven-year results of a prospective randomized trial. | Journal of Clinical Oncology : official Journal of the American Society of Clinical Oncology | 9 | 906-917 | <http://dx.doi.org/10.1200/JCO.1991.9.6.906> |
| Motzer RJ, Geller NL, Tan CC, Herr H, Morse M, Fair W, Sheinfeld J, Sogani P, Russo P and Bosl GJ | 1991 | Salvage chemotherapy for patients with germ cell tumors. The Memorial Sloan-Kettering Cancer Center experience (1979-1989). | Cancer | 67 | 1305-1310 | [http://dx.doi.org/10.1002/1097-0142(19910301)67:5<1305::aid-cncr2820670506>3.0.co;2-j](http://dx.doi.org/10.1002/1097-0142(19910301)67:5%3c1305::aid-cncr2820670506%3e3.0.co;2-j) |
| Packer RJ, Sutton LN, Goldwein JW, Perilongo G, Bunin G, Ryan J, Cohen BH, D'Angio G, Kramer ED and Zimmerman RA | 1991 | Improved survival with the use of adjuvant chemotherapy in the treatment of medulloblastoma. | Journal of NeuroSurgery | 74 | 433-440 | <http://dx.doi.org/10.3171/jns.1991.74.3.0433> |
| Sanders JE | 1991 | Endocrine problems in children after bone marrow transplant for hematologic malignancies. The Long-term Follow-up Team. | Bone Marrow Transplantation | 8  Suppl 1 | 2-Apr |  |
| Santini G, Coser P, Chisesi T, Porcellini A, Sertoli R, Contu A, Vinante O, Congiu AM, Carella AM and D'Amico T | 1991 | Autologous bone marrow transplantation for advanced stage adult lymphoblastic lymphoma in first complete remission. Report of the Non-Hodgkin's Lymphoma Cooperative Study Group (NHLCSG). | Annals of Oncology : official Journal of the European Society for Medical Oncology | 2 Suppl 2 | 181-185 | <http://dx.doi.org/10.1007/978-1-4899-7305-4_29> |
| Tjulandin SA, Khlebnov AV, Nasirova RJ, Mikhina ZP, Molchanov GV, Sholokhov VN, Sokolov VA, Vetrova NA and Garin AM | 1991 | VAB-6 and cisplatin-cyclophosphamide combinations in the treatment of metastatic seminoma patients: the U.S.S.R. experience. | Annals of Oncology : official Journal of the European Society for Medical Oncology | 2 | 667-672 | <http://dx.doi.org/10.1093/oxfordjournals.annonc.a058046> |
| von Eschenbach AC, Swanson DA, Johnson DE, Wishnow KI, Stephenson RA and Babaian RJ | 1991 | Preservation of retroperitoneal lymph nodes in patients with locoregional nonseminomatous germ cell tumors: surveillance and primary chemotherapy. | Progress in Clinical and Biological Research | 370 | 385-392 |  |
| Wollner N, Ghavimi F, Wachtel A, Luks E, Exelby P and Woodruff J | 1991 | Germ cell tumors in children: gonadal and extragonadal. | Medical and Pediatric Oncology | 19 | 228-239 | <http://dx.doi.org/10.1002/mpo.2950190405> |
| Barrett AJ | 1992 | Allogeneic bone marrow transplantation for acute lymphoblastic leukaemia. | Leukemia | 6  Suppl 2 | 139-143 |  |
| Broun ER, Nichols CR, Kneebone P, Williams SD, Loehrer PJ, Einhorn LH and Tricot GJ | 1992 | Long-term outcome of patients with relapsed and refractory germ cell tumors treated with high-dose chemotherapy and autologous bone marrow rescue. | Annals of Internal Medicine | 117 | 124-128 | <http://dx.doi.org/10.7326/0003-4819-117-2-124> |
| Carl J, Christensen TB and von der Maase H | 1992 | Cisplatinum dose dependent response in germ cell cancer evaluated by tumour marker modelling. | Acta Oncologica (Stockholm, Sweden) | 31 | 749-753 | <http://dx.doi.org/10.3109/02841869209083865> |
| Droz JP, Pico JL, Ghosn M, Kramar A, Rey A, Ostronoff M and Baume D | 1992 | A phase II trial of early intensive chemotherapy with autologous bone marrow transplantation in the treatment of poor prognosis non seminomatous germ cell tumors. | Bulletin du Cancer | 79 | 497-507 |  |
| Hansen SW | 1992 | Late-effects after treatment for germ-cell cancer with cisplatin, vinblastine, and bleomycin. | Danish Medical Bulletin | 39 | 391-399 |  |
| Horwich A, Dearnaley DP, A'Hern R, Mason M, Thomas G, Jay G and Nicholls J | 1992 | The activity of single-agent carboplatin in advanced seminoma. | European Journal of Cancer (Oxford, England : 1990) | 28A | 1307-1310 | <http://dx.doi.org/10.1016/0959-8049(92)90505-v> |
| Husband DJ and Green JA | 1992 | POMB/ACE chemotherapy in non-seminomatous germ cell tumours: outcome and importance of dose intensity. | European Journal of Cancer (Oxford, England : 1990) | 28 | 86-91 | <http://dx.doi.org/10.1016/0959-8049(92)90392-f> |
| Marina N, Fontanesi J, Kun L, Rao B, Jenkins JJ, Thompson EI and Etcubanas E | 1992 | Treatment of childhood germ cell tumors. Review of the St. Jude experience from 1979 to 1988. | Cancer | 70 | 2568-2575 | [http://dx.doi.org/10.1002/1097-0142(19921115)70:10<2568::aid-cncr2820701028>3.0.co;2-1](http://dx.doi.org/10.1002/1097-0142(19921115)70:10%3c2568::aid-cncr2820701028%3e3.0.co;2-1) |
| Mead GM, Stenning SP, Parkinson MC, Horwich A, Fossa SD, Wilkinson PM, Kaye SB, Newlands ES and Cook PA | 1992 | The Second Medical Research Council study of prognostic factors in nonseminomatous germ cell tumors. Medical Research Council Testicular Tumour Working Party. | Journal of Clinical Oncology : official Journal of the American Society of Clinical Oncology | 10 | 85-94 | <http://dx.doi.org/10.1200/JCO.1992.10.1.85> |
| O'Brien ME, Pinkerton CR, Kingston J, Mott M, Tait D, Meller S, Radford M, Malpas J and McElwain TJ | 1992 | 'VEEP' in children with Hodgkin's Disease--a regimen to decrease late sequelae. | British Journal of Cancer | 65 | 756-760 | <http://dx.doi.org/10.1038/bjc.1992.159> |
| Ondrus D, Hornak M, Matoska J, Kausitz J and Belan V | 1992 | Primary chemotherapy in the management of low stage (IIA and IIB) non- seminomatous germ cell testicular tumours. | International Urology and Nephrology | 24 | 299-304 | <http://dx.doi.org/10.1007/bf02549539> |
| Patel SR, Buckner JC, Smithson WA, Scheithauer BW and Groover RV | 1992 | Cisplatin-based chemotherapy in primary central nervous system germ cell tumors. | Journal of neuro-Oncology | 12 | 47-52 | <http://dx.doi.org/10.1007/bf00172456> |
| Pizzocaro G, Salvioni R, Piva L, Faustini M, Nicolai N and Gianni L | 1992 | Modified cisplatin, etoposide (or vinblastine) and ifosfamide salvage therapy for male germ-cell tumors. Long-term results. | Annals of Oncology : official Journal of the European Society for Medical Oncology | 3 | 211-216 | <http://dx.doi.org/10.1093/oxfordjournals.annonc.a058154> |
| Saikia T, Hedge U, Advani S, Ramakrishnan G, Kulkarni J and Desai PB | 1992 | Cisplatin-based (PVB+M) chemotherapy in good-risk and poor-risk nonseminomatous germ cell tumors. | Journal of Surgical Oncology | 51 | 100-103 | <http://dx.doi.org/10.1002/jso.2930510207> |
| Schellong G, Bramswig JH and Hornig-Franz I | 1992 | Treatment of children with Hodgkin's Disease--results of the German Pediatric Oncology Group. | Annals of Oncology : official Journal of the European Society for Medical Oncology | 3 Suppl 4 | 73-76 | <http://dx.doi.org/10.1093/annonc/3.suppl_4.s73> |
| Sebag-Montefiore DJ, Douek E, Kingston JE and Plowman PN | 1992 | Intracranial germ cell tumours: I. Experience with platinum based chemotherapy and implications for curative chemoradiotherapy. | Clinical Oncology (Royal College of Radiologists (Great Britain)) | 4 | 345-350 | <http://dx.doi.org/10.1016/s0936-6555(05)81122-7> |
| Bajorin DF, Sarosdy MF, Pfister DG, Mazumdar M, Motzer RJ, Scher HI, Geller NL, Fair WR, Herr H and Sogani P | 1993 | Randomized trial of etoposide and cisplatin versus etoposide and carboplatin in patients with good-risk germ cell tumors: a multiinstitutional study. | Journal of Clinical Oncology : official Journal of the American Society of Clinical Oncology | 11 | 598-606 | <http://dx.doi.org/10.1200/JCO.1993.11.4.598> |
| Barnett MJ, Coppin CM, Murray N, Nevill TJ, Reece DE, Klingemann HG, Shepherd JD, Nantel SH, Sutherland HJ and Phillips GL | 1993 | High-dose chemotherapy and autologous bone marrow transplantation for patients with poor prognosis nonseminomatous germ cell tumours. | British Journal of Cancer | 68 | 594-598 | <http://dx.doi.org/10.1038/bjc.1993.392> |
| Bokemeyer C, Schmoll HJ, Harstrick A, Illiger HJ, Metzner B, Rath U, Hohnloser J, Clemm C, Berdel W and Siegert W | 1993 | A phase I/II study of a stepwise dose-escalated regimen of cisplatin, etoposide and ifosfamide plus granulocyte-macrophage colony-stimulating factor (GM-CSF) in patients with advanced germ cell tumours. | European Journal of Cancer (Oxford, England : 1990) | 29A | 2225-2231 | <http://dx.doi.org/10.1016/0959-8049(93)90211-w> |
| Chevreau C, Droz JP, Pico JL, Biron P, Kerbrat P, Cure H, Heron JF, Chevallier B, Fargeot P and Kramar A | 1993 | Early intensified chemotherapy with autologous bone marrow transplantation in first line treatment of poor risk non-seminomatous germ cell tumours. Preliminary results of a French randomized trial. | European Urology | 23 | 213-7; discussion 218 | <http://dx.doi.org/10.1159/000474596> |
| Childs WJ, Goldstraw P, Nicholls JE, Dearnaley DP and Horwich A | 1993 | Primary malignant mediastinal germ cell tumours: improved prognosis with platinum-based chemotherapy and surgery. | British Journal of Cancer | 67 | 1098-1101 | <http://dx.doi.org/10.1038/bjc.1993.201> |
| De Sanctis V, Galimberti M, Lucarelli G, Angelucci E, Ughi M, Baronciani D, Polchi P, Giardini C, Bagni B and Vullo C | 1993 | Pubertal development in thalassaemic patients after allogenic bone marrow transplantation. | European Journal of Pediatrics | 152 | 993-997 |  |
| Duffner PK, Horowitz ME, Krischer JP, Friedman HS, Burger PC, Cohen ME, Sanford RA, Mulhern RK, James HE and Freeman CR | 1993 | Postoperative chemotherapy and delayed radiation in children less than three years of age with malignant brain tumors. | The New England Journal of Medicine | 328 | 1725-1731 | <http://dx.doi.org/10.1056/NEJM199306173282401> |
| Dulmet EM, Macchiarini P, Suc B and Verley JM | 1993 | Germ cell tumors of the mediastinum. A 30-year experience. | Cancer | 72 | 1894-1901 | [http://dx.doi.org/10.1002/1097-0142(19930915)72:6<1894::aid-cncr2820720617>3.0.co;2-6](http://dx.doi.org/10.1002/1097-0142(19930915)72:6%3c1894::aid-cncr2820720617%3e3.0.co;2-6) |
| Goldwein JW, Radcliffe J, Packer RJ, Sutton LN, Lange B, Rorke LB and D'Angio GJ | 1993 | Results of a pilot study of low-dose craniospinal radiation therapy plus chemotherapy for children younger than 5 years with primitive neuroectodermal tumors. | Cancer | 71 | 2647-2652 | [http://dx.doi.org/10.1002/1097-0142(19930415)71:8<2647::aid-cncr2820710833>3.0.co;2-s](http://dx.doi.org/10.1002/1097-0142(19930415)71:8%3c2647::aid-cncr2820710833%3e3.0.co;2-s) |
| Halperin EC, Friedman HS, Schold SCJ, Fuchs HE, Oakes WJ, Hockenberger B and Burger PC | 1993 | Surgery, hyperfractionated craniospinal irradiation, and adjuvant chemotherapy in the management of supratentorial embryonal neuroepithelial neoplasms in children. | Surgical Neurology | 40 | 278-283 | <http://dx.doi.org/10.1016/0090-3019(93)90138-q> |
| Josefsen D, Ous S, Hoie J, Stenwig AE and Fossa SD | 1993 | Salvage treatment in male patients with germ cell tumours. | British Journal of Cancer | 67 | 568-572 | <http://dx.doi.org/10.1038/bjc.1993.104> |
| Kattan J, Mahjoubi M, Droz JP, Kramar A, Culine S, Boutan-Laroze A and Chazard M | 1993 | High failure rate of carboplatin-etoposide combination in good risk non- seminomatous germ cell tumours. | European Journal of Cancer (Oxford, England : 1990) | 29A | 1504-1509 | <http://dx.doi.org/10.1016/0959-8049(93)90283-l> |
| Motzer RJ, Gulati SC, Tong WP, Menendez-Botet C, Lyn P, Mazumdar M, Vlamis V, Lin S and Bosl GJ | 1993 | Phase I trial with pharmacokinetic analyses of high-dose carboplatin, etoposide, and cyclophosphamide with autologous bone marrow transplantation in patients with refractory germ cell tumors. | Cancer Research | 53 | 3730-3735 |  |
| Muller U and Stahel RA | 1993 | Gonadal function after MACOP-B or VACOP-B with or without dose intensification and ABMT in young patients with aggressive non-Hodgkin's lymphoma. | Annals of Oncology : official Journal of the European Society for Medical Oncology | 4 | 399-402 | <http://dx.doi.org/10.1093/oxfordjournals.annonc.a058519> |
| Pico JL, Ibrahim A, Castagna L, Bourhis JH, Chazard M, Maraninchi D and Droz JP | 1993 | Escalating high-dose carboplatin and autologous bone marrow transplantation in solid tumors. | Oncology | 50 Suppl 2 | 47-52 | <http://dx.doi.org/10.1159/000227261> |
| Shafford EA, Kingston JE, Malpas JS, Plowman PN, Pritchard J, Savage MO and Eden OB | 1993 | Testicular function following the treatment of Hodgkin's Disease in childhood. | British Journal of Cancer | 68 | 1199-1204 | <http://dx.doi.org/10.1038/bjc.1993.504> |
| Siimes MA, Lie SO, Andersen O, Marky I, Rautonen J and Hertz H | 1993 | Prophylactic cranial irradiation increases the risk of testicular damage in adult males surviving ALL in childhood. | Medical and Pediatric Oncology | 21 | 117-121 |  |
| Studer UE, Fey MF, Calderoni A, Kraft R, Mazzucchelli L and Sonntag RW | 1993 | Adjuvant chemotherapy after orchiectomy in high-risk patients with clinical stage I non-seminomatous testicular cancer. | European Urology | 23 | 444-449 | <http://dx.doi.org/10.1159/000474650> |
| Tjulandin SA, Garin AM, Mescheryakov AA, Perevodchikova NI, Gorbunova VA, Sokolov AV, Ljubimova NV, Mironova GT, Molchanov GV and Ozols RF | 1993 | Cisplatin-etoposide and carboplatin-etoposide induction chemotherapy for good-risk patients with germ cell tumors. | Annals of Oncology : official Journal of the European Society for Medical Oncology | 4 | 663-667 | <http://dx.doi.org/10.1093/oxfordjournals.annonc.a058621> |
| Allen JC, DaRosso RC, Donahue B and Nirenberg A | 1994 | A phase II trial of preirradiation carboplatin in newly diagnosed germinoma of the central nervous system. | Cancer | 74 | 940-944 | [http://dx.doi.org/10.1002/1097-0142(19940801)74:3<940::aid-cncr2820740323>3.0.co;2-u](http://dx.doi.org/10.1002/1097-0142(19940801)74:3%3c940::aid-cncr2820740323%3e3.0.co;2-u) |
|  | 1994 | The use of carboplatin in malignant germ cell tumours. | European Journal of Cancer (Oxford, England : 1990) | 30A | 721-722 | <http://dx.doi.org/10.1016/0959-8049(94)90560-6> |
| Bosl GJ and Bajorin DF | 1994 | Etoposide plus carboplatin or cisplatin in good-risk patients with germ cell tumors: a randomized comparison. | Seminars in Oncology | 21 | 61-64 |  |
| Broun ER, Nichols CR, Turns M, Williams SD, Loehrer PJ, Roth BJ, Lazarus HM and Einhorn LH | 1994 | Early salvage therapy for germ cell cancer using high dose chemotherapy with autologous bone marrow support. | Cancer | 73 | 1716-1720 | [http://dx.doi.org/10.1002/1097-0142(19940315)73:6<1716::aid-cncr2820730627>3.0.co;2-l](http://dx.doi.org/10.1002/1097-0142(19940315)73:6%3c1716::aid-cncr2820730627%3e3.0.co;2-l) |
| Eastham JA, Wilson TG, Russell C, Ahlering TE and Skinner DG | 1994 | Surgical resection in patients with nonseminomatous germ cell tumor who fail to normalize serum tumor markers after chemotherapy. | Urology | 43 | 74-80 | <http://dx.doi.org/10.1016/s0090-4295(94)80269-6> |
| Goldman JM | 1994 | Management of chronic myeloid leukaemia. | Blood Reviews | 8 | 21-29 |  |
| Goss PE, Schwertfeger L, Blackstein ME, Iscoe NA, Ginsberg RJ, Simpson WJ, Jones DP and Shepherd FA | 1994 | Extragonadal germ cell tumors. A 14-year Toronto experience. | Cancer | 73 | 1971-1979 | [http://dx.doi.org/10.1002/1097-0142(19940401)73:7<1971::aid-cncr2820730731>3.0.co;2-a](http://dx.doi.org/10.1002/1097-0142(19940401)73:7%3c1971::aid-cncr2820730731%3e3.0.co;2-a) |
| Haas RJ, Schmidt P, Gobel U and Harms D | 1994 | Treatment of malignant testicular tumors in childhood: results of the German National Study 1982-1992. | Medical and Pediatric Oncology | 23 | 400-405 | <http://dx.doi.org/10.1002/mpo.2950230503> |
| Horwich A, Dearnaley DP, Norman A, Nicolls J and Hendry WF | 1994 | Accelerated chemotherapy for poor prognosis germ cell tumours. | European Journal of Cancer (Oxford, England : 1990) | 30A | 1607-1611 | <http://dx.doi.org/10.1016/0959-8049(94)00329-4> |
| Ledermann JA, Holden L, Newlands ES, Begent RH, Rustin GJ, Bagshawe KD and Brampton M | 1994 | The long-term outcome of patients who relapse after chemotherapy for non- seminomatous germ cell tumours. | British Journal of Urology | 74 | 225-230 | <http://dx.doi.org/10.1111/j.1464-410x.1994.tb16591.x> |
| Marina NM, Rodman JH, Murry DJ, Shema SJ, Bowman LC, Jones DP, Furman W, Meyer WH and Pratt CB | 1994 | Phase I study of escalating targeted doses of carboplatin combined with ifosfamide and etoposide in treatment of newly diagnosed pediatric solid tumors. | Journal of the National Cancer Institute | 86 | 544-548 | <http://dx.doi.org/10.1093/jnci/86.7.544> |
| Mencel PJ, Motzer RJ, Mazumdar M, Vlamis V, Bajorin DF and Bosl GJ | 1994 | Advanced seminoma: treatment results, survival, and prognostic factors in 142 patients. | Journal of Clinical Oncology : official Journal of the American Society of Clinical Oncology | 12 | 120-126 | <http://dx.doi.org/10.1200/JCO.1994.12.1.120> |
| Nair R, Pai SK, Saikia TK, Nair CN, Kurkure PA, Gopal R, Sampat MS and Advani SH | 1994 | Malignant germ cell tumors in childhood. | Journal of Surgical Oncology | 56 | 186-190 | <http://dx.doi.org/10.1002/jso.2930560313> |
| Petersen PM, Hansen SW, Giwercman A, Rorth M and Skakkebaek NE | 1994 | Dose-dependent impairment of testicular function in patients treated with cisplatin-based chemotherapy for germ cell cancer. | Annals of Oncology : official Journal of the European Society for Medical Oncology | 5 | 355-358 | <http://dx.doi.org/10.1093/oxfordjournals.annonc.a058840> |
| Radford JA, Clark S, Crowther D and Shalet SM | 1994 | Male fertility after VAPEC-B chemotherapy for Hodgkin's Disease and non-Hodgkin's lymphoma. | British Journal of Cancer | 69 | 379-381 | <http://dx.doi.org/10.1038/bjc.1994.69> |
| Schellong G, Bramswig JH, Hornig-Franz I, Schwarze EW, Potter R and Wannenmacher M | 1994 | Hodgkin's Disease in children: combined modality treatment for stages IA, IB, and IIA. Results in 356 patients of the German/Austrian Pediatric Study Group. | Annals of Oncology : official Journal of the European Society for Medical Oncology | 5 Suppl 2 | 113-115 | <http://dx.doi.org/10.1093/annonc/5.suppl_2.s113> |
| Sertoli MR, Santini G, Chisesi T, Congiu AM, Rubagotti A, Contu A, Salvagno L, Coser P, Porcellini A and Vespignani M | 1994 | MACOP-B versus ProMACE-MOPP in the treatment of advanced diffuse non-Hodgkin's lymphoma: results of a prospective randomized trial by the non-Hodgkin's Lymphoma Cooperative Study Group. | Journal of Clinical Oncology : official Journal of the American Society of Clinical Oncology | 12 | 1366-1374 | <http://dx.doi.org/10.1200/JCO.1994.12.7.1366> |
| Siegert W, Beyer J, Strohscheer I, Baurmann H, Oettle H, Zingsem J, Zimmermann R, Bokemeyer C, Schmoll HJ and Huhn D | 1994 | High-dose treatment with carboplatin, etoposide, and ifosfamide followed by autologous stem-cell transplantation in relapsed or refractory germ cell cancer: a phase I/II study. The German Testicular Cancer Cooperative Study Group. | Journal of Clinical Oncology : official Journal of the American Society of Clinical Oncology | 12 | 1223-1231 | <http://dx.doi.org/10.1200/JCO.1994.12.6.1223> |
| Yeung AW, Pang YK, Tsang YC and Wong SW | 1994 | Double-cycle high-dose chemotherapy with peripheral blood stem cells and hematopoietic growth factor support in patients with advanced solid tumor. A pilot study by the Hong Kong Biotherapy Group. | Cancer | 73 | 1960-1970 | [http://dx.doi.org/10.1002/1097-0142(19940401)73:7<1960::aid-cncr2820730730>3.0.co;2-h](http://dx.doi.org/10.1002/1097-0142(19940401)73:7%3c1960::aid-cncr2820730730%3e3.0.co;2-h) |
| Amato RJ, Ellerhorst J, Banks M and Logothetis CJ | 1995 | Carboplatin and ifosfamide and selective consolidation in advanced seminoma. | European Journal of Cancer (Oxford, England : 1990) | 31A | 2223-2228 | <http://dx.doi.org/10.1016/0959-8049(95)00341-x> |
| Aparicio J, Montalar J, Munarriz EB, Reynes G, Gomez-Codina J, Pastor M and Herranz C | 1995 | Extragonadal germ cell tumors: prognostic factors and long-term follow- up. | European Urology | 28 | 19-24 | <http://dx.doi.org/10.1159/000475014> |
| Chang TK, Wong TT and Hwang B | 1995 | Combination chemotherapy with vinblastine, bleomycin, cisplatin, and etoposide (VBPE) in children with primary intracranial germ cell tumors. | Medical and Pediatric Oncology | 24 | 368-372 | <http://dx.doi.org/10.1002/mpo.2950240606> |
| Fields KK, Elfenbein GJ, Lazarus HM, Cooper BW, Perkins JB, Creger RJ, Ballester OF, Hiemenz JH, Janssen WE and Zorsky PE | 1995 | Maximum-tolerated doses of ifosfamide, carboplatin, and etoposide given over 6 days followed by autologous stem-cell rescue: toxicity profile. | Journal of Clinical Oncology : official Journal of the American Society of Clinical Oncology | 13 | 323-332 | <http://dx.doi.org/10.1200/JCO.1995.13.2.323> |
| Fossa SD, Droz JP, Stoter G, Kaye SB, Vermeylen K and Sylvester R | 1995 | Cisplatin, vincristine and ifosphamide combination chemotherapy of metastatic seminoma: results of EORTC trial 30874. EORTC GU Group. | British Journal of Cancer | 71 | 619-624 | <http://dx.doi.org/10.1038/bjc.1995.121> |
| Gerl A, Clemm C, Schmeller N, Dienemann H, Lamerz R, Kriegmair M and Wilmanns W | 1995 | Outcome analysis after post-chemotherapy surgery in patients with non- seminomatous germ cell tumours. | Annals of Oncology : official Journal of the European Society for Medical Oncology | 6 | 483-488 | <http://dx.doi.org/10.1093/oxfordjournals.annonc.a059219> |
| Haas RJ and Schmidt P | 1995 | Testicular germ-cell tumors in childhood and adolescence. | World Journal of Urology | 13 | 203-208 |  |
| Haas RJ, Schmidt P, Gobel U and Harms D | 1995 | Testicular germ cell tumors. Results of the GPO MAHO studies -82, -88, -92. | Klinische Padiatrie | 207 | 145-150 | <http://dx.doi.org/10.1055/s-2008-1046531> |
| Heideman RL, Kovnar EH, Kellie SJ, Douglass EC, Gajjar AJ, Walter AW, Langston JA, Jenkins JJ, Li Y and Greenwald C | 1995 | Preirradiation chemotherapy with carboplatin and etoposide in newly diagnosed embryonal pediatric CNS tumors. | Journal of Clinical Oncology : official Journal of the American Society of Clinical Oncology | 13 | 2247-2254 | <http://dx.doi.org/10.1200/JCO.1995.13.9.2247> |
| Kapoor G, Advani SH, Nair CN, Pai K, Kurkure PA, Nair R, Saikia TK, Vege D and Desai PB | 1995 | Pediatric germ cell tumor. An experience with BEP. | Journal of Pediatric Hematology/Oncology | 17 | 318-324 | <http://dx.doi.org/10.1097/00043426-199511000-00007> |
| Loehrer PJS, Johnson D, Elson P, Einhorn LH and Trump D | 1995 | Importance of bleomycin in favorable-prognosis disseminated germ cell tumors: an Eastern Cooperative Oncology Group trial. | Journal of Clinical Oncology : official Journal of the American Society of Clinical Oncology | 13 | 470-476 | <http://dx.doi.org/10.1200/JCO.1995.13.2.470> |
| Lopes LF, de Camargo B, Dondonis M, de Araujo RA and Morinaka E | 1995 | Response to high-dose cisplatin and etoposide in advanced germ cell tumors in children: results of the Brazilian Germ Cell Tumor Study. | Medical and Pediatric Oncology | 25 | 396-399 | <http://dx.doi.org/10.1002/mpo.2950250506> |
| Molassiotis A, van den Akker OB, Milligan DW and Boughton BJ | 1995 | Gonadal function and psychosexual adjustment in male long-term survivors of bone marrow transplantation. | Bone Marrow Transplantation | 16 | 253-259 |  |
| Motzer RJ, Sheinfeld J, Mazumdar M, Bajorin DF, Bosl GJ, Herr H, Lyn P and Vlamis V | 1995 | Etoposide and cisplatin adjuvant therapy for patients with pathologic stage II germ cell tumors. | Journal of Clinical Oncology : official Journal of the American Society of Clinical Oncology | 13 | 2700-2704 | <http://dx.doi.org/10.1200/JCO.1995.13.11.2700> |
| Nademanee A, O'Donnell MR, Snyder DS, Schmidt GM, Parker PM, Stein AS, Smith EP, Molina A, Stepan DE and Somlo G | 1995 | High-dose chemotherapy with or without total body irradiation followed by autologous bone marrow and/or peripheral blood stem cell transplantation for patients with relapsed and refractory Hodgkin's Disease: results in 85 patients with analysis of prognos | Blood | 85 | 1381-1390 |  |
| Stephenson WT, Poirier SM, Rubin L and Einhorn LH | 1995 | Evaluation of reproductive capacity in germ cell tumor patients following treatment with cisplatin, etoposide, and bleomycin. | Journal of Clinical Oncology : official Journal of the American Society of Clinical Oncology | 13 | 2278-2280 | <http://dx.doi.org/10.1200/JCO.1995.13.9.2278> |
| Touroutoglou N, Dimopoulos MA, Younes A, Hess M, Pugh W, Cox J, Cabanillas F and Sarris AH | 1995 | Testicular lymphoma: late relapses and poor outcome despite doxorubicin- based therapy. | Journal of Clinical Oncology : official Journal of the American Society of Clinical Oncology | 13 | 1361-1367 | <http://dx.doi.org/10.1200/JCO.1995.13.6.1361> |
| van Hoff J, Grier HE, Douglass EC and Green DM | 1995 | Etoposide, ifosfamide, and cisplatin therapy for refractory childhood solid tumors. Response and toxicity. | Cancer | 75 | 2966-2970 | [http://dx.doi.org/10.1002/1097-0142(19950615)75:12<2966::aid-cncr2820751226>3.0.co;2-w](http://dx.doi.org/10.1002/1097-0142(19950615)75:12%3c2966::aid-cncr2820751226%3e3.0.co;2-w) |
| Williams PC, Henner WD, Roman-Goldstein S, Dahlborg SA, Brummett RE, Tableman M, Dana BW and Neuwelt EA | 1995 | Toxicity and efficacy of carboplatin and etoposide in conjunction with disruption of the blood-brain tumor barrier in the treatment of intracranial neoplasms. | NeuroSurgery | 37 | 17-18 | <http://dx.doi.org/10.1227/00006123-199507000-00003> |
| Balmaceda C, Heller G, Rosenblum M, Diez B, Villablanca JG, Kellie S, Maher P, Vlamis V, Walker RW, Leibel S and Finlay JL | 1996 | Chemotherapy without irradiation--a novel approach for newly diagnosed CNS germ cell tumors: results of an international cooperative trial. The First International Central Nervous System Germ Cell Tumor Study. | Journal of Clinical Oncology : official Journal of the American Society of Clinical Oncology | 14 | 2908-2915 | <http://dx.doi.org/10.1200/JCO.1996.14.11.2908> |
| Benisovich VI, Silverman L, Slifkin R, Stone N and Cohen E | 1996 | Cisplatin-based chemotherapy in renal transplant recipients. A case report and a review of the literature. | Cancer | 77 | 160-163 | [http://dx.doi.org/10.1002/(SICI)1097-0142(19960101)77:1<160::AID-CNCR26>3.0.CO;2-2](http://dx.doi.org/10.1002/(SICI)1097-0142(19960101)77:1%3c160::AID-CNCR26%3e3.0.CO;2-2) |
| Blanke C, Loehrer PJ, Nichols CR and Einhorn LH | 1996 | A phase II trial of VP-16, ifosfamide, cisplatin, vinblastine, and bleomycin in advanced germ-cell tumors. | American Journal of Clinical Oncology | 19 | 487-491 | <http://dx.doi.org/10.1097/00000421-199610000-00012> |
| Bokemeyer C, Hartmann JT, Kuczyk MA, Truss MC, Beyer J, Jonas U and Kanz L | 1996 | The role of paclitaxel in chemosensitive urological malignancies: current strategies in bladder cancer and testicular germ-cell tumors. | World Journal of Urology | 14 | 354-359 |  |
| Dieckmann KP, Krain J, Kuster J and Bruggeboes B | 1996 | Adjuvant carboplatin treatment for seminoma clinical stage I. | Journal of Cancer Research and Clinical Oncology | 122 | 63-66 | <http://dx.doi.org/10.1007/bf01203075> |
| Farhat F, Culine S, Theodore C, Bekradda M, Terrier-Lacombe MJ and Droz JP | 1996 | Cisplatin and ifosfamide with either vinblastine or etoposide as salvage therapy for refractory or relapsing germ cell tumor patients: the Institut Gustave Roussy experience. | Cancer | 77 | 1193-1197 | [http://dx.doi.org/10.1002/(sici)1097-0142(19960315)77:6<1193::aid-cncr28>3.0.co;2-w](http://dx.doi.org/10.1002/(sici)1097-0142(19960315)77:6%3c1193::aid-cncr28%3e3.0.co;2-w) |
| Gerl A, Clemm C, Lamerz R and Wilmanns W | 1996 | Cisplatin-based chemotherapy of primary extragonadal germ cell tumors. A single institution experience. | Cancer | 77 | 526-532 | [http://dx.doi.org/10.1002/(SICI)1097-0142(19960201)77:3<526::AID-CNCR15>3.0.CO;2-6](http://dx.doi.org/10.1002/(SICI)1097-0142(19960201)77:3%3c526::AID-CNCR15%3e3.0.CO;2-6) |
| Gerl A, Clemm C, Schmeller N, Hartenstein R, Lamerz R and Wilmanns W | 1996 | Advances in the management of metastatic non-seminomatous germ cell tumours during the cisplatin era: a single-institution experience. | British Journal of Cancer | 74 | 1280-1285 | <http://dx.doi.org/10.1038/bjc.1996.530> |
| Halperin EC | 1996 | Long-term results of therapy for stage C neuroblastoma. | Journal of Surgical Oncology | 63 | 172-178 | [http://dx.doi.org/10.1002/(SICI)1096-9098(199611)63:3<172::AID-JSO7>3.0.CO;2-A](http://dx.doi.org/10.1002/(SICI)1096-9098(199611)63:3%3c172::AID-JSO7%3e3.0.CO;2-A) |
|  | 1996 | Carboplatin in the combination chemotherapy of non-seminomatous germ cell tumours. | Annals of Oncology : official Journal of the European Society for Medical Oncology | 7 | 989-991 | <http://dx.doi.org/10.1093/oxfordjournals.annonc.a010521> |
| Kobayashi H, Urashima M, Hoshi Y, Uchiyama H, Fujisawa K, Akatsuka J, Maekawa K and Hurusato M | 1996 | Testicular morphological changes in children with acute lymphoblastic leukemia following chemotherapy. | Acta paediatrica Japonica : Overseas edition | 38 | 640-643 |  |
| Mackie EJ, Radford M and Shalet SM | 1996 | Gonadal function following chemotherapy for childhood Hodgkin's Disease. | Medical and Pediatric Oncology | 27 | 74-78 | [http://dx.doi.org/10.1002/(SICI)1096-911X(199608)27:2<74::AID-MPO2>3.0.CO;2-Q](http://dx.doi.org/10.1002/(SICI)1096-911X(199608)27:2%3c74::AID-MPO2%3e3.0.CO;2-Q) |
| Margolin K, Doroshow JH, Ahn C, Hamasaki V, Leong L, Morgan R, Raschko J, Shibata S, Somlo G and Tetef M | 1996 | Treatment of germ cell cancer with two cycles of high-dose ifosfamide, carboplatin, and etoposide with autologous stem-cell support. | Journal of Clinical Oncology : official Journal of the American Society of Clinical Oncology | 14 | 2631-2637 | <http://dx.doi.org/10.1200/JCO.1996.14.10.2631> |
| Schellong G | 1996 | Treatment of children and adolescents with Hodgkin's Disease: the experience of the German-Austrian Paediatric Study Group. | Bailliere's Clinical Haematology | 9 | 619-634 |  |
| Schiffman KS, Bensinger WI, Appelbaum FR, Rowley S, Lilleby K, Clift RA, Weaver CH, Demirer T, Sanders JE, Petersdorf S, Gooley T, Weiden P, Zuckerman N, Montgomery P, Maziarz R, Klarnet JP, Rivkin S, Trueblood K, Storb R, Holmberg L and Buckner CD | 1996 | Phase II study of high-dose busulfan, melphalan and thiotepa with autologous peripheral blood stem cell support in patients with malignant Disease. | Bone Marrow Transplantation | 17 | 943-950 |  |
| Shanta V, Maitreyan V, Sagar TG, Gajalakshmi CK and Rajalekshmy KR | 1996 | Prognostic variables and survival in pediatric acute lymphoblastic leukemias: cancer institute experience. | Pediatric Hematology and Oncology | 13 | 205-216 |  |
| Stemmer SM, Cagnoni PJ, Shpall EJ, Bearman SI, Matthes S, Dufton C, Day T, Taffs S, Hami L, Martinez C, Purdy MH, Arron J and Jones RB | 1996 | High-dose paclitaxel, cyclophosphamide, and cisplatin with autologous hematopoietic progenitor-cell support: a phase I trial. | Journal of Clinical Oncology : official Journal of the American Society of Clinical Oncology | 14 | 1463-1472 | <http://dx.doi.org/10.1200/JCO.1996.14.5.1463> |
| Ben Arush MW, Roguin A, Zamir E, el-Hassid R, Pries D, Gaitini D, Dale A and Postovsky S | 1997 | Bleomycin and cyclophosphamide toxicity simulating metastatic nodules to the lungs in childhood cancer. | Pediatric Hematology and Oncology | 14 | 381-386 |  |
| Beyer J, Kingreen D, Krause M, Schleicher J, Schwaner I, Schwella N, Huhn D and Siegert W | 1997 | Long-term survival of patients with recurrent or refractory germ cell tumors after high dose chemotherapy. | Cancer | 79 | 161-168 |  |
| Bower M, Newlands ES, Holden L, Rustin GJ and Begent RH | 1997 | Treatment of men with metastatic non-seminomatous germ cell tumours with cyclical POMB/ACE chemotherapy. | Annals of Oncology : official Journal of the European Society for Medical Oncology | 8 | 477-483 | <http://dx.doi.org/10.1023/a:1008279222625> |
| Calaminus G, Andreussi L, Garre ML, Kortmann RD, Schober R and Gobel U | 1997 | Secreting germ cell tumors of the central nervous system (CNS). First results of the cooperative German/Italian pilot study (CNS sGCT). | Klinische Padiatrie | 209 | 222-227 | <http://dx.doi.org/10.1055/s-2008-1043954> |
| Chamberlain MC | 1997 | Pediatric leptomeningeal metastases: outcome following combined therapy. | Journal of Child Neurology | 12 | 53-59 | <http://dx.doi.org/10.1177/088307389701200109> |
| Coogan CL, Foster RS, Rowland RG, Bihrle R, Smith ERJ, Einhorn LH, Roth BJ and Donohue JP | 1997 | Postchemotherapy retroperitoneal lymph node dissection is effective therapy in selected patients with elevated tumor markers after primary chemotherapy alone. | Urology | 50 | 957-962 | <http://dx.doi.org/10.1016/S0090-4295(97)00458-5> |
| Culine S, Philippot I, Farhat F, Theodore C, Terrier-Lacombe MJ and Droz JP | 1997 | Evaluation of long-term results of a modified VAB-6 chemotherapy regimen in a cohort of good-risk metastatic non seminomatous germ-cell tumors. | Bulletin du Cancer | 84 | 368-372 |  |
| Culine S, Theodore C, Bekradda M, Farhat F, Terrier-Lacombe MJ and Droz JP | 1997 | Experience with bleomycin, etoposide, cisplatin (BEP) and alternating cisplatin, cyclophosphamide, doxorubicin (CISCA(II))/vinblastine, bleomycin (VB(IV)) regimens of chemotherapy in poor-risk nonseminomatous germ cell tumors. | American Journal of Clinical Oncology | 20 | 184-188 | <http://dx.doi.org/10.1097/00000421-199704000-00017> |
| Fossa SD, Oliver RT, Stenning SP, Horwich A, Wilkinson P, Read G, Mead GM, Roberts JT, Rustin G, Cullen MH, Kaye SB, Harland SJ and Cook P | 1997 | Prognostic factors for patients with advanced seminoma treated with platinum-based chemotherapy. | European Journal of Cancer (Oxford, England : 1990) | 33 | 1380-1387 | <http://dx.doi.org/10.1016/s0959-8049(96)00425-x> |
| Graham ML, Herndon JEn, Casey JR, Chaffee S, Ciocci GH, Krischer JP, Kurtzberg J, Laughlin MJ, Longee DC, Olson JF, Paleologus N, Pennington CN and Friedman HS | 1997 | High-dose chemotherapy with autologous stem-cell rescue in patients with recurrent and high-risk pediatric brain tumors. | Journal of Clinical Oncology : official Journal of the American Society of Clinical Oncology | 15 | 1814-1823 | <http://dx.doi.org/10.1200/JCO.1997.15.5.1814> |
| Hollender A, Stenwig EA, Ous S and Fossa SD | 1997 | Survival of patients with viable malignant non-seminomatous germ cell tumour persistent after cisplatin-based induction chemotherapy. | European Urology | 31 | 141-147 | <http://dx.doi.org/10.1159/000474439> |
| Horwich A, Sleijfer DT, Fossa SD, Kaye SB, Oliver RT, Cullen MH, Mead GM, de Wit R, de Mulder PH, Dearnaley DP, Cook PA, Sylvester RJ and Stenning SP | 1997 | Randomized trial of bleomycin, etoposide, and cisplatin compared with bleomycin, etoposide, and carboplatin in good-prognosis metastatic nonseminomatous germ cell cancer: a Multiinstitutional Medical Research Council/European Organization for Research and | Journal of Clinical Oncology : official Journal of the American Society of Clinical Oncology | 15 | 1844-1852 | <http://dx.doi.org/10.1200/JCO.1997.15.5.1844> |
| Krege S, Kalund G, Otto T, Goepel M and Rubben H | 1997 | Phase II study: adjuvant single-agent carboplatin therapy for clinical stage I seminoma. | European Urology | 31 | 405-407 | <http://dx.doi.org/10.1159/000474497> |
| Loehrer PJS, Chen M, Kim K, Aisner SC, Einhorn LH, Livingston R and Johnson D | 1997 | Cisplatin, doxorubicin, and cyclophosphamide plus thoracic radiation therapy for limited-stage unresectable thymoma: an intergroup trial. | Journal of Clinical Oncology : official Journal of the American Society of Clinical Oncology | 15 | 3093-3099 | <http://dx.doi.org/10.1200/JCO.1997.15.9.3093> |
| Longo DL, Glatstein E, Duffey PL, Young RC, Ihde DC, Bastian AW, Wilson WH, Wittes RE, Jaffe ES, Hubbard SM and DeVita VTJ | 1997 | Alternating MOPP and ABVD chemotherapy plus mantle-field radiation therapy in patients with massive mediastinal Hodgkin's Disease. | Journal of Clinical Oncology : official Journal of the American Society of Clinical Oncology | 15 | 3338-3346 | <http://dx.doi.org/10.1200/JCO.1997.15.11.3338> |
| Matsutani M, Sano K, Takakura K, Fujimaki T, Nakamura O, Funata N and Seto T | 1997 | Primary intracranial germ cell tumors: a clinical analysis of 153 histologically verified cases. | Journal of NeuroSurgery | 86 | 446-455 | <http://dx.doi.org/10.3171/jns.1997.86.3.0446> |
| McCaffrey JA, Mazumdar M, Bajorin DF, Bosl GJ, Vlamis V and Motzer RJ | 1997 | Ifosfamide- and cisplatin-containing chemotherapy as first-line salvage therapy in germ cell tumors: response and survival. | Journal of Clinical Oncology : official Journal of the American Society of Clinical Oncology | 15 | 2559-2563 | <http://dx.doi.org/10.1200/JCO.1997.15.7.2559> |
| Michel G, Socie G, Gebhard F, Bernaudin F, Thuret I, Vannier JP, Demeocq F, Leverger G, Pico JL, Rubie H, Mechinaud F, Reiffers J, Gratecos N, Troussard X, Jouet JP, Simonin G, Gluckman E and Maraninchi D | 1997 | Late effects of allogeneic bone marrow transplantation for children with acute myeloblastic leukemia in first complete remission: the impact of conditioning regimen without total-body irradiation--a report from the Societe Francaise de Greffe de Moelle. | Journal of Clinical Oncology : official Journal of the American Society of Clinical Oncology | 15 | 2238-2246 | <http://dx.doi.org/10.1200/JCO.1997.15.6.2238> |
| Motzer RJ, Mazumdar M, Bajorin DF, Bosl GJ, Lyn P and Vlamis V | 1997 | High-dose carboplatin, etoposide, and cyclophosphamide with autologous bone marrow transplantation in first-line therapy for patients with poor- risk germ cell tumors. | Journal of Clinical Oncology : official Journal of the American Society of Clinical Oncology | 15 | 2546-2552 | <http://dx.doi.org/10.1200/JCO.1997.15.7.2546> |
| Plowman PN, Kingston JE, Sebag-Montefiore D and Doughty D | 1997 | Clinical efficacy of perceived 'CNS friendly' chemoradiotherapy for primary intracranial germ cell tumours. | Clinical Oncology (Royal College of Radiologists (Great Britain)) | 9 | 48-53 | <http://dx.doi.org/10.1016/s0936-6555(97)80062-3> |
| Pont J, Bokemeyer C, Harstrick A, Sellner F, Greinix H and Stoiber F | 1997 | Chemotherapy for germ cell tumors relapsing after high-dose chemotherapy and stem cell support: a retrospective multicenter study of the Austrian Study Group on Urologic Oncology. | Annals of Oncology : official Journal of the European Society for Medical Oncology | 8 | 1229-1234 | <http://dx.doi.org/10.1023/a:1008286305312> |
| Robertson PL, DaRosso RC and Allen JC | 1997 | Improved prognosis of intracranial non-germinoma germ cell tumors with multimodality therapy. | Journal of Neuro-Oncology | 32 | 71-80 | <http://dx.doi.org/10.1023/a:1005732105727> |
| Stein ME, Kuten A, Drumea K, Moshkowitz B, Nativ C, Munichor M and Haim N | 1997 | Cisplatin-based chemotherapy in advanced seminoma: experience of the Northern Israel Oncology Center: 1981-1994. | Journal of Surgical Oncology | 64 | 331-335 | [http://dx.doi.org/10.1002/(sici)1096-9098(199704)64:4<331::aid-jso15>3.0.co;2-5](http://dx.doi.org/10.1002/(sici)1096-9098(199704)64:4%3c331::aid-jso15%3e3.0.co;2-5) |
| van den Berg H, Stuve W and Behrendt H | 1997 | Treatment of Hodgkin's Disease in children with alternating mechlorethamine, vincristine, procarbazine, and prednisone (MOPP) and adriamycin, bleomycin, vinblastine, and dacarbazine (ABVD) courses without radiotherapy. | Medical and Pediatric Oncology | 29 | 23-27 | [http://dx.doi.org/10.1002/(sici)1096-911x(199707)29:1<23::aid-mpo4>3.0.co;2-u](http://dx.doi.org/10.1002/(sici)1096-911x(199707)29:1%3c23::aid-mpo4%3e3.0.co;2-u) |
| Wessalowski R, Blohm M, Calaminus G, Engert J, Harms D, Krause I, Kruck H, Gruttner HP, Pape H and Gobel U | 1997 | Treatment results in children and adolescents with loco-regional recurrences of abdominal germ cell tumors (GCTs): a pilot-study with PEI chemotherapy and regional deep hyperthermia (RHT) in comparison to a matched cohort. | Klinische Padiatrie | 209 | 250-256 | <http://dx.doi.org/10.1055/s-2008-1043958> |
| Baranzelli MC, Patte C, Bouffet E, Portas M, Mechinaud-Lacroix F, Sariban E, Roche H and Kalifa C | 1998 | An attempt to treat pediatric intracranial alphaFP and betaHCG secreting germ cell tumors with chemotherapy alone. SFOP experience with 18 cases. Societe Francaise d'Oncologie Pediatrique. | Journal of Neuro-Oncology | 37 | 229-239 | <http://dx.doi.org/10.1023/a:1005863601481> |
| Brennemann W, Stoffel-Wagner B, Wichers M, Helmers A, Albers P, Mezger J and Klingmuller D | 1998 | Pretreatment follicle-stimulating hormone: a prognostic serum marker of spermatogenesis status in patients treated for germ cell cancer. | The Journal of Urology | 159 | 1942-1946 | <http://dx.doi.org/10.1016/s0022-5347(01)63203-8> |
| Culine S, Abs L, Terrier-Lacombe MJ, Theodore C, Wibault P and Droz JP | 1998 | Cisplatin-based chemotherapy in advanced seminoma: the Institut Gustave Roussy experience. | European Journal of Cancer (Oxford, England : 1990) | 34 | 353-358 | <http://dx.doi.org/10.1016/s0959-8049(97)10070-3> |
| Dahlborg SA, Petrillo A, Crossen JR, Roman-Goldstein S, Doolittle ND, Fuller KH and Neuwelt EA | 1998 | The potential for complete and durable response in nonglial primary brain tumors in children and young adults with enhanced chemotherapy delivery. | The Cancer Journal from Scientific American | 4 | 110-124 |  |
| Dueland S, Stenwig AE, Heilo A, Hoie J, Ous S and Fossa SD | 1998 | Treatment and outcome of patients with extragonadal germ cell tumours-- the Norwegian Radium Hospital's experience 1979-94. | British Journal of Cancer | 77 | 329-335 | <http://dx.doi.org/10.1038/bjc.1998.51> |
| Fizazi K, Culine S, Droz JP, Kramar A, Theodore C, Ruffie P and Le Chevalier T | 1998 | Primary mediastinal nonseminomatous germ cell tumors: results of modern therapy including cisplatin-based chemotherapy. | Journal of Clinical Oncology : official Journal of the American Society of Clinical Oncology | 16 | 725-732 | <http://dx.doi.org/10.1200/JCO.1998.16.2.725> |
| Fossa SD, Kaye SB, Mead GM, Cullen M, de Wit R, Bodrogi I, van Groeningen CJ, De Mulder PH, Stenning S, Lallemand E, De Prijck L and Collette L | 1998 | Filgrastim during combination chemotherapy of patients with poor- prognosis metastatic germ cell malignancy. European Organization for Research and Treatment of Cancer, Genito-Urinary Group, and the Medical Research Council Testicular Cancer Working Party | Journal of Clinical Oncology : official Journal of the American Society of Clinical Oncology | 16 | 716-724 | <http://dx.doi.org/10.1200/JCO.1998.16.2.716> |
| Gobel U, Calaminus G, Engert J, Kaatsch P, Gadner H, Bokkerink JP, Hass RJ, Waag K, Blohm ME, Dippert S, Teske C and Harms D | 1998 | Teratomas in infancy and childhood. | Medical and Pediatric Oncology | 31 | Aug-15 | [http://dx.doi.org/10.1002/(sici)1096-911x(199807)31:1<8::aid-mpo2>3.0.co;2-h](http://dx.doi.org/10.1002/(sici)1096-911x(199807)31:1%3c8::aid-mpo2%3e3.0.co;2-h) |
| Li CK, Shing MM, Chik KW, Kwan WH, Lai DH, Leung TF and Yuen PM | 1998 | Isolated testicular relapse after bone marrow transplant with total body irradiation and testicular boost in acute lymphoblastic leukemia. | Bone Marrow Transplantation | 22 | 397-399 | <http://dx.doi.org/10.1038/sj.bmt.1701340> |
| Liu HC, Liang DC, Chen SH, Liu FL, Chang PY, Sheu JC and Wang NL | 1998 | The stage I yolk sac tumor of testis in children younger than 2 years, chemotherapy or not? | Pediatric Hematology and Oncology | 15 | 223-228 |  |
| Loehrer PJS, Gonin R, Nichols CR, Weathers T and Einhorn LH | 1998 | Vinblastine plus ifosfamide plus cisplatin as initial salvage therapy in recurrent germ cell tumor. | Journal of Clinical Oncology : official Journal of the American Society of Clinical Oncology | 16 | 2500-2504 | <http://dx.doi.org/10.1200/JCO.1998.16.7.2500> |
| Matsutani M, Sano K, Takakura K, Fujimaki T and Nakamura O | 1998 | Combined treatment with chemotherapy and radiation therapy for intracranial germ cell tumors. | Child's nervous system : ChNS : official Journal of the International Society for Pediatric NeuroSurgery | 14 | 59-62 |  |
| Rick O, Beyer J, Kingreen D, Schwella N, Krusch A, Schleicher J, Kirsch A, Huhn D and Siegert W | 1998 | High-dose chemotherapy in germ cell tumours: a large single centre experience. | European Journal of Cancer (Oxford, England : 1990) | 34 | 1883-1888 | <http://dx.doi.org/10.1016/s0959-8049(98)00272-x> |
| Tjulandin SA, Titov DA, Breder VV, Sidorova NJ, Popov AJ, Kupchan DZ and Garin AM | 1998 | Paclitaxel and cisplatin as salvage treatment in patients with non- seminomatous germ cell tumour who failed to achieve a complete remission on induction chemotherapy. | Clinical Oncology (Royal College of Radiologists (Great Britain)) | 10 | 297-300 | <http://dx.doi.org/10.1016/s0936-6555(98)80080-0> |
| Baranzelli MC, Kramar A, Bouffet E, Quintana E, Rubie H, Edan C and Patte C | 1999 | Prognostic factors in children with localized malignant nonseminomatous germ cell tumors. | Journal of Clinical Oncology : official Journal of the American Society of Clinical Oncology | 17 | 1212 | <http://dx.doi.org/10.1200/JCO.1999.17.4.1212> |
| Bokemeyer C, Gerl A, Schoffski P, Harstrick A, Niederle N, Beyer J, Casper J, Schmoll HJ and Kanz L | 1999 | Gemcitabine in patients with relapsed or cisplatin-refractory testicular cancer. | Journal of Clinical Oncology : official Journal of the American Society of Clinical Oncology | 17 | 512-516 | <http://dx.doi.org/10.1200/JCO.1999.17.2.512> |
| Chik K, Li C, Shing MM, Leung T and Yuen PM | 1999 | Intracranial germ cell tumors in children with and without Down syndrome. | Journal of Pediatric Hematology/Oncology | 21 | 149-151 | <http://dx.doi.org/10.1097/00043426-199903000-00012> |
| de Wit R, Louwerens M, de Mulder PH, Verweij J, Rodenhuis S and Schornagel J | 1999 | Management of intermediate-prognosis germ-cell cancer: results of a phase I/II study of Taxol-BEP. | International Journal of Cancer | 83 | 831-833 | [http://dx.doi.org/10.1002/(sici)1097-0215(19991210)83:6<831::aid-ijc24>3.0.co;2-o](http://dx.doi.org/10.1002/(sici)1097-0215(19991210)83:6%3c831::aid-ijc24%3e3.0.co;2-o) |
| Einhorn LH, Stender MJ and Williams SD | 1999 | Phase II trial of gemcitabine in refractory germ cell tumors. | Journal of Clinical Oncology : official Journal of the American Society of Clinical Oncology | 17 | 509-511 | <http://dx.doi.org/10.1200/JCO.1999.17.2.509> |
| Flechon A, Biron P and Droz JP | 1999 | High-dose chemotherapy with hematopoietic stem-cell support in germ-cell tumor patient treatment: the French experience. | International Journal of Cancer | 83 | 844-847 | [http://dx.doi.org/10.1002/(sici)1097-0215(19991210)83:6<844::aid-ijc28>3.0.co;2-6](http://dx.doi.org/10.1002/(sici)1097-0215(19991210)83:6%3c844::aid-ijc28%3e3.0.co;2-6) |
| Fossa SD, Bokemeyer C, Gerl A, Culine S, Jones WG, Mead GM, Germa-Luch JR, Pont J, Schmoll HJ and Tjulandin S | 1999 | Treatment outcome of patients with brain metastases from malignant germ cell tumors. | Cancer | 85 | 988-997 | [http://dx.doi.org/10.1002/(sici)1097-0142(19990215)85:4<988::aid-cncr29>3.0.co;2-r](http://dx.doi.org/10.1002/(sici)1097-0142(19990215)85:4%3c988::aid-cncr29%3e3.0.co;2-r) |
| Fossa SD, Stenning SP, Gerl A, Horwich A, Clark PI, Wilkinson PM, Jones WG, Williams MV, Oliver RT, Newlands ES, Mead GM, Cullen MH, Kaye SB, Rustin GJ and Cook PA | 1999 | Prognostic factors in patients progressing after cisplatin-based chemotherapy for malignant non-seminomatous germ cell tumours. | British Journal of Cancer | 80 | 1392-1399 | <http://dx.doi.org/10.1038/sj.bjc.6690534> |
| Germa-Lluch JR, Garcia del Muro X, Tabernero JM, Sanchez M, Aparicio J, Alba E and Barnadas A | 1999 | BOMP/EPI intensive alternating chemotherapy for IGCCC poor-prognosis germ-cell tumors: the Spanish Germ-Cell Cancer Group experience (GG) | Annals of Oncology : official Journal of the European Society for Medical Oncology | 10 | 289-293 | <http://dx.doi.org/10.1023/a:1008351022211> |
| Gutierrez-Delgado F, Titov DA, Tjulandin SA and Garin AM | 1999 | Drug dose delivery and treatment outcome relationship in standard bleomycin, etoposide and cisplatin combination chemotherapy in nonseminomatous germ cell tumor patients. | Neoplasma | 46 | 190-195 |  |
| Haas RJ, Schmidt P, Gobel U and Harms D | 1999 | Testicular germ cell tumors, an update. Results of the German cooperative studies 1982-1997. | Klinische Padiatrie | 211 | 300-304 | <http://dx.doi.org/10.1055/s-2008-1043804> |
| Hara I, Yamada Y, Miyake H, Nomi M, Hara S, Yamanaka K, Takechi Y, Oka Y, Nakamura I, Gotoh A, Gohji K, Arakawa S and Kamidono S | 1999 | Clinical outcome of high-dose chemotherapy combined with peripheral blood stem cell transplantation for male germ cell tumors. | Anti-Cancer Drugs | 10 | 711-718 | <http://dx.doi.org/10.1097/00001813-199909000-00004> |
| Kasseyet S, Astoul P and Boutin C | 1999 | Results of a phase II trial of combined chemotherapy for patients with diffuse malignant mesothelioma of the pleura. | Cancer | 85 | 1740-1749 |  |
| Mahalati K, Bilen CY, Ozen H, Aki FT and Kendi S | 1999 | The management of brain metastasis in nonseminomatous germ cell tumours. | BJU International | 83 | 457-461 | <http://dx.doi.org/10.1046/j.1464-410x.1999.00967.x> |
| Marina NM, Cushing B, Giller R, Cohen L, Lauer SJ, Ablin A, Weetman R, Cullen J, Rogers P, Vinocur C, Stolar C, Rescorla F, Hawkins E, Heifetz S, Rao PV, Krailo M and Castleberry RP | 1999 | Complete surgical excision is effective treatment for children with immature teratomas with or without malignant elements: A Pediatric Oncology Group/Children's Cancer Group Intergroup Study. | Journal of Clinical Oncology : official Journal of the American Society of Clinical Oncology | 17 | 2137-2143 | <http://dx.doi.org/10.1200/JCO.1999.17.7.2137> |
| Morris MJ and Bosl GJ | 1999 | High-dose chemotherapy as primary treatment for poor-risk germ-cell tumors: the Memorial Sloan-Kettering experience (1988-1999). | International Journal of Cancer | 83 | 834-838 | [http://dx.doi.org/10.1002/(sici)1097-0215(19991210)83:6<834::aid-ijc25>3.0.co;2-i](http://dx.doi.org/10.1002/(sici)1097-0215(19991210)83:6%3c834::aid-ijc25%3e3.0.co;2-i) |
| Nam DH, Cho BK, Shin HJ, Ahn HS, Kim IH and Wang KC | 1999 | Treatment of intracranial nongerminomatous malignant germ cell tumor in children: the role of each treatment modality. | Child's nervous system : ChNS : official Journal of the International Society for Pediatric NeuroSurgery | 15 | 185-191 | <http://dx.doi.org/10.1007/s003810050366> |
| Pectasides D, Aravantinos G, Visvikis A, Bakoyiannis C, Halikia A, Kalofonos C, Kosmidis P, Skarlos D and Fountzilas G | 1999 | Platinum-based chemotherapy of primary extragonadal germ cell tumours: the Hellenic Cooperative Oncology Group experience. | Oncology | 57 | 1-Sep | <http://dx.doi.org/10.1159/000011993> |
| Rodenhuis S, de Wit R, de Mulder PH, Keizer HJ, Sleijfer DT, Lalisang RI, Bakker PJ, Mandjes I, Kooi M and de Vries EG | 1999 | A multi-center prospective phase II study of high-dose chemotherapy in germ-cell cancer patients relapsing from complete remission. | Annals of Oncology : official Journal of the European Society for Medical Oncology | 10 | 1467-1473 | <http://dx.doi.org/10.1023/a:1008328012040> |
| Schellong G, Potter R, Bramswig J, Wagner W, Prott FJ, Dorffel W, Korholz D, Mann G, Rath B, Reiter A, Weissbach G, Riepenhausen M, Thiemann M and Schwarze EW | 1999 | High cure rates and reduced long-term toxicity in pediatric Hodgkin's Disease: the German-Austrian multicenter trial DAL-HD-90. The German-Austrian Pediatric Hodgkin's Disease Study Group. | Journal of Clinical Oncology : official Journal of the American Society of Clinical Oncology | 17 | 3736-3744 | <http://dx.doi.org/10.1200/JCO.1999.17.12.3736> |
| Soulie P, Garrino C, Bensmaine MA, Bekradda M, Brain E, Di Palma M, Goupil A, Misset JL and Cvitkovic E | 1999 | Antitumoral activity of oxaliplatin/cisplatin-based combination therapy in cisplatin-refractory germ cell cancer patients. | Journal of Cancer Research and Clinical Oncology | 125 | 707-711 | <http://dx.doi.org/10.1007/s004320050338> |
| Tada T, Takizawa T, Nakazato F, Kobayashi S, Koike K, Oguchi M, Ishii E and Amano Y | 1999 | Treatment of intracranial nongerminomatous germ-cell tumor by high-dose chemotherapy and autologous stem-cell rescue. | Journal of Neuro-Oncology | 44 | 71-76 | <http://dx.doi.org/10.1023/a:1006395719917> |
| Ushio Y, Kochi M, Kuratsu J, Itoyama Y and Marubayashi T | 1999 | Preliminary observations for a new treatment in children with primary intracranial yolk sac tumor or embryonal carcinoma. Report of five cases. | Journal of NeuroSurgery | 90 | 133-137 | <http://dx.doi.org/10.3171/jns.1999.90.1.0133> |
| Amato RJ, Millikan R, Daliani D, Wood L, Logothetis C and Pollack A | 2000 | Cyclophosphamide and carboplatin and selective consolidation in advanced seminoma. | Clinical Cancer Research : an official Journal of the American Association for Cancer Research | 6 | 72-77 |  |
| Cicognani A, Cacciari E, Pasini A, Burnelli R, De Iasio R, Pirazzoli P and Paolucci G | 2000 | Low serum inhibin B levels as a marker of testicular damage after treatment for a childhood malignancy. | European Journal of Pediatrics | 159 | 103-107 |  |
| de Bono JS, Paul J, Simpson A, Anthoney A, Kirk D, Underwood M, Graham J and Kaye SB | 2000 | Improving the outcome of salvage treatment for non-seminomatous germ cell tumours (NSGCT). | British Journal of Cancer | 83 | 426-430 | <http://dx.doi.org/10.1054/bjoc.2000.1290> |
| Dieckmann KP, Bruggeboes B, Pichlmeier U, Kuster J, Mullerleile U and Bartels H | 2000 | Adjuvant treatment of clinical stage I seminoma: is a single course of carboplatin sufficient? | Urology | 55 | 102-106 | <http://dx.doi.org/10.1016/s0090-4295(99)00376-3> |
| Ganjoo KN, Rieger KM, Kesler KA, Sharma M, Heilman DK and Einhorn LH | 2000 | Results of modern therapy for patients with mediastinal nonseminomatous germ cell tumors. | Cancer | 88 | 1051-1056 | [http://dx.doi.org/10.1002/(sici)1097-0142(20000301)88:5<1051::aid-cncr15>3.0.co;2-r](http://dx.doi.org/10.1002/(sici)1097-0142(20000301)88:5%3c1051::aid-cncr15%3e3.0.co;2-r) |
| Kollmannsberger C, Nichols C, Bamberg M, Hartmann JT, Schleucher N, Beyer J, Schofski P, Derigs G, Ruther U, Bohlke I, Schmoll HJ, Kanz L and Bokemeyer C | 2000 | First-line high-dose chemotherapy +/- radiation therapy in patients with metastatic germ-cell cancer and brain metastases. | Annals of Oncology : official Journal of the European Society for Medical Oncology | 11 | 553-559 | <http://dx.doi.org/10.1023/a:1008388328809> |
| Kusumakumary P, Mathew BS, Hariharan S, Priyakumari T and Rajan B | 2000 | Testicular germ cell tumors in prepubertal children. | Pediatric Hematology and Oncology | 17 | 105-111 | <http://dx.doi.org/10.1080/088800100276721> |
| Mann JR, Raafat F, Robinson K, Imeson J, Gornall P, Sokal M, Gray E, McKeever P, Hale J, Bailey S and Oakhill A | 2000 | The United Kingdom Children's Cancer Study Group's second germ cell tumor study: carboplatin, etoposide, and bleomycin are effective treatment for children with malignant extracranial germ cell tumors, with acceptable toxicity. | Journal of Clinical Oncology : official Journal of the American Society of Clinical Oncology | 18 | 3809-3818 | <http://dx.doi.org/10.1200/JCO.2000.18.22.3809> |
| Mardiak J, Fuchsberger P, Lakota J, Salek T, Sycova-Mila Z, Drahokoupilova M, Balaz M and Koza I | 2000 | Sequential intermediate high-dose therapy with etoposide, ifosfamide and cisplatin for patients with germ cell tumors. | Neoplasma | 47 | 239-243 |  |
| Mardiak J, Salek T, Sycova-Mila Z, Sufliarsky J, Balaz M and Koza I | 2000 | Carboplatin and cyclophosphamide in the treatment of metastatic seminoma. | Neoplasma | 47 | 244-247 |  |
| Porcu P, Bhatia S, Sharma M and Einhorn LH | 2000 | Results of treatment after relapse from high-dose chemotherapy in germ cell tumors. | Journal of Clinical Oncology : official Journal of the American Society of Clinical Oncology | 18 | 1181-1186 | <http://dx.doi.org/10.1200/JCO.2000.18.6.1181> |
| Schneider DT, Calaminus G, Reinhard H, Gutjahr P, Kremens B, Harms D and Gobel U | 2000 | Primary mediastinal germ cell tumors in children and adolescents: results of the German cooperative protocols MAKEI 83/86, 89, and 96. | Journal of Clinical Oncology : official Journal of the American Society of Clinical Oncology | 18 | 832-839 | <http://dx.doi.org/10.1200/JCO.2000.18.4.832> |
| Shamash J, O'Doherty CA, Oliver RT, Kelsey S, Gupta RK, Gallagher CJ, Newland AC and Lister TA | 2000 | Should high-dose chemotherapy be used to consolidate second or third line treatment in relapsing germ cell tumours? | Acta oncologica (Stockholm, Sweden) | 39 | 857-863 | <http://dx.doi.org/10.1080/028418600750063622> |
| Sklar CA and LaQuaglia MP | 2000 | The long-term complications of chemotherapy in childhood genitourinary tumors. | The Urologic Clinics of North America | 27 | 563-8, x |  |
| Studer UE, Burkhard FC and Sonntag RW | 2000 | Risk adapted management with adjuvant chemotherapy in patients with high risk clinical stage i nonseminomatous germ cell tumor. | The Journal of Urology | 163 | 1785-1787 |  |
| Vaidya SJ, Atra A, Bahl S, Pinkerton CR, Calvagna V, Horton C, Milan S, Shepherd V, Brain C, Treleaven J, Powles R, Tait D and Meller ST | 2000 | Autologous bone marrow transplantation for childhood acute lymphoblastic leukaemia in second remission - long-term follow-up. | Bone Marrow Transplantation | 25 | 599-603 | <http://dx.doi.org/10.1038/sj.bmt.1702214> |
| Weissbach L, Bussar-Maatz R, Flechtner H, Pichlmeier U, Hartmann M and Keller L | 2000 | RPLND or primary chemotherapy in clinical stage IIA/B nonseminomatous germ cell tumors? Results of a prospective multicenter trial including quality of life assessment. | European Urology | 37 | 582-594 | <http://dx.doi.org/10.1159/000020197> |
|  | 2001 | ESMO minimum clinical recommendations for diagnosis, treatment and follow-up of mixed or non-seminomatous germ cell tumours (NSGCT). | Annals of Oncology : official Journal of the European Society for Medical Oncology | 12 | 1215-1216 |  |
| Arranz Arija JA, Garcia del Muro X, Guma J, Aparicio J, Salazar R, Saenz A, Carles J, Sanchez M and Germa-Lluch JR | 2001 | E400P in advanced seminoma of good prognosis according to the international germ cell cancer collaborative group (IGCCCG) classification: the Spanish Germ Cell Cancer Group experience. | Annals of Oncology : official Journal of the European Society for Medical Oncology | 12 | 487-491 | <http://dx.doi.org/10.1023/a:1011127715764> |
| Aviles A and Neri N | 2001 | Hematological malignancies and pregnancy: a final report of 84 children who received chemotherapy in utero. | Clinical Lymphoma | 2 | 173-177 |  |
| Billmire D, Vinocur C, Rescorla F, Colombani P, Cushing B, Hawkins E, London WB, Giller R and Lauer S | 2001 | Malignant mediastinal germ cell tumors: an intergroup study. | Journal of Pediatric Surgery | 36 | 18-24 | <http://dx.doi.org/10.1053/jpsu.2001.19995> |
| Bohlen D, Burkhard FC, Mills R, Sonntag RW and Studer UE | 2001 | Fertility and sexual function following orchiectomy and 2 cycles of chemotherapy for stage I high risk nonseminomatous germ cell cancer. | The Journal of Urology | 165 | 441-444 | <http://dx.doi.org/10.1097/00005392-200102000-00022> |
| de Wit R, Roberts JT, Wilkinson PM, de Mulder PH, Mead GM, Fossa SD, Cook P, de Prijck L, Stenning S and Collette L | 2001 | Equivalence of three or four cycles of bleomycin, etoposide, and cisplatin chemotherapy and of a 3- or 5-day schedule in good-prognosis germ cell cancer: a randomized study of the European Organization for Research and Treatment of Cancer Genitourinary Tr | Journal of Clinical Oncology : official Journal of the American Society of Clinical Oncology | 19 | 1629-1640 | <http://dx.doi.org/10.1200/JCO.2001.19.6.1629> |
| Fizazi K, Tjulandin S, Salvioni R, Germa-Lluch JR, Bouzy J, Ragan D, Bokemeyer C, Gerl A, Flechon A, de Bono JS, Stenning S, Horwich A, Pont J, Albers P, De Giorgi U, Bower M, Bulanov A, Pizzocaro G, Aparicio J, Nichols CR, Theodore C, Hartmann JT, Schmoll HJ, Kaye SB, Culine S, Droz JP and Mahe C | 2001 | Viable malignant cells after primary chemotherapy for disseminated nonseminomatous germ cell tumors: prognostic factors and role of postsurgery chemotherapy--results from an international study group. | Journal of Clinical Oncology : official Journal of the American Society of Clinical Oncology | 19 | 2647-2657 | <http://dx.doi.org/10.1200/JCO.2001.19.10.2647> |
| Flechon A, Rivoire M, Biron P and Droz JP | 2001 | Importance of surgery as salvage treatment after high dose chemotherapy failure in germ cell tumors. | The Journal of Urology | 165 | 1920-1926 | <http://dx.doi.org/10.1097/00005392-200106000-00019> |
| Ganjoo KN, Foster RS, Michael H, Donohue JP and Einhorn LH | 2001 | Germ cell tumor associated primitive neuroectodermal tumors. | The Journal of Urology | 165 | 1514-1516 |  |
| Gerl A, Muhlbayer D, Hansmann G, Mraz W and Hiddemann W | 2001 | The impact of chemotherapy on Leydig cell function in long term survivors of germ cell tumors. | Cancer | 91 | 1297-1303 | [http://dx.doi.org/10.1002/1097-0142(20010401)91:7<1297::aid-cncr1132>3.0.co;2-z](http://dx.doi.org/10.1002/1097-0142(20010401)91:7%3c1297::aid-cncr1132%3e3.0.co;2-z) |
| Gobel U, Schneider DT, Calaminus G, Jurgens H, Spaar HJ, Sternschulte W, Waag K and Harms D | 2001 | Multimodal treatment of malignant sacrococcygeal germ cell tumors: a prospective analysis of 66 patients of the German cooperative protocols MAKEI 83/86 and 89. | Journal of Clinical Oncology : official Journal of the American Society of Clinical Oncology | 19 | 1943-1950 | <http://dx.doi.org/10.1200/JCO.2001.19.7.1943> |
| Hartmann JT, Einhorn L, Nichols CR, Droz JP, Horwich A, Gerl A, Fossa SD, Beyer J, Pont J, Schmoll HJ, Kanz L and Bokemeyer C | 2001 | Second-line chemotherapy in patients with relapsed extragonadal nonseminomatous germ cell tumors: results of an international multicenter analysis. | Journal of Clinical Oncology : official Journal of the American Society of Clinical Oncology | 19 | 1641-1648 | <http://dx.doi.org/10.1200/JCO.2001.19.6.1641> |
| Rescorla F, Billmire D, Stolar C, Vinocur C, Colombani P, Cullen J, Giller R, Cushing B, Lauer S, Davis M, Hawkins E, Shuster J and Krailo M | 2001 | The effect of cisplatin dose and surgical resection in children with malignant germ cell tumors at the sacrococcygeal region: a pediatric intergroup trial (POG 9049/CCG 8882). | Journal of Pediatric Surgery | 36 | Dec-17 | <http://dx.doi.org/10.1053/jpsu.2001.19993> |
| Rick O, Bokemeyer C, Beyer J, Hartmann JT, Schwella N, Kingreen D, Neureither S, Metzner B, Casper J, Wandt H, Hartmann F, Schmoll HJ, Derigs G, Gerl A, Berdel WE, Kanz L and Siegert W | 2001 | Salvage treatment with paclitaxel, ifosfamide, and cisplatin plus high- dose carboplatin, etoposide, and thiotepa followed by autologous stem- cell rescue in patients with relapsed or refractory germ cell cancer. | Journal of Clinical Oncology : official Journal of the American Society of Clinical Oncology | 19 | 81-88 | <http://dx.doi.org/10.1200/JCO.2001.19.1.81> |
| Sonneveld DJ, Hoekstra HJ, van der Graaf WT, Sluiter WJ, Mulder NH, Willemse PH, Koops HS and Sleijfer DT | 2001 | Improved long term survival of patients with metastatic nonseminomatous testicular germ cell carcinoma in relation to prognostic classification systems during the cisplatin era. | Cancer | 91 | 1304-1315 |  |
| Bhutani M, Kumar L, Seth A, Thulkar S, Vijayaraghavan M and Kochupillai V | 2002 | Germ cell tumours of the testis: clinical features, treatment outcome and prognostic factors. | The National Medical Journal of India | 15 | 18-21 |  |
| Bokemeyer C, Nichols CR, Droz J-P, Schmoll H-J, Horwich A, Gerl A, Fossa SD, Beyer J, Pont J, Kanz L, Einhorn L and Hartmann JT | 2002 | Extragonadal germ cell tumors of the mediastinum and retroperitoneum: results from an international analysis. | Journal of Clinical Oncology : official Journal of the American Society of Clinical Oncology | 20 | 1864-1873 | <http://dx.doi.org/10.1200/JCO.2002.07.062> |
| Fizazi K, Prow DM, Do K-A, Wang X, Finn L, Kim J, Daliani D, Papandreou CN, Tu S-M, Millikan RE, Pagliaro LC, Logothetis CJ and Amato RJ | 2002 | Alternating dose-dense chemotherapy in patients with high volume disseminated non-seminomatous germ cell tumours. | British Journal of Cancer | 86 | 1555-1560 | <http://dx.doi.org/10.1038/sj.bjc.6600272> |
| Hsu Y-J, Pai L, Chen Y-C, Ho C-L, Kao W-Y and Chao T-Y | 2002 | Extragonadal germ cell tumors in Taiwan: an analysis of treatment results of 59 patients. | Cancer | 95 | 766-774 | <http://dx.doi.org/10.1002/cncr.10738> |
| Kellie SJ, Wong CKF, Pozza LD, Waters KD, Lockwood L, Mauger DC and White L | 2002 | Activity of postoperative carboplatin, etoposide, and high-dose methotrexate in pediatric CNS embryonal tumors: results of a phase II study in newly diagnosed children. | Medical and Pediatric Oncology | 39 | 168-174 | <http://dx.doi.org/10.1002/mpo.10137> |
| Miki T, Mizutani Y, Nonomura N, Nomoto T, Nakao M, Saiki S, Kotake T and Okuyama A | 2002 | Irinotecan plus cisplatin has substantial antitumor effect as salvage chemotherapy against germ cell tumors. | Cancer | 95 | 1879-1885 | <http://dx.doi.org/10.1002/cncr.10918> |
| Spermon JR, Roeleveld TA, van der Poel HG, Hulsbergen-van de Kaa CA, Ten Bokkel Huinink WW, van de Vijver M, Witjes JA and Horenblas S | 2002 | Comparison of surveillance and retroperitoneal lymph node dissection in Stage I nonseminomatous germ cell tumors. | Urology | 59 | 923-929 | <http://dx.doi.org/10.1016/s0090-4295(02)01528-5> |
| Steiner H, Holtl L, Wirtenberger W, Berger AP, Bartsch G and Hobisch A | 2002 | Long-term experience with carboplatin monotherapy for clinical stage I seminoma: a retrospective single-center study. | Urology | 60 | 324-328 | <http://dx.doi.org/10.1016/s0090-4295(02)01708-9> |
| Stern JW and Bunin N | 2002 | Prospective study of carboplatin-based chemotherapy for pediatric germ cell tumors. | Medical and Pediatric Oncology | 39 | 163-167 | <http://dx.doi.org/10.1002/mpo.10134> |
| Strumberg D, Brugge S, Korn MW, Koeppen S, Ranft J, Scheiber G, Reiners C, Mockel C, Seeber S and Scheulen ME | 2002 | Evaluation of long-term toxicity in patients after cisplatin-based chemotherapy for non-seminomatous testicular cancer. | Annals of Oncology : official Journal of the European Society for Medical Oncology | 13 | 229-236 | <http://dx.doi.org/10.1093/annonc/mdf058> |
| Suita S, Shono K, Tajiri T, Takamatsu T, Mizote H, Nagasaki A, Inomata Y, Hara T, Okamura J, Miyazaki S, Kawakami K, Eguchi H and Tsuneyoshi M | 2002 | Malignant germ cell tumors: clinical characteristics, treatment, and outcome. A report from the study group for Pediatric Solid Malignant Tumors in the Kyushu Area, Japan. | Journal of Pediatric Surgery | 37 | 1703-1706 | <http://dx.doi.org/10.1053/jpsu.2002.36700> |
| Terenziani M, Piva L, Spreafico F, Salvioni R, Massimino M, Luksch R, Cefalo G, Casanova M, Ferrari A, Polastri D, Mazza E, Bellani FF and Nicolai N | 2002 | Clinical stage I nonseminomatous germ cell tumors of the testis in childhood and adolescence: an analysis of 31 cases. | Journal of Pediatric Hematology/Oncology | 24 | 454-458 |  |
| Billmire D, Vinocur C, Rescorla F, Colombani P, Cushing B, Hawkins E, Davis M, London WB, Lauer S and Giller R | 2003 | Malignant retroperitoneal and abdominal germ cell tumors: an intergroup study. | Journal of Pediatric Surgery | 38 | 315-318 | <http://dx.doi.org/10.1053/jpsu.2003.50100> |
| Bokemeyer C, Schleucher N, Metzner B, Thomas M, Rick O, Schmoll H-J, Kollmannsberger C, Boehlke I, Kanz L and Hartmann JT | 2003 | First-line sequential high-dose VIP chemotherapy with autologous transplantation for patients with primary mediastinal nonseminomatous germ cell tumours: a prospective trial. | British Journal of Cancer | 89 | 29-35 | <http://dx.doi.org/10.1038/sj.bjc.6600999> |
| Calaminus G, Schneider DT, Bokkerink JPM, Gadner H, Harms D, Willers R and Gobel U | 2003 | Prognostic value of tumor size, metastases, extension into bone, and increased tumor marker in children with malignant sacrococcygeal germ cell tumors: a prospective evaluation of 71 patients treated in the German cooperative protocols Maligne Keimzelltum | Journal of Clinical Oncology : official Journal of the American Society of Clinical Oncology | 21 | 781-786 | <http://dx.doi.org/10.1200/JCO.2003.03.125> |
| Christian JA, Huddart RA, Norman A, Mason M, Fossa S, Aass N, Nicholl EJ, Dearnaley DP and Horwich A | 2003 | Intensive induction chemotherapy with CBOP/BEP in patients with poor prognosis germ cell tumors. | Journal of Clinical Oncology : official Journal of the American Society of Clinical Oncology | 21 | 871-877 | <http://dx.doi.org/10.1200/JCO.2003.05.155> |
| Cicognani A, Pasini A, Pession A, Pirazzoli P, Burnelli R, Barbieri E, Mazzanti L and Cacciari E | 2003 | Gonadal function and pubertal development after treatment of a childhood malignancy. | Journal of Pediatric endocrinology & metabolism : JPEM | 16 Suppl 2 | 321-326 |  |
| Gholam D, Fizazi K, Terrier-Lacombe M-J, Jan P, Culine S and Theodore C | 2003 | Advanced seminoma--treatment results and prognostic factors for survival after first-line, cisplatin-based chemotherapy and for patients with recurrent Disease: a single-institution experience in 145 patients. | Cancer | 98 | 745-752 | <http://dx.doi.org/10.1002/cncr.11574> |
| Huddart SN, Mann JR, Robinson K, Raafat F, Imeson J, Gornall P, Sokal M, Gray E, McKeever P and Oakhill A | 2003 | Sacrococcygeal teratomas: the UK Children's Cancer Study Group's experience. I. Neonatal. | Pediatric Surgery International | 19 | 47-51 | <http://dx.doi.org/10.1007/s00383-002-0884-2> |
| Jahnukainen K and Soder O | 2003 | Testicular function after cancer treatment in childhood. | Endocrine Development | 5 | 124-135 |  |
| Kawai K, Miyazaki J, Tsukamoto S, Hinotsu S, Hattori K, Shimazui T and Akaza H | 2003 | Paclitaxel, ifosfamide and cisplatin regimen is feasible for Japanese patients with advanced germ cell cancer. | Japanese Journal of Clinical Oncology | 33 | 127-131 | <http://dx.doi.org/10.1093/jjco/hyg029> |
| Lo Curto M, Lumia F, Alaggio R, Cecchetto G, Almasio P, Indolfi P, Siracusa F, Bagnulo S, De Bernardi B, De Laurentis T, Di Cataldo A and Tamaro P | 2003 | Malignant germ cell tumors in childhood: results of the first Italian cooperative study "TCG 91". | Medical and Pediatric Oncology | 41 | 417-425 | <http://dx.doi.org/10.1002/mpo.10324> |
| Patel MI, Motzer RJ and Sheinfeld J | 2003 | Management of recurrence and follow-up strategies for patients with seminoma and selected high-risk groups. | The Urologic Clinics of North America | 30 | 803-817 |  |
| Rescorla F, Billmire D, Vinocur C, Colombani P, London W, Giller R, Cushing B, Lauer S, Cullen J, Davis M and Hawkins E | 2003 | The effect of neoadjuvant chemotherapy and surgery in children with malignant germ cell tumors of the genital region: a pediatric intergroup trial. | Journal of Pediatric Surgery | 38 | 910-912 | <http://dx.doi.org/10.1016/s0022-3468(03)00121-0> |
| Schmoll H-J, Kollmannsberger C, Metzner B, Hartmann JT, Schleucher N, Schoffski P, Schleicher J, Rick O, Beyer J, Hossfeld D, Kanz L, Berdel WE, Andreesen R and Bokemeyer C | 2003 | Long-term results of first-line sequential high-dose etoposide, ifosfamide, and cisplatin chemotherapy plus autologous stem cell support for patients with advanced metastatic germ cell cancer: an extended phase I/II study of the German Testicular Cancer S | Journal of Clinical Oncology : official Journal of the American Society of Clinical Oncology | 21 | 4083-4091 | <http://dx.doi.org/10.1200/JCO.2003.09.035> |
| Schrama JG, Holtkamp MJ, Baars JW, Schornagel JH and Rodenhuis S | 2003 | Toxicity of the high-dose chemotherapy CTC regimen (cyclophosphamide, thiotepa, carboplatin): the Netherlands Cancer Institute experience. | British Journal of Cancer | 88 | 1831-1838 | <http://dx.doi.org/10.1038/sj.bjc.6601001> |
| Stewart RJ, Martelli H, Oberlin O, Rey A, Bouvet N, Spicer RD, Godzinski J and Stevens MCG | 2003 | Treatment of children with nonmetastatic paratesticular rhabdomyosarcoma: results of the Malignant Mesenchymal Tumors studies (MMT 84 and MMT 89) of the International Society of Pediatric Oncology. | Journal of Clinical Oncology : official Journal of the American Society of Clinical Oncology | 21 | 793-798 | <http://dx.doi.org/10.1200/JCO.2003.06.040> |
| Takeda S-i, Miyoshi S, Ohta M, Minami M, Masaoka A and Matsuda H | 2003 | Primary germ cell tumors in the mediastinum: a 50-year experience at a single Japanese institution. | Cancer | 97 | 367-376 | <http://dx.doi.org/10.1002/cncr.11068> |
| Vaena DA, Abonour R and Einhorn LH | 2003 | Long-term survival after high-dose salvage chemotherapy for germ cell malignancies with adverse prognostic variables. | Journal of Clinical Oncology : official Journal of the American Society of Clinical Oncology | 21 | 4100-4104 | <http://dx.doi.org/10.1200/JCO.2003.06.067> |
| Wessalowski R, Schneider DT, Mils O, Hannen M, Calaminus G, Engelbrecht V, Pape H, Willers R, Engert J, Harms D and Gobel U | 2003 | An approach for cure: PEI-chemotherapy and regional deep hyperthermia in children and adolescents with unresectable malignant tumors. | Klinische Padiatrie | 215 | 303-309 | <http://dx.doi.org/10.1055/s-2003-45500> |
| Bhala N, Coleman JM, Radstone CR, Horsman JM, George J, Hancock BW, Hatton MQ and Coleman RE | 2004 | The management and survival of patients with advanced germ-cell tumours: improving outcome in intermediate and poor prognosis patients. | Clinical Oncology (Royal College of Radiologists (Great Britain)) | 16 | 40-47 |  |
| Bokemeyer C, Kollmannsberger C, Stenning S, Hartmann JT, Horwich A, Clemm C, Gerl A, Meisner C, Ruckerl C-P, Schmoll H-J, Kanz L and Oliver T | 2004 | Metastatic seminoma treated with either single agent carboplatin or cisplatin-based combination chemotherapy: a pooled analysis of two randomised trials. | British Journal of Cancer | 91 | 683-687 | <http://dx.doi.org/10.1038/sj.bjc.6602020> |
| Cushing B, Giller R, Cullen JW, Marina NM, Lauer SJ, Olson TA, Rogers PC, Colombani P, Rescorla F, Billmire DF, Vinocur CD, Hawkins EP, Davis MM, Perlman EJ, London WB and Castleberry RP | 2004 | Randomized comparison of combination chemotherapy with etoposide, bleomycin, and either high-dose or standard-dose cisplatin in children and adolescents with high-risk malignant germ cell tumors: a pediatric intergroup study--Pediatric Oncology Group 9049 | Journal of Clinical Oncology : official Journal of the American Society of Clinical Oncology | 22 | 2691-2700 | <http://dx.doi.org/10.1200/JCO.2004.08.015> |
| De Giorgi U, Rosti G, Papiani G, Aieta M, Fochessati F, Paoluzzi L, Valduga F and Marangolo M | 2004 | Weekly gemcitabine, paclitaxel, oxaliplatin combination chemotherapy in patients with Cisplatin-refractory germ cell tumor: preliminary experience. | American Journal of Clinical Oncology | 27 | 457-460 | <http://dx.doi.org/10.1097/01.coc.0000128727.40450.9e> |
| Kollmannsberger C, Beyer J, Liersch R, Schoeffski P, Metzner B, Hartmann JT, Rick O, Stengele K, Hohloch K, Spott C, Kanz L and Bokemeyer C | 2004 | Combination chemotherapy with gemcitabine plus oxaliplatin in patients with intensively pretreated or refractory germ cell cancer: a study of the German Testicular Cancer Study Group. | Journal of Clinical Oncology : official Journal of the American Society of Clinical Oncology | 22 | 108-114 | <http://dx.doi.org/10.1200/JCO.2004.06.068> |
| Kondagunta GV, Sheinfeld J, Mazumdar M, Mariani TV, Bajorin D, Bacik J, Bosl GJ and Motzer RJ | 2004 | Relapse-free and overall survival in patients with pathologic stage II nonseminomatous germ cell cancer treated with etoposide and cisplatin adjuvant chemotherapy. | Journal of Clinical Oncology : official Journal of the American Society of Clinical Oncology | 22 | 464-467 | <http://dx.doi.org/10.1200/JCO.2004.07.178> |
| Miyake H, Muramaki M, Eto H, Kamidono S and Hara I | 2004 | Health-related quality of life after chemotherapy for advanced germ cell tumors: a comparison of standard-dose and high-dose chemotherapy. | International Journal of Urology : official Journal of the Japanese Urological Association | 11 | 542-546 | <http://dx.doi.org/10.1111/j.1442-2042.2004.00839.x> |
| Muramaki M, Hara I, Miyake H, Yamada Y, Kawabata G and Kamidono S | 2004 | Advances in the management of non-seminomatous germ cell tumors during the cisplatin era: a single-institution experience. | International Journal of Urology : official Journal of the Japanese Urological Association | 11 | 768-773 | <http://dx.doi.org/10.1111/j.1442-2042.2004.00889.x> |
| Oliver RTD, Ong J, Shamash J, Ravi R, Nagund V, Harper P, Ostrowski MJ, Sizer B, Levay J, Robinson A, Neal DE and Williams M | 2004 | Long-term follow-up of Anglian Germ Cell Cancer Group surveillance versus patients with Stage 1 nonseminoma treated with adjuvant chemotherapy. | Urology | 63 | 556-561 | <http://dx.doi.org/10.1016/j.urology.2003.10.023> |
| Pectasides D, Pectasides M, Farmakis D, Aravantinos G, Nikolaou M, Koumpou M, Gaglia A, Kostopoulou V, Mylonakis N, Economopoulos T and Raptis SA | 2004 | Oxaliplatin and irinotecan plus granulocyte-colony stimulating factor as third-line treatment in relapsed or cisplatin-refractory germ-cell tumor patients: a phase II study. | European Urology | 46 | 216-221 | <http://dx.doi.org/10.1016/j.eururo.2004.03.001> |
| Pectasides D, Pectasides M, Farmakis D, Aravantinos G, Nikolaou M, Koumpou M, Gaglia A, Kostopoulou V, Mylonakis N and Skarlos D | 2004 | Gemcitabine and oxaliplatin (GEMOX) in patients with cisplatin-refractory germ cell tumors: a phase II study. | Annals of Oncology : official Journal of the European Society for Medical Oncology | 15 | 493-497 | <http://dx.doi.org/10.1093/annonc/mdh103> |
| Schmoll HJ, Souchon R, Krege S, Albers P, Beyer J, Kollmannsberger C, Fossa SD, Skakkebaek NE, de Wit R, Fizazi K, Droz JP, Pizzocaro G, Daugaard G, de Mulder PHM, Horwich A, Oliver T, Huddart R, Rosti G, Paz Ares L, Pont O, Hartmann JT, Aass N, Algaba F, Bamberg M, Bodrogi I, Bokemeyer C, Classen J, Clemm S, Culine S, de Wit M, Derigs HG, Dieckmann KP, Flasshove M, Garcia del Muro X, Gerl A, Germa-Lluch JR, Hartmann M, Heidenreich A, Hoeltl W, Joffe J, Jones W, Kaiser G, Klepp O, Kliesch S, Kisbenedek L, Koehrmann KU, Kuczyk M, Laguna MP, Leiva O, Loy V, Mason MD, Mead GM, Mueller RP, Nicolai N, Oosterhof GON, Pottek T, Rick O, Schmidberger H, Sedlmayer F, Siegert W, Studer U, Tjulandin S, von der Maase H, Walz P, Weinknecht S, Weissbach L, Winter E and Wittekind C | 2004 | European consensus on diagnosis and treatment of germ cell cancer: a report of the European Germ Cell Cancer Consensus Group (EGCCCG). | Annals of Oncology : official Journal of the European Society for Medical Oncology | 15 | 1377-1399 | <http://dx.doi.org/10.1093/annonc/mdh301> |
| van den Berg H, Furstner F, van den Bos C and Behrendt H | 2004 | Decreasing the number of MOPP courses reduces gonadal damage in survivors of childhood Hodgkin Disease. | Pediatric Blood & Cancer | 42 | 210-215 | <http://dx.doi.org/10.1002/pbc.10422> |
| Varuni Kondagunta G, Bacik J, Schwartz L, Sheinfeld J, Bajorin D, Vuky J, Marion S, Mazumdar M, Bosl GJ and Motzer RJ | 2004 | Phase II trial of temozolomide in patients with cisplatin-refractory germ cell tumors. | Investigational New Drugs | 22 | 177-179 | <http://dx.doi.org/10.1023/B:DRUG.0000011794.21608.59> |
| Zaletel LZ, Bratanic N and Jereb B | 2004 | Gonadal function in patients treated for leukemia in childhood. | Leukemia & Lymphoma | 45 | 1797-1802 | <http://dx.doi.org/10.1080/1042819042000219458> |
| Dearnaley DP, Fossa SD, Kaye SB, Cullen MH, Harland SJ, Sokal MPJ, Graham JD, Roberts JT, Mead GM, Williams MV, Cook PA and Stenning SP | 2005 | Adjuvant bleomycin, vincristine and cisplatin (BOP) for high-risk stage I non-seminomatous germ cell tumours: a prospective trial (MRC TE17). | British Journal of Cancer | 92 | 2107-2113 | <http://dx.doi.org/10.1038/sj.bjc.6602624> |
| Fossa SD, Paluchowska B, Horwich A, Kaiser G, de Mulder PHM, Koriakine O, van Oosterom AT, de Prijck L, Collette L and de Wit R | 2005 | Intensive induction chemotherapy with C-BOP/BEP for intermediate- and poor-risk metastatic germ cell tumours (EORTC trial 30948). | British Journal of Cancer | 93 | 1209-1214 | <http://dx.doi.org/10.1038/sj.bjc.6602830> |
| Geyer JR, Sposto R, Jennings M, Boyett JM, Axtell RA, Breiger D, Broxson E, Donahue B, Finlay JL, Goldwein JW, Heier LA, Johnson D, Mazewski C, Miller DC, Packer R, Puccetti D, Radcliffe J, Tao ML and Shiminski-Maher T | 2005 | Multiagent chemotherapy and deferred radiotherapy in infants with malignant brain tumors: a report from the Children's Cancer Group. | Journal of Clinical Oncology : official Journal of the American Society of Clinical Oncology | 23 | 7621-7631 | <http://dx.doi.org/10.1200/JCO.2005.09.095> |
| Hara I, Yamada Y, Kumano M, Furukawa J, Yamanaka K and Fujisawa M | 2005 | High dose chemotherapy including paclitaxel (T-ICE) combined with peripheral blood stem cell transplantation for male germ cell tumor. Preliminary report. | International Journal of Urology : official Journal of the Japanese Urological Association | 12 | 1074-1078 | <http://dx.doi.org/10.1111/j.1442-2042.2005.01235.x> |
| Kondagunta GV, Bacik J, Bajorin D, Dobrzynski D, Sheinfeld J, Motzer RJ and Bosl GJ | 2005 | Etoposide and cisplatin chemotherapy for metastatic good-risk germ cell tumors. | Journal of Clinical Oncology : official Journal of the American Society of Clinical Oncology | 23 | 9290-9294 | <http://dx.doi.org/10.1200/JCO.2005.03.6616> |
| Kondagunta GV, Bacik J, Donadio A, Bajorin D, Marion S, Sheinfeld J, Bosl GJ and Motzer RJ | 2005 | Combination of paclitaxel, ifosfamide, and cisplatin is an effective second-line therapy for patients with relapsed testicular germ cell tumors. | Journal of Clinical Oncology : official Journal of the American Society of Clinical Oncology | 23 | 6549-6555 | <http://dx.doi.org/10.1200/JCO.2005.19.638> |
|  | 2005 | Carboplatin for stage I seminoma and the sword of Damocles. | Journal of Clinical Oncology : official Journal of the American Society of Clinical Oncology | 23 | 8566-8569 | <http://dx.doi.org/10.1200/JCO.2005.03.0361> |
| Mardiak J, Salek T, Sycova-Mila Z, Obertova J, Hlavata Z, Mego M, Reckova M and Koza I | 2005 | Paclitaxel plus ifosfamide and cisplatin in second-line treatment of germ cell tumors: a phase II study. | Neoplasma | 52 | 497-501 |  |
| Mardiak J, Salek T, Sycova-Mila Z, Obertova J, Hlavata Z, Mego M, Reckova M and Koza I | 2005 | Gemcitabine plus cisplatine and paclitaxel (GCP) in second-line treatment of germ cell tumors (GCT): a phase II study. | Neoplasma | 52 | 243-247 |  |
| Maroto P, Garcia del Muro X, Aparicio J, Paz-Ares L, Arranz JA, Guma J, Terrassa J, Barnadas J, Dorta J and Germa-Lluch JR | 2005 | Multicentre risk-adapted management for stage I non-seminomatous germ cell tumours. | Annals of Oncology : official Journal of the European Society for Medical Oncology | 16 | 1915-1920 | <http://dx.doi.org/10.1093/annonc/mdi397> |
| Mead GM, Cullen MH, Huddart R, Harper P, Rustin GJS, Cook PA, Stenning SP and Mason M | 2005 | A phase II trial of TIP (paclitaxel, ifosfamide and cisplatin) given as second-line (post-BEP) salvage chemotherapy for patients with metastatic germ cell cancer: a medical research council trial. | British Journal of Cancer | 93 | 178-184 | <http://dx.doi.org/10.1038/sj.bjc.6602682> |
| Moser EC, Noordijk EM, Carde P, Tirelli U, Baars JW, Thomas J, Bron D, Meerwaldt JH, van Glabbeke M, Raemaekers JMM and Kluin-Nelemans HC | 2005 | Late non-neoplastic events in patients with aggressive non-Hodgkin's lymphoma in four randomized European Organisation for Research and Treatment of Cancer trials. | Clinical Lymphoma & Myeloma | 6 | 122-130 | <http://dx.doi.org/10.3816/CLM.2005.n.038> |
| Nieto Y, Shpall EJ, Bearman SI, McSweeney PA, Cagnoni PJ, Matthes S, Gustafson D, Long M, Baron AE and Jones RB | 2005 | Phase I and pharmacokinetic study of docetaxel combined with melphalan and carboplatin, with autologous hematopoietic progenitor cell support, in patients with advanced refractory malignancies. | Biology of Blood and Marrow Transplantation : Journal of the American Society for Blood and Marrow Transplantation | 11 | 297-306 | <http://dx.doi.org/10.1016/j.bbmt.2005.01.002> |
| Oliver RTD, Mason MD, Mead GM, von der Maase H, Rustin GJS, Joffe JK, de Wit R, Aass N, Graham JD, Coleman R, Kirk SJ and Stenning SP | 2005 | Radiotherapy versus single-dose carboplatin in adjuvant treatment of stage I seminoma: a randomised trial. | Lancet (London, England) | 366 | 293-300 | <http://dx.doi.org/10.1016/S0140-6736(05)66984-X> |
|  | 2005 | One-dose carboplatin in seminoma. | Lancet (London, England) | 366 | 1526 | <http://dx.doi.org/10.1016/S0140-6736(05)67622-2> |
|  | 2005 | Maintenance of fertility following treatment with temozolomide for a high grade astrocytoma. | Journal of Neuro-Oncology | 73 | 185 | <http://dx.doi.org/10.1007/s11060-004-3577-y> |
|  | 2006 | Myths and facts on adjuvant carboplatin for stage I seminoma. | Journal of Clinical Oncology : official Journal of the American Society of Clinical Oncology | 24 | e40 | <http://dx.doi.org/10.1200/JCO.2006.05.7802> |
| Avramova B, Jordanova M, Michailov G, Konstantinov D, Christosova I and Bobev D | 2006 | Myeloablative chemotherapy with autologous peripheral blood stem cell transplantation in patients with poor-prognosis solid tumors - Bulgarian experience. | Journal of B.U.ON. : official Journal of the Balkan Union of Oncology | 11 | 433-438 |  |
| El-Helw LM, Naik JD, Chester JD, Joffe JK, Selby PJ and Coleman RE | 2006 | High-dose chemotherapy with haematopoietic stem-cell support in patients with poor prognosis, relapsed or refractory germ cell tumours. | BJU International | 98 | 519-525 | <http://dx.doi.org/10.1111/j.1464-410X.2006.06389.x> |
|  | 2006 | Carboplatin for stage I seminoma. | Journal of Clinical Oncology : official Journal of the American Society of Clinical Oncology | 24 | 2971-2973 | <http://dx.doi.org/10.1200/JCO.2005.05.3231> |
| Hara I, Miyake H, Yamada Y, Yamanaka K, Furukawa J, Kumano M, Takenaka A and Fujisawa M | 2006 | Feasibility and usefulness of high-dose chemotherapy (high-dose ifosfamide, carboplatin and etoposide) combined with peripheral blood stem cell transplantation for male germ cell tumor: a single-institute experience. | Anti-Cancer Drugs | 17 | 1057-1066 | <http://dx.doi.org/10.1097/01.cad.0000231469.46664.12> |
| Mathew P, Vakar-Lopez F and Troncoso P | 2006 | Protracted remission of metastatic epithelioid angiosarcoma with weekly infusion of doxorubicin, paclitaxel, and cisplatin. | The Lancet. Oncology | 7 | 92-93 | <http://dx.doi.org/10.1016/S1470-2045(05)70542-8> |
| Steiner H, Muller T, Gozzi C, Akkad T, Bartsch G and Berger AP | 2006 | Two cycles of cisplatin-based chemotherapy for low-volume retroperitoneal stage II nonseminomatous germ cell tumours. | BJU International | 98 | 349-352 | <http://dx.doi.org/10.1111/j.1464-410X.2006.06218.x> |
| Culine S, Kerbrat P, Kramar A, Theodore C, Chevreau C, Geoffrois L, Bui NB, Peny J, Caty A, Delva R, Biron P, Fizazi K, Bouzy J and Droz JP | 2007 | Refining the optimal chemotherapy regimen for good-risk metastatic nonseminomatous germ-cell tumors: a randomized trial of the Genito- Urinary Group of the French Federation of Cancer Centers (GETUG T93BP). | Annals of Oncology : official Journal of the European Society for Medical Oncology | 18 | 917-924 | <http://dx.doi.org/10.1093/annonc/mdm062> |
| Droz J-P, Kramar A, Biron P, Pico J-L, Kerbrat P, Peny J, Cure H, Chevreau C, Theodore C, Bouzy J and Culine S | 2007 | Failure of high-dose cyclophosphamide and etoposide combined with double- dose cisplatin and bone marrow support in patients with high-volume metastatic nonseminomatous germ-cell tumours: mature results of a randomised trial. | European Urology | 51 | 738-739 | <http://dx.doi.org/10.1016/j.eururo.2006.10.035> |
| Einhorn LH, Brames MJ, Juliar B and Williams SD | 2007 | Phase II study of paclitaxel plus gemcitabine salvage chemotherapy for germ cell tumors after progression following high-dose chemotherapy with tandem transplant. | Journal of Clinical Oncology : official Journal of the American Society of Clinical Oncology | 25 | 513-516 | <http://dx.doi.org/10.1200/JCO.2006.07.7271> |
| El Mesbahi O, Terrier-Lacombe M-J, Rebischung C, Theodore C, Vanel D and Fizazi K | 2007 | Chemotherapy in patients with teratoma with malignant transformation. | European Urology | 51 | 1302-1306 | <http://dx.doi.org/10.1016/j.eururo.2006.10.021> |
| Hartmann JT, Gauler T, Metzner B, Gerl A, Casper J, Rick O, Horger M, Schleicher J, Derigs G, Mayer-Steinacker R, Beyer J, Kuczyk MA and Bokemeyer C | 2007 | Phase I/II study of sequential dose-intensified ifosfamide, cisplatin, and etoposide plus paclitaxel as induction chemotherapy for poor prognosis germ cell tumors by the German Testicular Cancer Study Group. | Journal of Clinical Oncology : official Journal of the American Society of Clinical Oncology | 25 | 5742-5747 | <http://dx.doi.org/10.1200/JCO.2007.11.9099> |
| Kumano M, Miyake H, Hara I, Furukawa J, Takenaka A and Fujisawa M | 2007 | First-line high-dose chemotherapy combined with peripheral blood stem cell transplantation for patients with advanced extragonadal germ cell tumors. | International Journal of Urology : official Journal of the Japanese Urological Association | 14 | 336-338 | <http://dx.doi.org/10.1111/j.1442-2042.2006.01718.x> |
| Lorch A, Kollmannsberger C, Hartmann JT, Metzner B, Schmidt-Wolf IGH, Berdel WE, Weissinger F, Schleicher J, Egerer G, Haas A, Schirren R, Beyer J, Bokemeyer C and Rick O | 2007 | Single versus sequential high-dose chemotherapy in patients with relapsed or refractory germ cell tumors: a prospective randomized multicenter trial of the German Testicular Cancer Study Group. | Journal of Clinical Oncology : official Journal of the American Society of Clinical Oncology | 25 | 2778-2784 | <http://dx.doi.org/10.1200/JCO.2006.09.2148> |
| Mardiak J, Salek T, Sycova-Mila Z, Obertova J, Reckova M, Mego M, Hlavata Z, Brozmanova K, Risnyovzska Z, Svetlovska D and Koza I | 2007 | Paclitaxel, bleomycin, etoposide, and cisplatin (T-BEP) as initial treatment in patients with poor-prognosis germ cell tumors (GCT): a phase II study. | Neoplasma | 54 | 240-245 |  |
| Nakamura H, Takeshima H, Makino K and Kuratsu J-i | 2007 | Evaluation of residual tissues after adjuvant therapy in germ cell tumors. | Pediatric NeuroSurgery | 43 | 82-91 | <http://dx.doi.org/10.1159/000098378> |
| Nonomura N, Oka D, Nishimura K, Nakayama M, Inoue H, Mizutani Y, Miki T and Okuyama A | 2007 | Paclitaxel, ifosfamide, and nedaplatin (TIN) salvage chemotherapy for patients with advanced germ cell tumors. | International Journal of Urology : official Journal of the Japanese Urological Association | 14 | 527-531 | <http://dx.doi.org/10.1111/j.1442-2042.2006.01702.x> |
| Oechsle K, Honecker F, Kollmannsberger C, Rick O, Grunwald V, Mayer F, Hartmann JT and Bokemeyer C | 2007 | An open-label, multicenter phase II trial of capecitabine in patients with cisplatin-refractory or relapsed germ cell tumors. | Anti-Cancer Drugs | 18 | 273-276 | <http://dx.doi.org/10.1097/CAD.0b013e328011fd99> |
| Silva CAA and Brunner HI | 2007 | Gonadal functioning and preservation of reproductive fitness with juvenile systemic lupus erythematosus. | Lupus | 16 | 593-599 | <http://dx.doi.org/10.1177/0961203307077538> |
| van Beek RD, Smit M, van den Heuvel-Eibrink MM, de Jong FH, Hakvoort-Cammel FG, van den Bos C, van den Berg H, Weber RFA, Pieters R and de Muinck Keizer-Schrama SMPF | 2007 | Inhibin B is superior to FSH as a serum marker for spermatogenesis in men treated for Hodgkin's lymphoma with chemotherapy during childhood. | Human Reproduction (Oxford, England) | 22 | 3215-3222 | <http://dx.doi.org/10.1093/humrep/dem313> |
| van der Kaaij MAE, Heutte N, Le Stang N, Raemaekers JMM, Simons AHM, Carde P, Noordijk EM, Ferme C, Thomas J, Eghbali H, Kluin-Nelemans HC and Henry-Amar M | 2007 | Gonadal function in males after chemotherapy for early-stage Hodgkin's lymphoma treated in four subsequent trials by the European Organisation for Research and Treatment of Cancer: EORTC Lymphoma Group and the Groupe d'Etude des Lymphomes de l'Adulte. | Journal of Clinical Oncology : official Journal of the American Society of Clinical Oncology | 25 | 2825-2832 | <http://dx.doi.org/10.1200/JCO.2006.10.2020> |
| Vural F, Cagirgan S, Saydam G, Hekimgil M, Soyer NA and Tombuloglu M | 2007 | Primary testicular lymphoma. | Journal of the National Medical Association | 99 | 1277-1282 |  |
| Bokemeyer C, Oechsle K, Honecker F, Mayer F, Hartmann JT, Waller CF, Bohlke I and Kollmannsberger C | 2008 | Combination chemotherapy with gemcitabine, oxaliplatin, and paclitaxel in patients with cisplatin-refractory or multiply relapsed germ-cell tumors: a study of the German Testicular Cancer Study Group. | Annals of Oncology : official Journal of the European Society for Medical Oncology | 19 | 448-453 | <http://dx.doi.org/10.1093/annonc/mdm526> |
| Cresswell J, Scheitlin W, Gozen A, Lenz E, Teber D and Rassweiler J | 2008 | Laparoscopic retroperitoneal lymph node dissection combined with adjuvant chemotherapy for pathological stage II Disease in nonseminomatous germ cell tumours: a 15-year experience. | BJU International | 102 | 844-848 | <http://dx.doi.org/10.1111/j.1464-410X.2008.07754.x> |
| Culine S, Kramar A, Theodore C, Geoffrois L, Chevreau C, Biron P, Nguyen BB, Heron J-F, Kerbrat P, Caty A, Delva R, Fargeot P, Fizazi K, Bouzy J and Droz J-P | 2008 | Randomized trial comparing bleomycin/etoposide/cisplatin with alternating cisplatin/cyclophosphamide/doxorubicin and vinblastine/bleomycin regimens of chemotherapy for patients with intermediate- and poor-risk metastatic nonseminomatous germ cell tumors: | Journal of Clinical Oncology : official Journal of the American Society of Clinical Oncology | 26 | 421-427 | <http://dx.doi.org/10.1200/JCO.2007.13.8461> |
| Gremmer R, Schroder MLJF, Ten Huinink WWB, Brandsma D and Boogerd W | 2008 | Successful management of brain metastasis from malignant germ cell tumours with standard induction chemotherapy. | Journal of Neuro-Oncology | 90 | 335-339 | <http://dx.doi.org/10.1007/s11060-008-9668-4> |
|  | 2008 | Stage I seminoma and carboplatin risks. | Annals of Oncology : official Journal of the European Society for Medical Oncology | 19 | 407-408 | <http://dx.doi.org/10.1093/annonc/mdm594> |
| Kesler KA, Rieger KM, Hammoud ZT, Kruter LE, Perkins SM, Turrentine MW, Schneider BP, Einhorn LH and Brown JW | 2008 | A 25-year single institution experience with surgery for primary mediastinal nonseminomatous germ cell tumors. | The Annals of Thoracic Surgery | 85 | 371-378 | <http://dx.doi.org/10.1016/j.athoracsur.2007.09.020> |
| Lopes LF, Sonaglio V, Ribeiro KCB, Schneider DT and de Camargo B | 2008 | Improvement in the outcome of children with germ cell tumors. | Pediatric Blood & Cancer | 50 | 250-253 | <http://dx.doi.org/10.1002/pbc.21268> |
| Oechsle K, Kollmannsberger C, Honecker F, Boehlke I and Bokemeyer C | 2008 | Cerebral metastases in non-seminomatous germ cell tumour patients undergoing primary high-dose chemotherapy. | European Journal of Cancer (Oxford, England : 1990) | 44 | 1663-1669 | <http://dx.doi.org/10.1016/j.ejca.2008.05.012> |
| Sammler C, Beyer J, Bokemeyer C, Hartmann JT and Rick O | 2008 | Risk factors in germ cell tumour patients with relapse or progressive Disease after first-line chemotherapy: evaluation of a prognostic score for survival after high-dose chemotherapy. | European Journal of Cancer (Oxford, England : 1990) | 44 | 237-243 | <http://dx.doi.org/10.1016/j.ejca.2007.10.025> |
| Theodore C, Chevreau C, Yataqhene Y, Fizazi K, Delord J-P, Lotz J-P, Geoffrois L, Kerbrat P, Bui V and Flechon A | 2008 | A phase II multicenter study of oxaliplatin in combination with paclitaxel in poor prognosis patients who failed cisplatin-based chemotherapy for germ-cell tumors. | Annals of Oncology : official Journal of the European Society for Medical Oncology | 19 | 1465-1469 | <http://dx.doi.org/10.1093/annonc/mdn122> |
| Agarwal R, Dvorak CC, Stockerl-Goldstein KE, Johnston L and Srinivas S | 2009 | High-dose chemotherapy followed by stem cell rescue for high-risk germ cell tumors: the Stanford experience. | Bone Marrow Transplantation | 43 | 547-552 | <http://dx.doi.org/10.1038/bmt.2008.364> |
| Armstrong GT, Liu Q, Yasui Y, Neglia JP, Leisenring W, Robison LL and Mertens AC | 2009 | Late mortality among 5-year survivors of childhood cancer: a summary from the Childhood Cancer Survivor Study. | Journal of Clinical Oncology : official Journal of the American Society of Clinical Oncology | 27 | 2328-2338 | <http://dx.doi.org/10.1200/JCO.2008.21.1425> |
| Giannis M, Aristotelis B, Vassiliki K, Ioannis A, Konstantinos S, Nikolaos A, Georgios P, Georgios P, Pantelis P and Meletios-Athanasios D | 2009 | Cisplatin-based chemotherapy for advanced seminoma: report of 52 cases treated in two institutions. | Journal of Cancer Research and Clinical Oncology | 135 | 1495-1500 | <http://dx.doi.org/10.1007/s00432-009-0596-2> |
|  | 2009 | Treating IIA/B seminoma with combination carboplatin and radiotherapy. | Journal of Clinical Oncology : official Journal of the American Society of Clinical Oncology | 27 | 2101-2103 | <http://dx.doi.org/10.1200/JCO.2008.21.5269> |
| Green DM, Sklar CA, Boice JDJ, Mulvihill JJ, Whitton JA, Stovall M and Yasui Y | 2009 | Ovarian failure and reproductive outcomes after childhood cancer treatment: results from the Childhood Cancer Survivor Study. | Journal of Clinical Oncology : official Journal of the American Society of Clinical Oncology | 27 | 2374-2381 | <http://dx.doi.org/10.1200/JCO.2008.21.1839> |
| Kiserud CE, Fossa A, Bjoro T, Holte H, Cvancarova M and Fossa SD | 2009 | Gonadal function in male patients after treatment for malignant lymphomas, with emphasis on chemotherapy. | British Journal of Cancer | 100 | 455-463 | <http://dx.doi.org/10.1038/sj.bjc.6604892> |
| Lopes LF, Macedo CRP, Pontes EM, Dos Santos Aguiar S, Mastellaro MJ, Melaragno R, Vianna SMR, Lopes PAA, Mendonca N, de Assis Almeida MT, Sonaglio V, Ribeiro KB, Santana VM, Schneider DT and de Camargo B | 2009 | Cisplatin and etoposide in childhood germ cell tumor: brazilian pediatric oncology society protocol GCT-91. | Journal of Clinical Oncology : official Journal of the American Society of Clinical Oncology | 27 | 1297-1303 | <http://dx.doi.org/10.1200/JCO.2008.16.4202> |
| Nakamura Y, Matsumura A, Katsura H, Sakaguchi M, Ito N, Kitahara N, Ose N and Kitaichi M | 2009 | Cisplatin-based chemotherapy followed by surgery for malignant nonseminomatous germ cell tumor of mediastinum: one institution's experience. | General Thoracic and Cardiovascular Surgery | 57 | 363-368 | <http://dx.doi.org/10.1007/s11748-008-0375-z> |
| Nurmio M, Keros V, Lahteenmaki P, Salmi T, Kallajoki M and Jahnukainen K | 2009 | Effect of childhood acute lymphoblastic leukemia therapy on spermatogonia populations and future fertility. | The Journal of Clinical Endocrinology and Metabolism | 94 | 2119-2122 | <http://dx.doi.org/10.1210/jc.2009-0060> |
| Riese MJ and Vaughn DJ | 2009 | Chemotherapy for patients with poor prognosis germ cell tumors. | World Journal of Urology | 27 | 471-476 | <http://dx.doi.org/10.1007/s00345-009-0404-2> |
| Shiraishi T, Nakamura T, Mikami K, Takaha N, Kawauchi A and Miki T | 2009 | Salvage chemotherapy with paclitaxel and gemcitabine plus nedaplatin (TGN) as part of multidisciplinary therapy in patients with heavily pretreated cisplatin-refractory germ cell tumors. | International Journal of Clinical Oncology | 14 | 436-441 | <http://dx.doi.org/10.1007/s10147-009-0899-y> |
| van Casteren NJ, van der Linden GHM, Hakvoort-Cammel FGAJ, Hahlen K, Dohle GR and van den Heuvel-Eibrink MM | 2009 | Effect of childhood cancer treatment on fertility markers in adult male long-term survivors. | Pediatric Blood & Cancer | 52 | 108-112 | <http://dx.doi.org/10.1002/pbc.21780> |
| Afzal S, Wherrett D, Bartels U, Tabori U, Huang A, Stephens D and Bouffet E | 2010 | Challenges in management of patients with intracranial germ cell tumor and diabetes insipidus treated with cisplatin and/or ifosfamide based chemotherapy. | Journal of neuro-Oncology | 97 | 393-399 | <http://dx.doi.org/10.1007/s11060-009-0033-z> |
| Alapetite C, Brisse H, Patte C, Raquin MA, Gaboriaud G, Carrie C, Habrand JL, Thiesse P, Cuilliere JC, Bernier V, Ben-Hassel M, Frappaz D, Baranzelli MC and Bouffet E | 2010 | Pattern of relapse and outcome of non-metastatic germinoma patients treated with chemotherapy and limited field radiation: the SFOP experience. | Neuro-Oncology | 12 | 1318-1325 | <http://dx.doi.org/10.1093/neuonc/noq093> |
| Cai J-Y, Tang J-Y, Pan C, Xu M, Xue H-L, Zhou M, Dong L, Ye Q-D, Jiang H, Shen S-H and Chen J | 2010 | Results of RS-99 protocol for childhood solid tumors. | World Journal of Pediatrics : WJP | 6 | 43-49 | <http://dx.doi.org/10.1007/s12519-010-0005-6> |
| Chatzidarellis E, Makrilia N, Giza L, Georgiadis E, Alamara C and Syrigos KN | 2010 | Effects of taxane-based chemotherapy on inhibin B and gonadotropins as biomarkers of spermatogenesis. | Fertility and Sterility | 94 | 558-563 | <http://dx.doi.org/10.1016/j.fertnstert.2009.03.068> |
| da Silva NS, Cappellano AM, Diez B, Cavalheiro S, Gardner S, Wisoff J, Kellie S, Parker R, Garvin J and Finlay J | 2010 | Primary chemotherapy for intracranial germ cell tumors: results of the third international CNS germ cell tumor study. | Pediatric Blood & Cancer | 54 | 377-383 | <http://dx.doi.org/10.1002/pbc.22381> |
| Ehrlich Y, Beck SDW, Ulbright TM, Cheng L, Brames MJ, Andreoiu M, Foster RS and Einhorn LH | 2010 | Outcome analysis of patients with transformed teratoma to primitive neuroectodermal tumor. | Annals of Oncology : official Journal of the European Society for Medical Oncology | 21 | 1846-1850 | <http://dx.doi.org/10.1093/annonc/mdq045> |
| Ehrlich Y, Brames MJ, Beck SDW, Foster RS and Einhorn LH | 2010 | Long-term follow-up of Cisplatin combination chemotherapy in patients with disseminated nonseminomatous germ cell tumors: is a postchemotherapy retroperitoneal lymph node dissection needed after complete remission? | Journal of Clinical Oncology : official Journal of the American Society of Clinical Oncology | 28 | 531-536 | <http://dx.doi.org/10.1200/JCO.2009.23.0714> |
| Flechon A, Tavernier E, Boyle H, Meeus P, Rivoire M and Droz J-P | 2010 | Long-term oncological outcome after post-chemotherapy retroperitoneal lymph node dissection in men with metastatic nonseminomatous germ cell tumour. | BJU International | 106 | 779-785 | <http://dx.doi.org/10.1111/j.1464-410X.2009.09175.x> |
| Gobel U, Schneider DT, Teske C, Schonberger S and Calaminus G | 2010 | Brain metastases in children and adolescents with extracranial germ cell tumor - data of the MAHO/MAKEI-registry. | Klinische Padiatrie | 222 | 140-144 | <http://dx.doi.org/10.1055/s-0030-1249661> |
| Green DM, Kawashima T, Stovall M, Leisenring W, Sklar CA, Mertens AC, Donaldson SS, Byrne J and Robison LL | 2010 | Fertility of male survivors of childhood cancer: a report from the Childhood Cancer Survivor Study. | Journal of Clinical Oncology : official Journal of the American Society of Clinical Oncology | 28 | 332-339 | <http://dx.doi.org/10.1200/JCO.2009.24.9037> |
| Grimison PS, Stockler MR, Thomson DB, Olver IN, Harvey VJ, Gebski VJ, Lewis CR, Levi JA, Boyer MJ, Gurney H, Craft P, Boland AL, Simes RJ and Toner GC | 2010 | Comparison of two standard chemotherapy regimens for good-prognosis germ cell tumors: updated analysis of a randomized trial. | Journal of the National Cancer Institute | 102 | 1253-1262 | <http://dx.doi.org/10.1093/jnci/djq245> |
| Hudson MM | 2010 | Reproductive outcomes for survivors of childhood cancer. | Obstetrics and Gynecology | 116 | 1171-1183 | <http://dx.doi.org/10.1097/AOG.0b013e3181f87c4b> |
| Lorch A, Beyer J, Bascoul-Mollevi C, Kramar A, Einhorn LH, Necchi A, Massard C, De Giorgi U, Flechon A, Margolin KA, Lotz J-P, Germa Lluch JR, Powles T and Kollmannsberger CK | 2010 | Prognostic factors in patients with metastatic germ cell tumors who experienced treatment failure with cisplatin-based first-line chemotherapy. | Journal of Clinical Oncology : official Journal of the American Society of Clinical Oncology | 28 | 4906-4911 | <http://dx.doi.org/10.1200/JCO.2009.26.8128> |
| Lorch A, Rick O, Wundisch T, Hartmann J-T, Bokemeyer C and Beyer J | 2010 | High dose chemotherapy as salvage treatment for unresectable late relapse germ cell tumors. | The Journal of Urology | 184 | 168-173 | <http://dx.doi.org/10.1016/j.juro.2010.03.017> |
| Mazloom A, Fowler N, Medeiros LJ, Iyengar P, Horace P and Dabaja BS | 2010 | Outcome of patients with diffuse large B-cell lymphoma of the testis by era of treatment: the M. D. Anderson Cancer Center experience. | Leukemia & Lymphoma | 51 | 1217-1224 | <http://dx.doi.org/10.3109/10428191003793358> |
| Pectasides D, Pectasides E, Papaxoinis G, Xiros N, Kamposioras K, Tountas N and Economopoulos T | 2010 | Methotrexate, paclitaxel, ifosfamide, and cisplatin in poor-risk nonseminomatous germ cell tumors. | Urologic Oncology | 28 | 617-623 | <http://dx.doi.org/10.1016/j.urolonc.2008.10.013> |
| Radaideh SM, Cook VC, Kesler KA and Einhorn LH | 2010 | Outcome following resection for patients with primary mediastinal nonseminomatous germ-cell tumors and rising serum tumor markers post- chemotherapy. | Annals of Oncology : official Journal of the European Society for Medical Oncology | 21 | 804-807 | <http://dx.doi.org/10.1093/annonc/mdp516> |
| Rosenfeld A, Kletzel M, Duerst R, Jacobsohn D, Haut P, Weinstein J, Rademaker A, Schaefer C, Evans L, Fouts M and Goldman S | 2010 | A phase II prospective study of sequential myeloablative chemotherapy with hematopoietic stem cell rescue for the treatment of selected high risk and recurrent central nervous system tumors. | Journal of Neuro-Oncology | 97 | 247-255 | <http://dx.doi.org/10.1007/s11060-009-0009-z> |
| Tanaka T, Kitamura H, Takahashi A, Masumori N and Tsukamoto T | 2010 | Long-term outcome of chemotherapy for advanced testicular and extragonadal germ cell tumors: a single-center 27-year experience. | Japanese Journal of Clinical Oncology | 40 | 73-78 | <http://dx.doi.org/10.1093/jjco/hyp121> |
| Yoo KH, Lee SH, Lee J, Sung KW, Jung HL, Koo HH, Lim DH, Kim JH and Shin HJ | 2010 | Improved outcome of central nervous system germ cell tumors: implications for the role of risk-adapted intensive chemotherapy. | Journal of Korean Medical Science | 25 | 458-465 | <http://dx.doi.org/10.3346/jkms.2010.25.3.458> |
| Yun J, Kim SJ, Kim JA, Kong JH, Lee SH, Kim K, Ko YH and Kim WS | 2010 | Clinical features and treatment outcomes of non-Hodgkin's lymphomas involving rare extranodal sites: a single-center experience. | Acta Haematologica | 123 | 48-54 | <http://dx.doi.org/10.1159/000262291> |
| Zaletel LZ, Bratanic N and Jereb B | 2010 | Gonadal function in patients treated for Hodgkin's Disease in childhood. | Radiology and Oncology | 44 | 187-193 | <http://dx.doi.org/10.2478/v10019-010-0034-8> |
| Agarwala AK, Perkins SM, Abonour R, Brames MJ and Einhorn LH | 2011 | Salvage chemotherapy with high-dose carboplatin and etoposide with peripheral blood stem cell transplant in patients with relapsed pure seminoma. | American Journal of Clinical Oncology | 34 | 286-288 | <http://dx.doi.org/10.1097/COC.0b013e3181d6b518> |
|  | 2011 | Carboplatin in clinical stage I seminoma: too much and too little at the same time. | Journal of Clinical Oncology : official Journal of the American Society of Clinical Oncology | 29 | 949-952 | <http://dx.doi.org/10.1200/JCO.2010.29.5055> |
| Daugaard G, Skoneczna I, Aass N, De Wit R, De Santis M, Dumez H, Marreaud S, Collette L, Lluch JRG, Bokemeyer C and Schmoll HJ | 2011 | A randomized phase III study comparing standard dose BEP with sequential high-dose cisplatin, etoposide, and ifosfamide (VIP) plus stem-cell support in males with poor-prognosis germ-cell cancer. An intergroup study of EORTC, GTCSG, and Grupo Germinal (EO | Annals of Oncology : official Journal of the European Society for Medical Oncology | 22 | 1054-1061 | <http://dx.doi.org/10.1093/annonc/mdq575> |
| Harel S, Ferme C and Poirot C | 2011 | Management of fertility in patients treated for Hodgkin's lymphoma. | Haematologica | 96 | 1692-1699 | <http://dx.doi.org/10.3324/haematol.2011.045856> |
| Hendricks M, Davidson A, Pillay K, Desai F and Millar A | 2011 | Carboplatin-based chemotherapy and surgery: a cost effective treatment strategy for malignant extracranial germ cell tumours in the developing world. | Pediatric Blood & Cancer | 57 | 172-174 | <http://dx.doi.org/10.1002/pbc.23055> |
| Koychev D, Oechsle K, Bokemeyer C and Honecker F | 2011 | Treatment of patients with relapsed and/or cisplatin-refractory metastatic germ cell tumours: an update. | International Journal of Andrology | 34 | e266-73 | <http://dx.doi.org/10.1111/j.1365-2605.2011.01145.x> |
| Mead GM, Fossa SD, Oliver RTD, Joffe JK, Huddart RA, Roberts JT, Pollock P, Gabe R and Stenning SP | 2011 | Randomized trials in 2466 patients with stage I seminoma: patterns of relapse and follow-up. | Journal of the National Cancer Institute | 103 | 241-249 | <http://dx.doi.org/10.1093/jnci/djq525> |
| Oechsle K, Honecker F, Cheng T, Mayer F, Czaykowski P, Winquist E, Wood L, Fenner M, Glaesener S, Hartmann JT, Chi K, Bokemeyer C and Kollmannsberger C | 2011 | Preclinical and clinical activity of sunitinib in patients with cisplatin-refractory or multiply relapsed germ cell tumors: a Canadian Urologic Oncology Group/German Testicular Cancer Study Group cooperative study. | Annals of Oncology : official Journal of the European Society for Medical Oncology | 22 | 2654-2660 | <http://dx.doi.org/10.1093/annonc/mdr026> |
| Oechsle K, Kollmannsberger C, Honecker F, Mayer F, Waller CF, Hartmann JT, Boehlke I and Bokemeyer C | 2011 | Long-term survival after treatment with gemcitabine and oxaliplatin with and without paclitaxel plus secondary surgery in patients with cisplatin- refractory and/or multiply relapsed germ cell tumors. | European Urology | 60 | 850-855 | <http://dx.doi.org/10.1016/j.eururo.2011.06.019> |
| Park S, Lee S, Lee J, Park SH, Park JO, Kang WK, Park YS and Lim HY | 2011 | Salvage chemotherapy with paclitaxel, ifosfamide, and cisplatin (TIP) in relapsed or cisplatin-refractory germ cell tumors. | Onkologie | 34 | 416-420 | <http://dx.doi.org/10.1159/000331129> |
| Takahashi S, Yoshida K and Kawase T | 2011 | Intracranial germ cell tumors: efficacy of neoadjuvant chemo-radiotherapy without surgical biopsy. | The Keio Journal of Medicine | 60 | 56-64 |  |
| Tryakin A, Fedyanin M, Kanagavel D, Fainstein I, Sergeev J, Polockij B, Matveev V, Zakharova T, Garin A and Tjulandin S | 2011 | Paclitaxel+BEP (T-BEP) regimen as induction chemotherapy in poor prognosis patients with nonseminomatous germ cell tumors: a phase II study. | Urology | 78 | 620-625 | <http://dx.doi.org/10.1016/j.urology.2011.05.005> |
| De Corti F, Sarnacki S, Patte C, Mosseri V, Baranzelli MC, Martelli H, Conter C, Frappaz D and Orbach D | 2012 | Prognosis of malignant sacrococcygeal germ cell tumours according to their natural history and surgical management. | Surgical Oncology | 21 | e31-7 | <http://dx.doi.org/10.1016/j.suronc.2012.03.001> |
| Gombos DS | 2012 | Retinoblastoma in the perinatal and neonatal child. | Seminars in Fetal & Neonatal Medicine | 17 | 239-242 | <http://dx.doi.org/10.1016/j.siny.2012.04.003> |
| Lorch A, Kleinhans A, Kramar A, Kollmannsberger CK, Hartmann JT, Bokemeyer C, Rick O and Beyer J | 2012 | Sequential versus single high-dose chemotherapy in patients with relapsed or refractory germ cell tumors: long-term results of a prospective randomized trial. | Journal of Clinical Oncology : official Journal of the American Society of Clinical Oncology | 30 | 800-805 | <http://dx.doi.org/10.1200/JCO.2011.38.6391> |
| Patterson BC, Wasilewski-Masker K, Ryerson AB, Mertens A and Meacham L | 2012 | Endocrine health problems detected in 519 patients evaluated in a pediatric cancer survivor program. | The Journal of Clinical Endocrinology and Metabolism | 97 | 810-818 | <http://dx.doi.org/10.1210/jc.2011-2104> |
| van der Kaaij MAE, Heutte N, Meijnders P, Abeilard-Lemoisson E, Spina M, Moser LC, Allgeier A, Meulemans B, Dubois B, Simons AHM, Lugtenburg PJ, Aleman BMP, Noordijk EM, Ferme C, Thomas J, Stamatoullas A, Fruchart C, Brice P, Gaillard I, Doorduijn JK, Sebban C, Smit WGJM, Bologna S, Roesink JM, Ong F, Andre MPE, Raemaekers JMM, Henry-Amar M and Kluin-Nelemans HC | 2012 | Parenthood in survivors of Hodgkin lymphoma: an EORTC-GELA general population case-control study. | Journal of Clinical Oncology : official Journal of the American Society of Clinical Oncology | 30 | 3854-3863 | <http://dx.doi.org/10.1200/JCO.2011.40.8906> |
| van Dorp W, van Beek RD, Laven JSE, Pieters R, de Muinck Keizer-Schrama SMPF and van den Heuvel-Eibrink MM | 2012 | Long-term endocrine side effects of childhood Hodgkin's lymphoma treatment: a review. | Human Reproduction Update | 18 | Dec-28 | <http://dx.doi.org/10.1093/humupd/dmr038> |
| Baek HJ, Park HJ, Sung KW, Lee SH, Han JW, Koh KN, Im HJ, Kang HJ and Park KD | 2013 | Myeloablative chemotherapy and autologous stem cell transplantation in patients with relapsed or progressed central nervous system germ cell tumors: results of Korean Society of Pediatric Neuro-Oncology (KSPNO) S-053 study. | Journal of Neuro-Oncology | 114 | 329-338 | <http://dx.doi.org/10.1007/s11060-013-1188-1> |
| Boyle HJ, Jouanneau E, Droz JP and Flechon A | 2013 | Management of brain metastases from germ cell tumors: a single center experience. | Oncology | 85 | 21-26 | <http://dx.doi.org/10.1159/000351812> |
| Buyukpamukcu M, Varan A, Kupeli S, Ekinci S, Yalcin S, Kale G, Yalcin B, Kutluk T and Akyuz C | 2013 | Malignant sacrococcygeal germ cell tumors in children: a 30-year experience from a single institution. | Tumori | 99 | 51-56 | <http://dx.doi.org/10.1700/1248.13788> |
| Fukui N, Kohno Y, Ishioka J-I, Fukuda H, Kageyama Y and Higashi Y | 2013 | Treatment outcome of patients with extragonadal nonseminomatous germ cell tumors: the Saitama Cancer Center experience. | International Journal of Clinical Oncology | 18 | 731-734 | <http://dx.doi.org/10.1007/s10147-012-0436-2> |
| Giesen E, Mager A, van Tinteren H, Rodenhuis S and Kerst JM | 2013 | An alternative treatment regimen of advanced seminoma with carboplatin, etoposide, and bleomycin instead of cisplatin-based therapy. | Urologic Oncology | 31 | 110-114 | <http://dx.doi.org/10.1016/j.urolonc.2010.10.006> |
| Ismail H, Dembowska-Baginska B, Broniszczak D, Kalicinski P, Maruszewski P, Kluge P, Swieszkowska E, Kosciesza A, Lembas A and Perek D | 2013 | Treatment of undifferentiated embryonal sarcoma of the liver in children --single center experience. | Journal of Pediatric Surgery | 48 | 2202-2206 | <http://dx.doi.org/10.1016/j.jpedsurg.2013.05.020> |
| Lee SH and Shin CH | 2013 | Reduced male fertility in childhood cancer survivors. | Annals of Pediatric endocrinology & metabolism | 18 | 168-172 | <http://dx.doi.org/10.6065/apem.2013.18.4.168> |
| Mascarenhas L, Malogolowkin M, Armenian SH, Sposto R and Venkatramani R | 2013 | A phase I study of oxaliplatin and doxorubicin in pediatric patients with relapsed or refractory extracranial non-hematopoietic solid tumors. | Pediatric Blood & Cancer | 60 | 1103-1107 | <http://dx.doi.org/10.1002/pbc.24471> |
| Necchi A, Nicolai N, Mariani L, Raggi D, Fare E, Giannatempo P, Catanzaro M, Biasoni D, Torelli T, Stagni S, Milani A, Piva L, Pizzocaro G, Gianni AM and Salvioni R | 2013 | Modified cisplatin, etoposide, and ifosfamide (PEI) salvage therapy for male germ cell tumors: long-term efficacy and safety outcomes. | Annals of Oncology : official Journal of the European Society for Medical Oncology | 24 | 2887-2892 | <http://dx.doi.org/10.1093/annonc/mdt271> |
| Shaikh F, Nathan PC, Hale J, Uleryk E and Frazier L | 2013 | Is there a role for carboplatin in the treatment of malignant germ cell tumors? A systematic review of adult and pediatric trials. | Pediatric Blood & Cancer | 60 | 587-592 | <http://dx.doi.org/10.1002/pbc.24288> |
| Suleiman Y, Siddiqui BK, Brames MJ, Abonour R and Einhorn LH | 2013 | Salvage therapy with high-dose chemotherapy and peripheral blood stem cell transplant in patients with primary mediastinal nonseminomatous germ cell tumors. | Biology of Blood and Marrow Transplantation : Journal of the American Society for Blood and Marrow Transplantation | 19 | 161-163 | <http://dx.doi.org/10.1016/j.bbmt.2012.08.002> |
| Tanaka H, Yuasa T, Fujii Y, Sakura M, Urakami S, Yamamoto S, Masuda H, Fukui I and Yonese J | 2013 | First-line combination chemotherapy with cisplatin, etoposide and ifosfamide for the treatment of disseminated germ cell cancer: re-evaluation in the granulocyte colony-stimulating factor era. | Chemotherapy | 59 | 441-446 | <http://dx.doi.org/10.1159/000362498> |
| Wessalowski R, Schneider DT, Mils O, Friemann V, Kyrillopoulou O, Schaper J, Matuschek C, Rothe K, Leuschner I, Willers R, Schonberger S, Gobel U and Calaminus G | 2013 | Regional deep hyperthermia for salvage treatment of children and adolescents with refractory or recurrent non-testicular malignant germ- cell tumours: an open-label, non-randomised, single-institution, phase 2 study. | The Lancet. Oncology | 14 | 843-852 | <http://dx.doi.org/10.1016/S1470-2045(13)70271-7> |
| Aparicio J, Maroto P, Garcia del Muro X, Sanchez-Munoz A, Guma J, Margeli M, Saenz A, Sagastibelza N, Castellano D, Arranz JA, Hervas D, Bastus R, Fernandez-Aramburo A, Sastre J, Terrasa J, Lopez-Brea M, Dorca J, Almenar D, Carles J, Hernandez A and Germa JR | 2014 | Prognostic factors for relapse in stage I seminoma: a new nomogram derived from three consecutive, risk-adapted studies from the Spanish Germ Cell Cancer Group (SGCCG). | Annals of Oncology : official Journal of the European Society for Medical Oncology | 25 | 2173-2178 | <http://dx.doi.org/10.1093/annonc/mdu437> |
| Berger LA, Bokemeyer C, Lorch A, Hentrich M, Kopp H-G, Gauler TC, Beyer J, de Wit M, Mayer F, Boehlke I, Oing C, Honecker F and Oechsle K | 2014 | First salvage treatment in patients with advanced germ cell cancer after cisplatin-based chemotherapy: analysis of a registry of the German Testicular Cancer Study Group (GTCSG). | Journal of Cancer Research and Clinical Oncology | 140 | 1211-1220 | <http://dx.doi.org/10.1007/s00432-014-1661-z> |
| Faure-Conter C, Orbach D, Cropet C, Baranzelli MC, Martelli H, Thebaud E, Verite C, Rome A, Fasola S, Corradini N, Rocourt N, Frappaz D, Kalfa N and Patte C | 2014 | Salvage therapy for refractory or recurrent pediatric germ cell tumors: the French SFCE experience. | Pediatric Blood & Cancer | 61 | 253-259 | <http://dx.doi.org/10.1002/pbc.24730> |
| Fedyanin M, Tryakin A, Mosyakova Y, Pokataev I, Bulanov A, Zakharova T, Polockii B, Garin A and Tjulandin S | 2014 | Prognostic factors and efficacy of different chemotherapeutic regimens in patients with mediastinal nonseminomatous germ cell tumors. | Journal of Cancer Research and Clinical Oncology | 140 | 311-318 | <http://dx.doi.org/10.1007/s00432-013-1567-1> |
| Fizazi K, Delva R, Caty A, Chevreau C, Kerbrat P, Rolland F, Priou F, Geoffrois L, Rixe O, Beuzeboc P, Malhaire J-P, Culine S, Aubelle M-S and Laplanche A | 2014 | A risk-adapted study of cisplatin and etoposide, with or without ifosfamide, in patients with metastatic seminoma: results of the GETUG S99 multicenter prospective study. | European Urology | 65 | 381-386 | <http://dx.doi.org/10.1016/j.eururo.2013.09.004> |
| Gobel U, Calaminus G, Haas R, Teske C, Schonberger S, Schneider DT, Leuschner I and Harms D | 2014 | Testicular germ cell tumors in adolescents - results of the protocol MAHO 98 and the identification of good risk patients. | Klinische Padiatrie | 226 | 316-322 | <http://dx.doi.org/10.1055/s-0034-1387748> |
| Grimison PS, Stockler MR, Chatfield M, Thomson DB, Gebski V, Friedlander M, Boland AL, Houghton B, Gurney H, Rosenthal M, Singhal N, Kichenadasse G, Wong SS, Lewis CR, Vasey PA and Toner GC | 2014 | Accelerated BEP for metastatic germ cell tumours: a multicenter phase II trial by the Australian and New Zealand Urogenital and Prostate Cancer Trials Group (ANZUP). | Annals of Oncology : official Journal of the European Society for Medical Oncology | 25 | 143-148 | <http://dx.doi.org/10.1093/annonc/mdt369> |
| Haugnes HS, Solhaug O, Stenberg J, Hjelle LV and Bremnes RM | 2014 | Seminoma patients treated at a minor oncological department during 1986-2010: treatment and outcome. | AntiCancer Research | 34 | 4253-4260 |  |
| Kenney LB, Duffey-Lind E, Ebb D, Sklar CA, Grier H and Diller L | 2014 | Impaired testicular function after an ifosfamide-containing regimen for pediatric osteosarcoma: a case series and review of the literature. | Journal of Pediatric Hematology/Oncology | 36 | 237-240 | <http://dx.doi.org/10.1097/MPH.0b013e3182a27c39> |
| Mihaljevic B, Vukovic V, Smiljanic M, Milic N, Todorovic M, Bila J, Andjelic B, Djurasinovic V, Jelicic J and Antic D | 2014 | Single-center experience in the treatment of primary testicular lymphoma. | Oncology Research and Treatment | 37 | 239-242 | <http://dx.doi.org/10.1159/000362399> |
| Robertson PL, Jakacki R, Hukin J, Siffert J and Allen JC | 2014 | Multimodality therapy for CNS mixed malignant germ cell tumors (MMGCT): results of a phase II multi-institutional study. | Journal of Neuro-Oncology | 118 | 93-100 | <http://dx.doi.org/10.1007/s11060-013-1306-0> |
| Shiraishi K and Matsuyama H | 2014 | Microdissection testicular sperm extraction and salvage hormonal treatment in patients with postchemotherapy azoospermia. | Urology | 83 | 100-106 | <http://dx.doi.org/10.1016/j.urology.2013.08.043> |
| Sun X-F, Zhang F, Zhen Z-J, Yang Q-Y, Xia Y-F, Wu S-X, Zhu J, Lu S-Y, Wang J, Sun F-F, Cai R-Q, Chen Y and Li P-F | 2014 | The clinical characteristics and treatment outcome of 57 children and adolescents with primary central nervous system germ cell tumors. | Chinese Journal of Cancer | 33 | 395-401 | <http://dx.doi.org/10.5732/cjc.013.10112> |
| Tandstad T, Stahl O, Hakansson U, Dahl O, Haugnes HS, Klepp OH, Langberg CW, Laurell A, Oldenburg J, Solberg A, Soderstrom K, Cavallin-Stahl E, Stierner U, Wahlquist R, Wall N and Cohn-Cedermark G | 2014 | One course of adjuvant BEP in clinical stage I nonseminoma mature and expanded results from the SWENOTECA group. | Annals of Oncology : official Journal of the European Society for Medical Oncology | 25 | 2167-2172 | <http://dx.doi.org/10.1093/annonc/mdu375> |
| Wilhelmsson M, Vatanen A, Borgstrom B, Gustafsson B, Taskinen M, Saarinen-Pihkala UM, Winiarski J and Jahnukainen K | 2014 | Adult testicular volume predicts spermatogenetic recovery after allogeneic HSCT in childhood and adolescence. | Pediatric Blood & Cancer | 61 | 1094-1100 | <http://dx.doi.org/10.1002/pbc.24970> |
| Yetisyigit T, Babacan N, Urun Y, Seber ES, Cihan S, Arpaci E, Yildirim N, Aksoy S, Budakoglu B, Zengin N, Oksuzoglu B, Yalcin BC and Alkis N | 2014 | Predictors of outcome in patients with advanced nonseminomatous germ cell testicular tumors. | Asian Pacific Journal of Cancer prevention : APJCP | 15 | 831-835 | <http://dx.doi.org/10.7314/apjcp.2014.15.2.831> |
| Alsultan A, Alharbi M, Al-Dandan S, Bayoumi Y, Alharbi T, Alsudairy R, Alomari A, Aljamaan K, Musleh O, Alharbi Q and Jarrar M | 2015 | High-dose Chemotherapy With Autologous Stem Cell Rescue in Saudi Children Less Than 3 Years of Age With Embryonal Brain Tumors. | Journal of Pediatric Hematology/Oncology | 37 | 204-208 | <http://dx.doi.org/10.1097/MPH.0000000000000301> |
| Fedyanin M, Tryakin A, Bulanov A, Vybarava A, Tjulandina A, Chekini D, Sekhina O, Figurin K, Garin A and Tjulandin S | 2015 | Chemotherapy intensification in patients with advanced seminoma and adverse prognostic factors. | Journal of Cancer Research and Clinical Oncology | 141 | 1259-1264 | <http://dx.doi.org/10.1007/s00432-015-1914-5> |
| Feldman DR, Glezerman I, Patil S, Van Alstine L, Bajorin DF, Fischer P, Hughes A, Sheinfeld J, Bains M, Reich L, Woo K, Giralt S, Bosl GJ and Motzer RJ | 2015 | Phase I/II Trial of Paclitaxel With Ifosfamide Followed by High-Dose Paclitaxel, Ifosfamide, and Carboplatin (TI-TIC) With Autologous Stem Cell Reinfusion for Salvage Treatment of Germ Cell Tumors. | Clinical Genitourinary Cancer | 13 | 453-460 | <http://dx.doi.org/10.1016/j.clgc.2015.05.003> |
| Feldman DR and Powles T | 2015 | Salvage high-dose chemotherapy for germ cell tumors. | Urologic Oncology | 33 | 355-362 | <http://dx.doi.org/10.1016/j.urolonc.2015.01.025> |
| Hou J-Y, Liu H-C, Yeh T-C, Sheu J-C, Chen K-H, Chang C-Y and Liang D-C | 2015 | Treatment Results of Extracranial Malignant Germ Cell Tumor with Regimens of Cisplatin, Vinblastine, Bleomycin or Carboplatin, Etoposide, and Bleomycin with Special Emphasis on the Sites of Vagina and Testis. | Pediatrics and Neonatology | 56 | 301-306 | <http://dx.doi.org/10.1016/j.pedneo.2014.12.003> |
| Lai I-C, Wong T-T, Shiau C-Y, Hu Y-W, Ho DM-T, Chang K-P, Guo W-Y, Chang F-C, Liang M-L, Lee Y-Y, Chen H-H, Yen S-H and Chen Y-W | 2015 | Treatment results and prognostic factors for intracranial nongerminomatous germ cell tumors: single institute experience. | Child's nervous system : ChNS : official Journal of the International Society for Pediatric NeuroSurgery | 31 | 683-691 | <http://dx.doi.org/10.1007/s00381-015-2623-8> |
| Lauritsen J, Kier MGG, Mortensen MS, Bandak M, Gupta R, Holm NV, Agerbaek M and Daugaard G | 2015 | Germ Cell Cancer and Multiple Relapses: Toxicity and Survival. | Journal of Clinical Oncology : official Journal of the American Society of Clinical Oncology | 33 | 3116-3123 | <http://dx.doi.org/10.1200/JCO.2014.60.1310> |
| Madden LM, Ngwube AI, Shenoy S, Druley TE and Hayashi RJ | 2015 | Late toxicity of a novel allogeneic stem cell transplant using single fraction total body irradiation for hematologic malignancies in children. | Journal of Pediatric Hematology/Oncology | 37 | e94-e101 | <http://dx.doi.org/10.1097/MPH.0000000000000272> |
| Mulherin BP, Brames MJ and Einhorn LH | 2015 | Long-term survival with paclitaxel and gemcitabine for germ cell tumors after progression following high-dose chemotherapy with tandem transplant. | American Journal of Clinical Oncology | 38 | 373-376 | <http://dx.doi.org/10.1097/COC.0b013e31829e19e0> |
| Necchi A, Mariani L, Di Nicola M, Lo Vullo S, Nicolai N, Giannatempo P, Raggi D, Fare E, Magni M, Piva L, Matteucci P, Catanzaro M, Biasoni D, Torelli T, Stagni S, Bengala C, Barone C, Schiavetto I, Siena S, Carlo-Stella C, Pizzocaro G, Salvioni R and Gianni AM | 2015 | High-dose sequential chemotherapy (HDS) versus PEB chemotherapy as first-line treatment of patients with poor prognosis germ-cell tumors: mature results of an Italian randomized phase II study. | Annals of Oncology : official Journal of the European Society for Medical Oncology | 26 | 167-172 | <http://dx.doi.org/10.1093/annonc/mdu485> |
| Nieto Y, Tu S-M, Bassett R, Jones RB, Gulbis AM, Tannir N, Kingham A, Ledesma C, Margolin K, Holmberg L, Champlin R and Pagliaro L | 2015 | Bevacizumab/high-dose chemotherapy with autologous stem-cell transplant for poor-risk relapsed or refractory germ-cell tumors. | Annals of Oncology : official Journal of the European Society for Medical Oncology | 26 | 2125-2132 | <http://dx.doi.org/10.1093/annonc/mdv310> |
| Panasiuk A, Nussey S, Veys P, Amrolia P, Rao K, Krawczuk-Rybak M and Leiper A | 2015 | Gonadal function and fertility after stem cell transplantation in childhood: comparison of a reduced intensity conditioning regimen containing melphalan with a myeloablative regimen containing busulfan. | British Journal of Haematology | 170 | 719-726 | <http://dx.doi.org/10.1111/bjh.13497> |
| Servitzoglou M, De Vathaire F, Oberlin O, Patte C and Thomas-Teinturier C | 2015 | Dose-Effect Relationship of Alkylating Agents on Testicular Function in Male Survivors of Childhood Lymphoma. | Pediatric Hematology and Oncology | 32 | 613-623 | <http://dx.doi.org/10.3109/08880018.2015.1085933> |
| Vidal AD, Thalmann GN, Karamitopoulou-Diamantis E, Fey MF and Studer UE | 2015 | Long-term outcome of patients with clinical stage I high-risk nonseminomatous germ-cell tumors 15 years after one adjuvant cycle of bleomycin, etoposide, and cisplatin chemotherapy. | Annals of Oncology : official Journal of the European Society for Medical Oncology | 26 | 374-377 | <http://dx.doi.org/10.1093/annonc/mdu518> |
| Adra N, Albany C, Brames MJ, Case-Eads S, Johnson CS, Liu Z, Fausel CA, Breen T, Hanna NH, Hauke RJ, Picus J and Einhorn LH | 2016 | Phase II study of fosaprepitant + 5HT3 receptor antagonist + dexamethasone in patients with germ cell tumors undergoing 5-day cisplatin-based chemotherapy: a Hoosier Cancer Research Network study. | Supportive care in Cancer : official Journal of the Multinational Association of Supportive Care in Cancer | 24 | 2837-2842 | <http://dx.doi.org/10.1007/s00520-016-3100-y> |
| Awada A, Campone M, Varga A, Aftimos P, Frenel J-S, Bahleda R, Gombos A, Bourbouloux E and Soria J-C | 2016 | An open-label, dose-escalation study to evaluate the safety and pharmacokinetics of CEP-9722 (a PARP-1 and PARP-2 inhibitor) in combination with gemcitabine and cisplatin in patients with advanced solid tumors. | Anti-Cancer Drugs | 27 | 342-348 | <http://dx.doi.org/10.1097/CAD.0000000000000336> |
| Badreldin W, Krell J, Chowdhury S, Harland SJ, Mazhar D, Harding V, Frampton AE, Wilson P, Berney D, Stebbing J and Shamash J | 2016 | The efficacy of irinotecan, paclitaxel, and oxaliplatin (IPO) in relapsed germ cell tumours with high-dose chemotherapy as consolidation: a non-cisplatin-based induction approach. | BJU International | 117 | 418-423 | <http://dx.doi.org/10.1111/bju.13004> |
| Connell BJ, Patel MJ and Tretter CG | 2016 | High-Dose Chemotherapy in a Late Relapse, Platinum-Refractory Nonseminomatous Germ Cell Tumor. | Clinical Genitourinary Cancer | 14 | e441-3 | <http://dx.doi.org/10.1016/j.clgc.2016.03.008> |
| Dechaphunkul A, Sakdejayont S, Sathitruangsak C and Sunpaweravong P | 2016 | Clinical Characteristics and Treatment Outcomes of Patients with Primary Mediastinal Germ Cell Tumors: 10-Years' Experience at a Single Institution with a Bleomycin-Containing Regimen. | Oncology Research and Treatment | 39 | 688-694 | <http://dx.doi.org/10.1159/000452259> |
|  | 2016 | The challenge to one-course carboplatin in seminoma clinical stage 1. | Annals of Oncology : official Journal of the European Society for Medical Oncology | 27 | 1648-1649 | <http://dx.doi.org/10.1093/annonc/mdw187> |
| Egan G, Cervone KA, Philips PC, Belasco JB, Finlay JL and Gardner SL | 2016 | Phase I study of temozolomide in combination with thiotepa and carboplatin with autologous hematopoietic cell rescue in patients with malignant brain tumors with minimal residual Disease. | Bone Marrow Transplantation | 51 | 542-545 | <http://dx.doi.org/10.1038/bmt.2015.313> |
| Ligia Cebotaru C, Zenovia Antone N, Diana Olteanu E, Bejinariu N, Buiga R, Todor N, Ioana Iancu D, Eliade Ciuleanu T and Nagy V | 2016 | A phase II single institution single arm prospective study with paclitaxel, ifosfamide and cisplatin (TIP) as first-line chemotherapy in high-risk germ cell tumor patients with more than ten years follow-up and retrospective correlation with ERCC1, Topois | Journal of B.U.ON. : official Journal of the Balkan Union of Oncology | 21 | 698-708 |  |
| Lopes LF, Macedo CRPD, Aguiar SDS, Barreto JHS, Martins GE, Sonaglio V, Milone M, Lima ER, Almeida MTdA, Lopes PMAA, Watanabe FM, D'Andrea MLM, Pianovski MA, Melaragno R, Vianna SMR, Moreira MES, Bruniera P and de Oliveira CZ | 2016 | Lowered Cisplatin Dose and No Bleomycin in the Treatment of Pediatric Germ Cell Tumors: Results of the GCT-99 Protocol From the Brazilian Germ Cell Pediatric Oncology Cooperative Group. | Journal of Clinical Oncology : official Journal of the American Society of Clinical Oncology | 34 | 603-610 | <http://dx.doi.org/10.1200/JCO.2014.59.1420> |
| Madden LM, Hayashi RJ, Chan KW, Pulsipher MA, Douglas D, Hale GA, Chaudhury S, Haut P, Kasow KA, Gilman AL, Murray LM and Shenoy S | 2016 | Long-Term Follow-Up after Reduced-Intensity Conditioning and Stem Cell Transplantation for Childhood Nonmalignant Disorders. | Biology of blood and marrow transplantation : Journal of the American Society for Blood and Marrow Transplantation | 22 | 1467-1472 | <http://dx.doi.org/10.1016/j.bbmt.2016.04.025> |
| Makino T, Konaka H and Namiki M | 2016 | Clinical Features and Treatment Outcomes in Patients with Extragonadal Germ Cell Tumors: A Single-center Experience. | AntiCancer Research | 36 | 313-317 |  |
| Necchi A, Nicolai N, Alessi A, Miceli R, Giannatempo P, Raggi D, Tana S, Serafini G, Padovano B, Mariani L, Crippa F and Salvioni R | 2016 | Interim (18)F-Fluorodeoxyglucose Positron Emission Tomography for Early Metabolic Assessment of Response to Cisplatin, Etoposide, and Bleomycin Chemotherapy for Metastatic Seminoma: Clinical Value and Future Directions. | Clinical Genitourinary Cancer | 14 | 249-254 | <http://dx.doi.org/10.1016/j.clgc.2015.08.010> |
| Seidel C, Oechsle K, Lorch A, Dieing A, Hentrich M, Hornig M, Grunwald V, Cathomas R, Meiler J, de Wit M and Bokemeyer C | 2016 | Efficacy and safety of gemcitabine, oxaliplatin, and paclitaxel in cisplatin-refractory germ cell cancer in routine care--Registry data from an outcomes research project of the German Testicular Cancer Study Group. | Urologic Oncology | 34 | 167.e21-8 | <http://dx.doi.org/10.1016/j.urolonc.2015.11.007> |
| Shaikh F, Murray MJ, Amatruda JF, Coleman N, Nicholson JC, Hale JP, Pashankar F, Stoneham SJ, Poynter JN, Olson TA, Billmire DF, Stark D, Rodriguez-Galindo C and Frazier AL | 2016 | Paediatric extracranial germ-cell tumours. | The Lancet. Oncology | 17 | e149-e162 | <http://dx.doi.org/10.1016/S1470-2045(15)00545-8> |
| Yonemoto T, Takahashi M, Maru M, Tomioka A, Saito M, Araki Y, Tazaki M, Tsuchiya M, Iwata S, Kamoda H and Ishii T | 2016 | Marriage and fertility in long-term survivors of childhood, adolescent and young adult (AYA) high-grade sarcoma. | International Journal of Clinical Oncology | 21 | 801-807 | <http://dx.doi.org/10.1007/s10147-016-0948-2> |
| Zou T, Yin J, Zheng W, Xiao L, Tan L, Chen J, Wang Y, Li X, Qian C, Cui J, Zhang W, Zhou H and Liu Z | 2016 | Rho GTPases: RAC1 polymorphisms affected platinum-based chemotherapy toxicity in lung cancer patients. | Cancer Chemotherapy and Pharmacology | 78 | 249-258 | <http://dx.doi.org/10.1007/s00280-016-3072-0> |
| Zubizarreta P, Rossa A, Bailez M, Gil S, Rose A and Cacciavillano W | 2016 | Malignant extra-cranial germ cell tumors in children and adolescents. Results following the guidelines of SFOP/SFCE 95 Protocol. | Medicina | 76 | 265-272 |  |
| Agarwala S, Mitra A, Bansal D, Kapoor G, Vora T, Prasad M, Chinnaswamy G, Arora B, Radhakrishnan V, Laskar S, Kaur T, Dhaliwal RS, Rath GK and Bakhshi S | 2017 | Management of Pediatric Malignant Germ Cell Tumors: ICMR Consensus Document. | Indian Journal of Pediatrics | 84 | 465-472 | <http://dx.doi.org/10.1007/s12098-017-2308-2> |
| Chi EA and Schweizer MT | 2017 | Durable Response to Immune Checkpoint Blockade in a Platinum-Refractory Patient With Nonseminomatous Germ Cell Tumor. | Clinical Genitourinary Cancer | 15 | e855-e857 | <http://dx.doi.org/10.1016/j.clgc.2017.04.005> |
| Fischer S, Tandstad T, Wheater M, Porfiri E, Flechon A, Aparicio J, Klingbiel D, Skrbinc B, Basso U, Shamash J, Lorch A, Dieckmann K-P, Cohn-Cedermark G, Stahl O, Chau C, Arriola E, Marti K, Hutton P, Laguerre B, Maroto P, Beyer J and Gillessen S | 2017 | Outcome of Men With Relapse After Adjuvant Carboplatin for Clinical Stage I Seminoma. | Journal of Clinical Oncology : official Journal of the American Society of Clinical Oncology | 35 | 194-200 | <http://dx.doi.org/10.1200/JCO.2016.69.0958> |
| Gonzalez-Billalabeitia E, Sepulveda JM, Maroto P, Aparicio J, Arranz JA, Esteban E, Girones R, Lopez-Brea M, Mendez-Vidal MJ, Pinto A, Sastre J, de Prado DS, Terrasa J, Vazquez S, Powles T, Beyer J, Castellano D and Del Muro XG | 2017 | Consensus Recommendations from the Spanish Germ Cell Cancer Group on the Use of High-dose Chemotherapy in Germ Cell Cancer. | European Urology Focus | 3 | 280-286 | <http://dx.doi.org/10.1016/j.euf.2016.07.002> |
| Grabski DF, Pappo AS, Krasin MJ, Davidoff AM, Rao BN and Fernandez-Pineda I | 2017 | Long-term outcomes of pediatric and adolescent mediastinal germ cell tumors: a single pediatric oncology institutional experience. | Pediatric Surgery International | 33 | 235-244 | <http://dx.doi.org/10.1007/s00383-016-4020-0> |
| Green DM, Zhu L, Wang M, Chemaitilly W, Srivastava D, Kutteh WH, Ke RW, Sklar CA, Pui C-H, Kun LE, Ribeiro RC, Robison LL and Hudson MM | 2017 | Effect of cranial irradiation on sperm concentration of adult survivors of childhood acute lymphoblastic leukemia: a report from the St. Jude Lifetime Cohort Studydagger. | Human Reproduction (Oxford, England) | 32 | 1192-1201 | <http://dx.doi.org/10.1093/humrep/dex082> |
| Huang J, Tan Y, Zhen Z, Lu S, Sun F, Zhu J, Wang J, Liao R and Sun X | 2017 | Role of post-chemotherapy radiation in the management of children and adolescents with primary advanced malignant mediastinal germ cell tumors. | PloS one | 12 | e0183219 | <http://dx.doi.org/10.1371/journal.pone.0183219> |
| Kier MG, Lauritsen J, Mortensen MS, Bandak M, Andersen KK, Hansen MK, Agerbaek M, Holm NV, Dalton SO, Johansen C and Daugaard G | 2017 | Prognostic Factors and Treatment Results After Bleomycin, Etoposide, and Cisplatin in Germ Cell Cancer: A Population-based Study. | European Urology | 71 | 290-298 | <http://dx.doi.org/10.1016/j.eururo.2016.09.015> |
| Necchi A, Lo Vullo S, Giannatempo P, Raggi D, Calareso G, Togliardi E, Crippa F, Pennati M, Zaffaroni N, Perrone F, Busico A, Colecchia M, Nicolai N, Mariani L and Salvioni R | 2017 | Pazopanib in advanced germ cell tumors after chemotherapy failure: results of the open-label, single-arm, phase 2 Pazotest trial. | Annals of Oncology : official Journal of the European Society for Medical Oncology | 28 | 1346-1351 | <http://dx.doi.org/10.1093/annonc/mdx124> |
| Nodomi S, Umeda K, Ueno H, Saida S, Hiramatsu H, Funaki T, Arakawa Y, Mizowaki T, Adachi S and Heike T | 2017 | Efficacy of Ifosfamide-Cisplatin-Etoposide (ICE) Chemotherapy for a CNS Germinoma in a Child With Down Syndrome. | Journal of Pediatric Hematology/Oncology | 39 | e39-e42 | <http://dx.doi.org/10.1097/MPH.0000000000000711> |
| Poganitsch-Korhonen M, Masliukaite I, Nurmio M, Lahteenmaki P, van Wely M, van Pelt AMM, Jahnukainen K and Stukenborg J-B | 2017 | Decreased spermatogonial quantity in prepubertal boys with leukaemia treated with alkylating agents. | Leukemia | 31 | 1460-1463 | <http://dx.doi.org/10.1038/leu.2017.76> |
| Raggi D, Giannatempo P, Miceli R, Fare E, Piva L, Biasoni D, Catanzaro M, Torelli T, Stagni S, Marongiu M, Gianni AM, Nicolai N, Salvioni R and Necchi A | 2017 | Etoposide, Methotrexate, and Dactinomycin Alternating With Cyclophosphamide and Vincristine (EMACO) for Male Patients With HCG-expressing, Chemoresistant Germ Cell Tumors. | American Journal of Clinical Oncology | 40 | 60-65 | <http://dx.doi.org/10.1097/COC.0000000000000113> |
| Shaikh F, Cullen JW, Olson TA, Pashankar F, Malogolowkin MH, Amatruda JF, Villaluna D, Krailo M, Billmire DF, Rescorla FJ, Egler RA, Dicken BJ, Ross JH, Schlatter M, Rodriguez-Galindo C and Frazier AL | 2017 | Reduced and Compressed Cisplatin-Based Chemotherapy in Children and Adolescents With Intermediate-Risk Extracranial Malignant Germ Cell Tumors: A Report From the Children's Oncology Group. | Journal of Clinical Oncology : official Journal of the American Society of Clinical Oncology | 35 | 1203-1210 | <http://dx.doi.org/10.1200/JCO.2016.67.6544> |
| Sudour-Bonnange H, Faure-Conter C, Martelli H, Hameury F, Fresneau B, Orbach D and Verite C | 2017 | Primary mediastinal and retroperitoneal malignant germ cell tumors in children and adolescents: Results of the TGM95 trial, a study of the French Society of Pediatric Oncology (Societe Francaise des Cancers de l'Enfant). | Pediatric Blood & Cancer | 64 |  | <http://dx.doi.org/10.1002/pbc.26494> |
| Frazier AL, Stoneham S, Rodriguez-Galindo C, Dang H, Xia C, Olson TA, Murray MJ, Amatruda JF, Shaikh F, Pashankar F, Billmire D, Krailo M, Stark D, Brougham MFH, Nicholson JC and Hale JP | 2018 | Comparison of carboplatin versus cisplatin in the treatment of paediatric extracranial malignant germ cell tumours: A report of the Malignant Germ Cell International Consortium. | European Journal of Cancer (Oxford, England : 1990) | 98 | 30-37 | <http://dx.doi.org/10.1016/j.ejca.2018.03.004> |
| Gray HJ, Bell-McGuinn K, Fleming GF, Cristea M, Xiong H, Sullivan D, Luo Y, McKee MD, Munasinghe W and Martin LP | 2018 | Phase I combination study of the PARP inhibitor veliparib plus carboplatin and gemcitabine in patients with advanced ovarian cancer and other solid malignancies. | Gynecologic Oncology | 148 | 507-514 | <http://dx.doi.org/10.1016/j.ygyno.2017.12.029> |
| Hsieh T-H, Liu Y-R, Chang T-Y, Liang M-L, Chen H-H, Wang H-W, Yen Y and Wong T-T | 2018 | Global DNA methylation analysis reveals miR-214-3p contributes to cisplatin resistance in pediatric intracranial nongerminomatous malignant germ cell tumors. | Neuro-Oncology | 20 | 519-530 | <http://dx.doi.org/10.1093/neuonc/nox186> |
| Lawrence NJ, Chan H, Toner G, Stockler MR, Martin A, Yip S, Wong N, Yeung A, Mazhar D, Pashankar F, Frazier L, McDermott R, Walker R, Tan H, Davis ID and Grimison P | 2018 | Protocol for the P3BEP trial (ANZUP 1302): an international randomised phase 3 trial of accelerated versus standard BEP chemotherapy for adult and paediatric male and female patients with intermediate and poor-risk metastatic germ cell tumours. | BMC Cancer | 18 | 854 | <http://dx.doi.org/10.1186/s12885-018-4745-3> |
| Osorio DS, Dunkel IJ, Cervone KA, Goyal RK, Steve Lo KM, Finlay JL and Gardner SL | 2018 | Tandem thiotepa with autologous hematopoietic cell rescue in patients with recurrent, refractory, or poor prognosis solid tumor malignancies. | Pediatric Blood & Cancer | 65 |  | <http://dx.doi.org/10.1002/pbc.26776> |
| Pashankar F, Frazier AL, Krailo M, Xia C, Pappo AS, Malogolowkin M, Olson TA and Rodriguez-Galindo C | 2018 | Treatment of refractory germ cell tumors in children with paclitaxel, ifosfamide, and carboplatin: A report from the Children's Oncology Group AGCT0521 study. | Pediatric Blood & Cancer | 65 | e27111 | <http://dx.doi.org/10.1002/pbc.27111> |
| Stukenborg J-B, Alves-Lopes JP, Kurek M, Albalushi H, Reda A, Keros V, Tohonen V, Bjarnason R, Romerius P, Sundin M, Noren Nystrom U, Langenskiold C, Vogt H, Henningsohn L, Mitchell RT, Soder O, Petersen C and Jahnukainen K | 2018 | Spermatogonial quantity in human prepubertal testicular tissue collected for fertility preservation prior to potentially sterilizing therapy. | Human Reproduction (Oxford, England) | 33 | 1677-1683 | <http://dx.doi.org/10.1093/humrep/dey240> |
| Terenziani M, De Pasquale MD, Bisogno G, Biasoni D, Boldrini R, Collini P, Conte M, Dall'Igna P, Inserra A, Melchionda F, Siracusa F, Spreafico F, Barretta F and D'Angelo P | 2018 | Malignant testicular germ cell tumors in children and adolescents: The AIEOP (Associazione Italiana Ematologia Oncologia Pediatrica) protocol. | Urologic Oncology | 36 | 502.e7-502.e13 | <http://dx.doi.org/10.1016/j.urolonc.2018.07.001> |
| Tryakin A, Fedyanin M, Bulanov A, Kashia S, Kurmukov I, Matveev V, Fainstein I, Gordeeva O, Zakharova T and Tjulandin S | 2018 | Dose-reduced first cycle of chemotherapy for prevention of life-threatening acute complications in nonseminomatous germ cell tumor patients with ultra high tumor markers and/or poor performance status. | Journal of Cancer Research and Clinical Oncology | 144 | 1817-1823 | <http://dx.doi.org/10.1007/s00432-018-2695-4> |
|  | 2019 | Controversies in the management of stage I seminoma: adjuvant carboplatin revisited. | Clinical & Translational Oncology : official publication of the Federation of Spanish Oncology Societies and of the National Cancer Institute of Mexico | 21 | 246-247 | <http://dx.doi.org/10.1007/s12094-018-1917-1> |
| Batra A, Ernst S, Potvin K, Fernandes R, Power N, Vanhie J and Winquist E | 2019 | Early experience with chemotherapy intensification for poor prognosis metastatic germ cell cancer and unfavorable tumor marker decline. | Canadian Urological Association Journal = Journal de l'Association des urologues du Canada |  |  | <http://dx.doi.org/10.5489/cuaj.5802> |
| Fenner M, Oing C, Dieing A, Gauler T, Oechsle K, Lorch A, Hentrich M, Kopp H-G, Bokemeyer C and Honecker F | 2019 | Everolimus in patients with multiply relapsed or cisplatin refractory germ cell tumors: results of a phase II, single-arm, open-label multicenter trial (RADIT) of the German Testicular Cancer Study Group. | Journal of Cancer Research and Clinical Oncology | 145 | 717-723 | <http://dx.doi.org/10.1007/s00432-018-2752-z> |
| Hamid AA, Markt SC, Vicier C, McDermott K, Richardson P, Ho VT and Sweeney CJ | 2019 | Autologous Stem-Cell Transplantation Outcomes for Relapsed Metastatic Germ-Cell Tumors in the Modern Era. | Clinical Genitourinary Cancer | 17 | 58-64.e1 | <http://dx.doi.org/10.1016/j.clgc.2018.09.009> |
