# Supplementary material for: Impacts of platinum-based chemotherapy on subsequent testicular function and fertility in boys with cancer
Source: Hum Reprod Update. 2020 Sep 16;26(6):874–85. doi: 10.1093/humupd/dmaa041 (PMC7600277; doi:10.1093/humupd/dmaa041)
Supplement: dmaa041_Supplementary_Data [file dmaa041_supplementary_data.zip › dmaa041-suppl_data/Supplementary_Table_SIII final.docx]

**Supplementary Table SIII** The 16 publications subjected to detailed full-text screening and excluded with reasons for exclusion.

| **Author** | **Year** | **Title** | **Journal** | **Volume** | **Pages** | **DOI** | **Exclusion Reason** |
| --- | --- | --- | --- | --- | --- | --- | --- |
| Castaneda VL, Parmley RT, Geiser CF, Saldivar VA, Mullins JK and Marlin AE | 1990 | Postoperative chemotherapy for primary intracranial germ cell tumor. | Medical and Pediatric Oncology | 18 | 299-303 | <https://doi.org/10.1002/mpo.2950180409> | No post-chemotherapy fertility outcomes |
| Siimes MA, Elomaa I and Koskimies A | 1990 | Testicular function after chemotherapy for osteosarcoma. | European Journal of Cancer (Oxford, England : 1990) | 26 | 973-975 | <https://doi.org/10.1016/0277-5379(90)90623-2> | Adult participants |
| Vijayaraghavan S, Brock C, Monson JP, Snodgrass GJ, King TT, Gallagher C and Oliver RT | 1993 | Does the rapid response to cisplatin-based chemotherapy justify its use as primary treatment for intracranial germ-cell tumours? | The Quarterly Journal of Medicine | 86 | 801-810 | <https://doi.org/10.1093/oxfordjournals.qjmed.a068763> | No fertility outcomes |
| Herrmann HD, Westphal M, Winkler K, Laas RW and Schulte FJ | 1994 | Treatment of nongerminomatous germ-cell tumors of the pineal region. | Neurosurgery | 34 | 524-9 | <https://doi.org/10.1227/00006123-199403000-00021> | No fertility outcomes |
| Buckner JC, Peethambaram PP, Smithson WA, Groover RV, Schomberg PJ, Kimmel DW, Raffel C, O'Fallon JR, Neglia J and Shaw EG | 1999 | Phase II trial of primary chemotherapy followed by reduced-dose radiation for CNS germ cell tumors. | Journal of Clinical Oncology : official journal of the American Society of Clinical Oncology | 17 | 933-940 | <https://doi.org/10.1200/JCO.1999.17.3.933> | No post-chemotherapy fertility outcomes |
| Bacci G, Ferrari S, Bertoni F, Ruggieri P, Picci P, Longhi A, Casadei R, Fabbri N, Forni C, Versari M and Campanacci M | 2000 | Long-term outcome for patients with nonmetastatic osteosarcoma of the extremity treated at the istituto ortopedico rizzoli according to the istituto ortopedico rizzoli/osteosarcoma-2 protocol: an updated report. | Journal of Clinical Oncology : official journal of the American Society of Clinical Oncology | 18 | 4016-4027 | <https://doi.org/10.1200/JCO.2000.18.24.4016> | No fertility outcomes for pre-pubertal patients |
| Janmohamed S, Grossman AB, Metcalfe K, Lowe DG, Wood DF, Chew SL, Monson JP, Besser GM and Plowman PN | 2002 | Suprasellar germ cell tumours: specific problems and the evolution of optimal management with a combined chemoradiotherapy regimen. | Clinical Endocrinology | 57 | 487-500 | <https://doi.org/10.1046/j.1365-2265.2002.01620.x> | Adult male participants |
| Gaffan J, Holden L, Newlands ES, Short D, Fuller S, Begent RHJ, Rustin GJS and Seckl MJ | 2003 | Infertility rates following POMB/ACE chemotherapy for male and female germ cell tumours - a retrospective long-term follow-up study. | British Journal of Cancer | 89 | 1849-1854 | <https://doi.org/10.1038/sj.bjc.6601383> | Adult participants |
| Kochi M, Itoyama Y, Shiraishi S, Kitamura I, Marubayashi T and Ushio Y | 2003 | Successful treatment of intracranial nongerminomatous malignant germ cell tumors by administering neoadjuvant chemotherapy and radiotherapy before excision of residual tumors. | Journal of Neurosurgery | 99 | 106-114 | <https://doi.org/10.3171/jns.2003.99.1.0106> | No fertility outcomes |
| Kellie SJ, Boyce H, Dunkel IJ, Diez B, Rosenblum M, Brualdi L and Finlay JL | 2004 | Primary chemotherapy for intracranial nongerminomatous germ cell tumors: results of the second international CNS germ cell study group protocol. | Journal of Clinical Oncology : official journal of the American Society of Clinical Oncology | 22 | 846-853 | <https://doi.org/10.1200/JCO.2004.07.006> | No post-chemotherapy fertility outcomes |
| Modak S, Gardner S, Dunkel IJ, Balmaceda C, Rosenblum MK, Miller DC, Halpern S and Finlay JL | 2004 | Thiotepa-based high-dose chemotherapy with autologous stem-cell rescue in patients with recurrent or progressive CNS germ cell tumors. | Journal of Clinical Oncology : official journal of the American Society of Clinical Oncology | 22 | 1934-1943 | <https://doi.org/10.1200/JCO.2004.11.053> | No fertility outcomes |
| Hsiao W, Stahl PJ, Osterberg EC, Nejat E, Palermo GD, Rosenwaks Z and Schlegel PN | 2011 | Successful treatment of postchemotherapy azoospermia with microsurgical testicular sperm extraction: the Weill Cornell experience. | Journal of Clinical Oncology : official journal of the American Society of Clinical Oncology | 29 | 1607-1611 | <https://doi.org/10.1200/JCO.2010.33.7808> | Adult participants |
| Goldman S, Bouffet E, Fisher PG, Allen JC, Robertson PL, Chuba PJ, Donahue B, Kretschmar CS, Zhou T, Buxton AB and Pollack IF | 2015 | Phase II Trial Assessing the Ability of Neoadjuvant Chemotherapy With or Without Second-Look Surgery to Eliminate Measurable Disease for Nongerminomatous Germ Cell Tumors: A Children's Oncology Group Study. | Journal of Clinical Oncology : official journal of the American Society of Clinical Oncology | 33 | 2464-2471 | <https://doi.org/10.1200/JCO.2014.59.5132> | No fertility outcomes |
| Gunn HM, Rinne I, Emilsson H, Gabriel M, Maguire AM and Steinbeck KS | 2016 | Primary Gonadal Insufficiency in Male and Female Childhood Cancer Survivors in a Long-Term Follow-Up Clinic. | Journal of Adolescent and young Adult Oncology | 5 | 344-350 | <https://doi.org/10.1089/jayao.2016.0007> | No use of platinum agents reported |
| Biswas A, Julka PK, Bakhshi S, Singh M and Rath GK | 2017 | Treatment Outcome in Patients with Primary Central Nervous System Germ Cell Tumour: Clinical Experience from a Regional Cancer Centre in North India. | Pediatric Neurosurgery | 52 | 240-249 | <https://doi.org/10.1159/000474946> | No chemotherapy-related fertility outcomes |
| Calaminus G, Frappaz D, Kortmann RD, Krefeld B, Saran F, Pietsch T, Vasiljevic A, Garre ML, Ricardi U, Mann JR, Gobel U, Alapetite C, Murray MJ and Nicholson JC | 2017 | Outcome of patients with intracranial non-germinomatous germ cell tumors-lessons from the SIOP-CNS-GCT-96 trial. | Neuro-oncology | 19 | 1661-1672 | <https://doi.org/10.1093/neuonc/nox122> | No fertility outcomes |
