# Supplementary material for: Impacts of platinum-based chemotherapy on subsequent testicular function and fertility in boys with cancer
Source: Hum Reprod Update. 2020 Sep 16;26(6):874–85. doi: 10.1093/humupd/dmaa041 (PMC7600277; doi:10.1093/humupd/dmaa041)
Supplement: dmaa041_Supplementary_Data [file dmaa041_supplementary_data.zip › dmaa041-suppl_data/Supplementary_Table_SIV final.docx]

| **Supplementary Table SIV** Assessment of risk of bias for all included studies. | | | |  |
| --- | --- | --- | --- | --- |
| **COHORT STUDIES WITH SUB -ANALYSIS FOR PLATINUM-EXPOSURE** | | | | |
|  |  |  |  | |
| **Ridola 2009** | |  |  | |
| **Category** | **Sub-category** | **Risk** | **Details** | |
| ***Confounders*** | Pre-existing | Serious | Variation in pubertal stage | |
|  | Intervention | Serious | Multiple treatment regimens including alkylators | |
|  | Post-intervention | Serious | Higher dose for relapse | |
| ***Selection*** | Loss to follow-up | Medium | Loss to follow-up | |
|  | Selection post-intervention | Serious | Excluded due to having fathered children, retrospective study | |
| ***Information*** | Recall bias - intervention | Medium | Retrospective case-notes | |
|  | Detection bias - outcome | Medium | Lack of defined outcome, variable timing of outcome measure | |
| ***Reporting*** | Selection of outcome measure | Medium | Platinum outcome reported for testicular function as 'no impact’ | |
|  |  |  |  | |
| **Tromp 2011** | |  |  | |
| **Category** | **Sub-category** | **Risk** | **Details** | |
| ***Confounders*** | Pre-existing | Serious | Variation in pubertal stage | |
|  | Intervention | Serious | Multiple treatment regimens including alkylators | |
|  | Post-intervention | Medium | Deaths post-intervention | |
| ***Selection*** | Loss to follow-up | Medium | Loss to follow-up, deaths post-intervention | |
|  | Selection post-intervention | Medium | Retrospective study | |
| ***Information*** | Recall bias - intervention | Medium | Retrospective cancer registry | |
|  | Detection bias - outcome | Medium | Exclude patients outwith region | |
| ***Reporting*** | Selection of outcome measure | Medium | Univariate/multivariate analysis of platinum (excluding other agents) | |
|  |  |  |  | |
| **Reinmuth 2013** | |  |  | |
| **Category** | **Sub-category** | **Risk** | **Details** | |
| ***Confounders*** | Pre-existing | Serious | Variation in pubertal stage | |
|  | Intervention | Serious | Variation in diagnoses and treatment regimens | |
|  | Post-intervention | Medium | Relapse and post-intervention treatment not described | |
| ***Selection*** | Loss to follow-up | Medium | Loss to follow-up, deaths post-intervention | |
|  | Selection post-intervention | Medium | Retrospective study | |
| ***Information*** | Recall bias - intervention | Medium | Retrospective cancer registry, questionnaire | |
|  | Detection bias - outcome | Medium | Lack of clearly defined outcome | |
| ***Reporting*** | Selection of outcome measure | Medium | Univariate analysis for cisplatin not including puberty | |
|  |  |  |  | |
| **Green, Nolan et al 2014** | |  |  | |
| **Category** | **Sub-category** | **Risk** | **Details** | |
| ***Confounders*** | Pre-existing | Serious | Variation in pubertal stage | |
|  | Intervention | Serious | Variation in diagnoses and treatment regimens | |
|  | Post-intervention | Medium | Relapse and post-intervention treatment not described | |
| ***Selection*** | Loss to follow-up | Serious | Loss to follow-up | |
|  | Selection post-intervention | Medium | Retrospective study | |
| ***Information*** | Recall bias - intervention | Medium | Retrospective study using cancer registry | |
|  | Detection bias - outcome | Medium | Defined outcome - pregnancy | |
| ***Reporting*** | Selection of outcome measure | Medium | Cisplatin data reported | |
| **Green, Liu et al 2104** | |  |  | |
| **Category** | **Sub-category** | **Risk** | **Details** | |
| ***Confounders*** | Pre-existing | Serious | Variation in pubertal stage | |
|  | Intervention | Serious | Variation in diagnoses and treatment regimens | |
|  | Post-intervention | Medium | Relapse and post-intervention treatment not described | |
| ***Selection*** | Loss to follow-up | Serious | Loss to follow-up | |
|  | Selection post-intervention | Serious | Men who had fathered child less likely to provide semen sample | |
| ***Information*** | Recall bias - intervention | Medium | Retrospective study | |
|  | Detection bias - outcome | Low | Clearly defined outcome - semen analysis | |
| ***Reporting*** | Selection of outcome measure | Serious | Outcome measures reported for CED only | |
|  |  |  |  | |
| **Wasilewski-Masker 2014** | |  |  | |
| **Category** | **Sub-category** | **Risk** | **Details** | |
| ***Confounders*** | Pre-existing | Serious | Variation in pubertal stage | |
|  | Intervention | Serious | Variation in diagnoses and treatment regimens | |
|  | Post-intervention | Medium | Relapse and post-intervention treatment not described | |
| ***Selection*** | Loss to follow-up | Serious | Loss to follow-up | |
|  | Selection post-intervention | Serious | Restricted to survivors who had tried to achieve pregnancy | |
| ***Information*** | Recall bias - intervention | Medium | Retrospective cancer registry, questionnaire | |
|  | Detection bias - outcome | Low | Clearly defined outcome | |
| ***Reporting*** | Selection of outcome measure | Medium | Platinum not included in multivariate model | |
|  |  |  |  | |
| **Chow 2016** | |  |  | |
| **Category** | **Sub-category** | **Risk** | **Details** | |
| ***Confounders*** | Pre-existing | Serious | Variation in pubertal stage | |
|  | Intervention | Serious | Variation in diagnoses and treatment regimens | |
|  | Post-intervention | Medium | Relapse and post-intervention treatment not described | |
| ***Selection*** | Loss to follow-up | Serious | Loss to follow-up | |
|  | Selection post-intervention | Medium | Restricted inclusion to specific age-groups | |
| ***Information*** | Recall bias - intervention | Medium | Retrospective cancer registry | |
|  | Detection bias - outcome | Low | Clearly defined outcome | |
| ***Reporting*** | Selection of outcome measure | Medium | Includes imputed data | |
|  |  |  |  | |
| **Utrainen 2019** | |  |  | |
| **Category** | **Sub-category** | **Risk** | **Details** | |
| ***Confounders*** | Pre-existing | Low | All prepubertal with neuroblastoma | |
|  | Intervention | Serious | Variation in treatment regimens | |
|  | Post-intervention | Medium | Relapse and post-intervention treatment not described | |
| ***Selection*** | Loss to follow-up | Medium | Loss to follow-up | |
|  | Selection post-intervention | Low | Follow-up reported | |
| ***Information*** | Recall bias - intervention | Medium | Retrospective | |
|  | Detection bias - outcome | Low | Clearly defined outcome | |
| ***Reporting*** | Selection of outcome measure | Medium | Multiple outcome measures, cisplatin analysis included | |
|  |  |  |  | |
|  |  |  |  | |
| **COHORT STUDIES WITHOUT SUB -ANALYSIS FOR PLATINUM-EXPOSURE** | | | | |
|  |  |  |  | |
| **Flamant 1984** | |  |  | |
| **Category** | **Sub-category** | **Risk** | **Details** | |
| ***Confounders*** | Pre-existing | Serious | Includes multiple diagnoses | |
|  | Intervention | Medium | Same treatment for all subjects but includes alkylators | |
|  | Post-intervention | Serious | Includes abdominal radiotherapy | |
| ***Selection*** | Loss to follow-up | Serious | Deaths post-intervention | |
|  | Selection post-intervention | Low | Prospective study | |
| ***Information*** | Recall bias - intervention | Critical | No data reported for most patients | |
|  | Detection bias - outcome | Critical | Lack of defined outcome measures | |
| ***Reporting*** | Selection of outcome measure | Critical | Reporting on one patient with 'sterility' | |
|  |  |  |  | |
| **Wallace 1989** | |  |  | |
| **Category** | **Sub-category** | **Risk** | **Details** | |
| ***Confounders*** | Pre-existing | Medium | Variation in pubertal stage | |
|  | Intervention | Medium | Variable treatments | |
|  | Post-intervention | Critical | Variation in pubertal stage (short-term follow-up) | |
| ***Selection*** | Loss to follow-up | Medium | No loss to follow-up | |
|  | Selection post-intervention | Medium | Retrospective study | |
| ***Information*** | Recall bias - intervention | Medium | Retrospective study | |
|  | Detection bias - outcome | Critical | Defined outcome, short duration of follow-up | |
| ***Reporting*** | Selection of outcome measure | Serious | Short-term follow-up | |
|  |  |  |  | |
| **Kiltie 1995** | |  |  | |
| **Category** | **Sub-category** | **Risk** | **Details** | |
| ***Confounders*** | Pre-existing | Serious | Evolving treatments over time | |
|  | Intervention | Serious | Multiple treatment regimens including CSI | |
|  | Post-intervention | Serious | Relapses post-intervention | |
| ***Selection*** | Loss to follow-up | Serious | Deaths post-intervention | |
|  | Selection post-intervention | Medium | Retrospective study | |
| ***Information*** | Recall bias - intervention | Medium | Retrospective case-notes | |
|  | Detection bias - outcome | Critical | Lack of defined outcome measures | |
| ***Reporting*** | Selection of outcome measure | Serious | Multiple time-points | |
|  |  |  |  | |
| **Muller 1996** | |  |  | |
| **Category** | **Sub-category** | **Risk** | **Details** | |
| ***Confounders*** | Pre-existing | Serious | Variation in pubertal stage | |
|  | Intervention | Serious | Multiple treatment regimens including testicular irradiation | |
|  | Post-intervention | Serious | Relapses post-intervention | |
| ***Selection*** | Loss to follow-up | Medium | No loss to follow-up | |
|  | Selection post-intervention | Medium | Retrospective study | |
| ***Information*** | Recall bias - intervention | Medium | Retrospective case-notes | |
|  | Detection bias - outcome | Medium | Defined outcome, variable timing of outcome measure | |
| ***Reporting*** | Selection of outcome measure | Serious | Only accounts for all non-alkylators | |
|  |  |  |  | |
| **Hale 1999** | |  |  | |
| **Category** | **Sub-category** | **Risk** | **Details** | |
| ***Confounders*** | Pre-existing | Serious | Variation in pubertal stage | |
|  | Intervention | Serious | Multiple treatment regimens including alkylators | |
|  | Post-intervention | Critical | Cross-over of treatment regimens for partial response | |
| ***Selection*** | Loss to follow-up | Medium | Loss to follow-up | |
|  | Selection post-intervention | Medium | Retrospective study | |
| ***Information*** | Recall bias - intervention | Medium | Questionnaire and follow-up telephone | |
|  | Detection bias - outcome | Critical | Information on males not frequently evaluated | |
| ***Reporting*** | Selection of outcome measure | Serious | Multiple methods not clearly defined | |
|  |  |  |  | |
| **Relander 2000** | |  |  | |
| **Category** | **Sub-category** | **Risk** | **Details** | |
| ***Confounders*** | Pre-existing | Serious | Variation in diagnosis and pubertal stage | |
|  | Intervention | Serious | Multiple regimens including alkylators and testicular irradiation | |
|  | Post-intervention | Medium | Relapse post-intervention | |
| ***Selection*** | Loss to follow-up | Low | No loss to follow up | |
|  | Selection post-intervention | Medium | Retrospective study | |
| ***Information*** | Recall bias - intervention | Medium | Retrospective case-notes, questionnaire and interview | |
|  | Detection bias - outcome | Medium | Defined outcome, variable timing of outcome measure | |
| ***Reporting*** | Selection of outcome measure | Serious | Reporting of sub-groups | |
|  |  |  |  | |
| **Longhi 2003** | |  |  | |
| **Category** | **Sub-category** | **Risk** | **Details** | |
| ***Confounders*** | Pre-existing | Serious | Variation in pubertal stage | |
|  | Intervention | Serious | Multiple treatment regimens including alkylators | |
|  | Post-intervention | Serious | Schedule change for poor responders | |
| ***Selection*** | Loss to follow-up | Low | No loss to follow up | |
|  | Selection post-intervention | Medium | Retrospective study | |
| ***Information*** | Recall bias - intervention | Medium | Retrospective case-notes, questionnaire and interview | |
|  | Detection bias - outcome | Medium | Defined outcome, variable timing of outcome measure | |
| ***Reporting*** | Selection of outcome measure | Critical | Multiple timepoints, only 1 prepubertal patient reported | |
|  |  |  |  | |
| **Romerius 2011** | |  |  | |
| **Category** | **Sub-category** | **Risk** | **Details** | |
| ***Confounders*** | Pre-existing | Serious | Variation in pubertal stage | |
|  | Intervention | Serious | Multiple treatment regimens including alkylators | |
|  | Post-intervention | Medium | No treatment for last 4 years but post-intervention not reported | |
| ***Selection*** | Loss to follow-up | Medium | Loss to follow-up | |
|  | Selection post-intervention | Medium | Exclusion of men on testsoterone | |
| ***Information*** | Recall bias - intervention | Medium | Retrospective case-notes | |
|  | Detection bias - outcome | Medium | Defined outcome, variable timing of outcome measure | |
| ***Reporting*** | Selection of outcome measure | Serious | Outcome measures reported for combination alkylating/platinum agents | |
|  |  |  |  | |
| **Odagiri 2012** | |  |  | |
| **Category** | **Sub-category** | **Risk** | **Details** | |
| ***Confounders*** | Pre-existing | Serious | Variation in pubertal stage | |
|  | Intervention | Serious | Variation in treatments regimens over time, cranial irradiation | |
|  | Post-intervention | Low | No relapse and no deaths | |
| ***Selection*** | Loss to follow-up | Low | All eligible patients included | |
|  | Selection post-intervention | Medium | Retrospective study | |
| ***Information*** | Recall bias - intervention | Medium | Retrospective case-notes | |
|  | Detection bias - outcome | Serious | Outcome related to fertility or pituitary damage | |
| ***Reporting*** | Selection of outcome measure | Serious | Outcome measure not fully reported | |
|  |  |  |  | |
| **Brignardello 2016** | |  |  | |
| **Category** | **Sub-category** | **Risk** | **Details** | |
| ***Confounders*** | Pre-existing | Serious | Variation in pubertal stage | |
|  | Intervention | Serious | Variation in diagnoses and treatment regimens | |
|  | Post-intervention | Medium | Relapse and post-intervention treatment not described | |
| ***Selection*** | Loss to follow-up | Medium | Loss to follow-up | |
|  | Selection post-intervention | Medium | Retrospective study | |
| ***Information*** | Recall bias - intervention | Medium | Retrospective case-notes | |
|  | Detection bias - outcome | Medium | Multiple outcome measures | |
| ***Reporting*** | Selection of outcome measure | Serious | Multiple scales used to define impaired spermatogenesis | |
|  |  |  |  | |
| **Isaksson 2018** | |  |  | |
| **Category** | **Sub-category** | **Risk** | **Details** | |
| ***Confounders*** | Pre-existing | Serious | Variation in pubertal stage | |
|  | Intervention | Serious | Variation in diagnoses and treatment regimens | |
|  | Post-intervention | Medium | Relapse and post-intervention treatment not described | |
| ***Selection*** | Loss to follow-up | Serious | Loss to follow-up | |
|  | Selection post-intervention | Medium | Retrospective study, exclusion for androgen replacement therapy | |
| ***Information*** | Recall bias - intervention | Medium | Retrospective | |
|  | Detection bias - outcome | Medium | Clearly defined outcome, may include pituitary effects | |
| ***Reporting*** | Selection of outcome measure | Serious | Outcome measures not reported for platinum agents | |
|  |  |  |  | |
|  |  |  |  | |
| **SINGLE CASE REPORTS** | | | | |
|  |  |  |  | |
| **Chemaitilly 2016** | |  |  | |
| **Category** | **Sub-category** | **Risk** | **Details** | |
| ***Confounders*** | Pre-existing | Medium | Prepubertal | |
|  | Intervention | Critical | Multiple agent chemotherapy | |
|  | Post-intervention | Medium | VP shunt revision | |
| ***Selection*** | Loss to follow-up | n/a | Single case report | |
|  | Selection post-intervention | n/a | Single case report | |
| ***Information*** | Recall bias - intervention | Medium | Retrospective | |
|  | Detection bias - outcome | Critical | Lack defined outcome | |
| ***Reporting*** | Selection of outcome measure | Critical | Case report | |

n/a – not applicable.
