# Supplementary material for: Impacts of platinum-based chemotherapy on subsequent testicular function and fertility in boys with cancer
Source: Hum Reprod Update. 2020 Sep 16;26(6):874–85. doi: 10.1093/humupd/dmaa041 (PMC7600277; doi:10.1093/humupd/dmaa041)
Supplement: dmaa041_Supplementary_Data [file dmaa041_supplementary_data.zip › dmaa041-suppl_data/Supplementary_Table_SV final.docx]

**Supplementary Table SV** Detailed summary of full text publications included in the systematic review.

|  | | | | | |
| --- | --- | --- | --- | --- | --- |
| **Study design** | **Participants** | **Treatment** | **Fertility outcomes** | **Control group** | **Additional comments** |
| Flamant F, Schwartz L, Delons E, Caillaud JM, Hartmann O, Lemerle J (1984). Nonseminomatous malignant germ cell tumors in children. Multidrug therapy in Stages III and IV. *Cancer* 54, 1687-1691. | | | | | |
| Two-centre cohort study | 35 childhood cancer survivors (13 male, 22 female)  Age: 1-14 years  Diagnosis: Nonseminomatous malignant germ cell tumour | Surgery: Orchiectomy followed by lymphadenectomy for testicular primaries (n=4)  Chemotherapy: Actinomycin D, cyclophosphamide, vincristine, doxorubicin, bleomycin, cisplatin | One case of sterility attributed to whole abdominal irradiation | No |  |
| Wallace WH, Shalet SM, Crowne EC, Morris-Jones PH, Gattamaneni HR, Price DA (1989). Gonadal dysfunction due to cis-platinum. *Med Pediatr Oncol* 5, 409-13. | | | | | |
| Cohort study | 15 patients (8 male, 7 female)  Age at diagnosis (Male): 7.3-14.6 years  Diagnosis: Osteosarcoma | Chemotherapy:  Protocol A: Adriamycin, cisplatin  Protocol B: Methotrexate, Adriamycin, cisplatin  Protocol C: Methotrexate, vincristine, bleomycin, cyclophosphamide, actinomycin D, Adriamycin, cisplatin  Protocol D: cisplatin, ifosfamide, Adriamycin | Semen analysis:  1 patient developed oligospermia (3.3x10^5^/ml)  Gonadotrophins and Testicular volume:  1 patient has significantly elevated Follicle Stimulating Hormone (FSH; 11 IU/L) and Luteinising Hormone (LH; 12 IU/L), normal testosterone (15 nmol/L) and small testes (11.5 ml)  1 patient had decreasing testicular volume (12 ml to 10 ml) over 1.2 years | No | Patients who were prepubertal at time of assessment (n=3) showed normal prepubertal gonadotrophins and testosterone |
| Kiltie AE & Gattamaneni HR (1995). Survival and quality of life of paediatric intracranial germ cell tumour patients treated at the Christie Hospital, 1972-1993. *Medical and pediatric oncology* 25:450-456. | | | | | |
| Single-centre cohort study | 25 childhood cancer patients (18 male, 7 female, 17 survivors)  Age: Median 10 (3-15) years  Diagnosis: Intracranial germ cell tumour | Vincristine: 7 (28%)  Etoposide: 8 (32%)  Cyclophosphamide:  1 (4%)  Actinomycin D: 1 (4%)  Cisplatin: 1 (4%)  Carboplatin: 5 (20%)  Bleomycin: 5 (20%)  Platinum-based chemotherapy at relapse: 2  Non-platinum based chemotherapy at relapse: 1 | Requiring sex hormone replacement: 4 (23.5%)  Partial FSH deficiency: 1 (5.9%) | No | All but two survivors received craniospinal irradiation. While two survivors presented with precocious puberty, none developed precocious puberty following radiotherapy |
| Muller HL, Klinkhammer-Schalke M, Seelbach-Gobel B, Hartmann AA, Kuhl J (1996). Gonadal function of young adults after therapy of malignancies during childhood or adolescence. *European journal of pediatrics* 155, 763-769. | | | | | |
| Single-centre cohort study | 54 long-term childhood cancer survivors (33 male, 21 female)  Age at diagnosis (Male): Median 14.3 (7.8-17.3) years  Age at follow-up (Male): Median 23.5 (22.1-34.4) years  Diagnoses (Male):  Ewing sarcoma (n=2), Rhabdomyosarcoma (n=1), Osteosarcoma (n=5), Synovial sarcoma (n=1), Hodgkin’s disease (n=13), Neuroblastoma (n=6), Medulloblastoma (n=3), Acute Lymphoblastic Leukaemia (n=2) | Radiotherapy (Male): n=25  Cumulative gonadal dose: Median 5 (2-50/2400^a^) cGy  Chemotherapy cumulative doses (Male): n=29  Cyclophosphamide (n=21): Median 4 (1.5-26) g/m^2^  Ifosfamide (n=3): Median 63 (12-72) g/m^2^  CCNU (n=1): 0.8 g/m^2^  Procarbazine (n=10): Median 6.5 (3-29.2) g/m^2^ | Semen analysis definitions:  Normozoospermia: >20x10^6^/ml  Oligozoospermia: <20x10^6^/ml  Azoospermia: 0/ml  Semen analysis (14 survivors, 8 controls):  Normozoospermia: 2 survivors, 8 controls  Oligospermia: 3 survivors  Azoospermia: 9 survivors  Serum FSH Concentration (Male): Significantly elevated at basal and following GnRH stimulation (p<0.01)  Serum LH Concentration (Male): Significantly elevated at basal and following Gonadotrophin Releasing Hormone (GnRH) stimulation (p<0.05)  Testicular volume: Significantly reduced as measured by ultrasound (p<0.001) and Prader orchidometer (p<0.01) | Yes | Cumulative doses for various chemotherapeutic agents including cisplatin were calculated; however only data for alkylating agents were shown. Azoospermic survivors were treated with higher doses of radiation and alkylating agents compared to normospermic survivors. Basal FSH levels correlated with testicular volume in long-term survivors. Basal FSH levels correlated negatively with testicular volume measured by Prader orchidometer. |
| Hale GA, Marina NM, Jones-Wallace D, Greenwald CA, Jenkins JJ, Rao BN, et al (1999). Late effects of treatment for germ cell tumors during childhood and adolescence. *Journal of pediatric hematology/oncology* 21, 115-122 | | | | | |
| Single-centre cohort study | 73 (26 male, 47 female)  Age at diagnosis: Median 9.2 (1 day-18.3) years  Diagnosis: Extracranial germ cell tumour | Chemotherapy:  1962-1978: vincristine, actinomycin, cyclophosphamide (VAC) ± doxorubicin  1979-1988: VAC; cisplatin, vinblastine, bleomycin/etoposide (PVB); VAC and PVB  Radiotherapy: 25-30 Gy abdomen and pelvis or 20 Gy mediastinum and supraclavicular regions  Surgery: Surgical resection followed by observation for all stage I Germ cell tumours (GCTs)  Chemotherapy + surgery (n=27)  Radiotherapy + chemotherapy + surgery (n=21)  Radiotherapy + surgery (n=8)  Surgery alone (n=17) | Pubertal delay: No male patients reported pubertal delay  Testosterone replacement: No male patients required testosterone replacement  Impotence: One patient reported impotence following retroperitoneal lymph node dissection (RPLND)  Ejaculatory function: One patient reported ejaculatory dysfunction following Retroperitoneal lymph node dissection (RPLND) | No | Information about reproductive function not frequently evaluated among male patients. |
| Relander T, Cavallin-Stahl E, Garwicz S, Olsson AM, Willen M (2000). Gonadal and sexual function in men treated for childhood cancer. *Medical and pediatric oncology* 35: 52-63. | | | | | |
| Single-centre cohort study | 77 male childhood cancer patients  Age at diagnosis: 11 (10 months-17) years  Age at follow-up: Median 23.6 (18.6-38.5) years  Diagnoses: Leukaemia (n=9), Hodgkin disease (n=11), Non-Hodgkin Lymphoma (n=7), Pituitary (n=7), Pineal (n=3), other Central Nervous System (CNS) tumours (n=14), Sarcoma (n=5), Testis (n=3), Others (n=11) | Surgery only (n=16)  Radiotherapy only (n=6)  Surgery + radiotherapy (n=19)  Chemotherapy only (n=1)  Chemotherapy + surgery/radiotherapy (n=35) | Semen analysis definitions:  Normozoospermia: >20x10^6^/ml  Oligozoospermia: ≥5  and <20×10^6^/ml  Severe oligozoospermia: <5x10^6^/ml  Azoospermia: 0/ml  Patients without androgen substitution (n=66):  *Semen analysis* (n=59): Normozoospermia (n=34), Oligozoospermia: (n=6),  Severe oligozoospermia (n=5), Azoospermia (n=9)  Unable to provide sample (n=5)  *Sex hormones*:  1 patient with subnormal free testosterone, 14 pmol/liter (normal 31-163 pmol/liter) and borderline testosterone, 6.6 nmol/liter (normal 6-30 nmol/liter)  *Sexual function and progeny*: 20 patients report abnormalities in sexual function  Patients receiving androgen substitution (n=11):  *Testicular size*: 10 patients with testicular size <2ml, 1 patient 14 ml  *Semen analysis* (n=4): All azoospermic | No | 2 patients received cisplatin-containing regimens. 1 patient with Ewing’s sarcoma who received testicular irradiation, cyclophosphamide of doses up to 31800 mg/m^2^, actinomycin D, anthracyclines, methotrexate, vincristine, bleomycin and cisplatin 480 mg/m^2^ was observed to have testicular size of 13 ml. Another 2 patients who received similar regimens but without cisplatin were found to be azoospermic. 1 patient with testicular tumour received unilateral orchiectomy, retroperitoneal lymph node dissection, cisplatin 600 mg/m^2^, etoposide, vinblastine and bleomycin had small testicle (8 ml) and normozoospermia. |
| Longhi A, Macchiagodena M, Vitali G, Bacci G (2003). Fertility in male patients treated with neoadjuvant chemotherapy for osteosarcoma. *Journal of pediatric hematology/oncology* 25, 292-296. | | | | | |
| Single-centre cohort study | 96 male patients  Age at diagnosis: 17 (10-42) years  Age at follow-up: 27 (19-53) years  Diagnosis: Osteosarcoma | Chemotherapy: Methotrexate, cisplatin, doxorubicin, ifosfamide, etoposide | Semen analysis definitions:  Normozoospermia: >20x10^6^/ml, sperm motility 30-50%, morphology normal in 30%  Oligozoospermia: 1-20x10^6^/ml  Azoospermia: <1x10^6^/ml  Semen analysis (n=26): Normospermia (n=6), Oligospermia (n=1), Azoospermia (n=19)  Gonadotrophins and sex hormones (n=5):  5 patients had normal testosterone and raised LH and FSH levels  Pubertal development (Prepubertal patients): Median age at puberty 14 (12-16) years  Normal pubertal maturation | No | 11 patients were prepubertal at the time of chemotherapy; of these, only 1 patient underwent semen analysis and was azoospermic.  The incidence of azoospermia related to ifosfamide therapy versus no ifosfamide was statistically significant (p=0.005).  5 of 10 patients who did not receive ifosfamide were found to be azoospermic. These patients received cisplatin, methotrexate and doxorubicin at varying doses. |
| Ridola V, Fawaz O, Aubier F, Bergeron C, de Vathaire F, Pichon F, et al (2009). Testicular function of survivors of childhood cancer: a comparative study between ifosfamide- and cyclophosphamide-based regimens. *European journal of cancer* 45, 814-818. | | | | | |
| Multi-centre cohort study | 159 male childhood cancer survivors  Age at diagnosis:  Median 14 (9-19) years  Age at follow-up:  *Cyclophosphamide group*: Median 19.5 (17.5-28.6) years  *Ifosfamide group*: Median 22.5 (17.3-36.1) years  Diagnosis: Soft tissue sarcoma (n=79), Osteosarcoma (n=39), Ewing’s sarcoma (n=10), Non-Hodgkin’s lymphoma (n=28), Other (n=3) | Chemotherapy:  Cyclophosphamide (n=59), Ifosfamide (n=100), cisplatin (n=42)  Eligible patients could have received platinum therapy but not other alkylating agents or pelvic, gonadal or cranial irradiation | FSH:  *Cyclophosphamide group*: 28 (47.4%) had abnormal levels  *Ifosfamide group*: 6 (6%) had abnormal levels  LH:  *Ifosfamide group*: 14 (14%) had elevated levels  Testosterone:  *Ifosfamide group*: 2 had low testosterone  Fatherhood:  *Cyclophosphamide group*: 8/59 fathered at least one child  *Ifosfamide group*: 6/100 fathered at least one child | No | Adding cisplatin/carboplatin to alkylating agents had no effect on testicular function |
| Romerius P, Stahl O, Moell C, Relander T, Cavallin-Stahl E, Wiebe T, et al. High risk of azoospermia in men treated for childhood cancer (2011). *International journal of andrology* 34, 69-76. | | | | | |
| Cohort study | 129 male childhood cancer survivors  Age at diagnosis: Median 10 (0.1-17) years  Age at follow-up: Median 29 (20-46) years  Diagnoses: Leukaemias (n=21), Brain tumours (n=27), Hodgkin’s disease (n=19), Non-Hodgkin’s lymphoma (n=9), Testicular cancer (n=9), Wilms’ tumour (n=11), Others (n=33) | Brain surgery (n=16), Surgery (excluding brain surgery) only (n=16), chemotherapy only (n=35), radiotherapy to testes (n=1), non-testicular radiotherapy (n=13), chemotherapy + radiotherapy (n=48) | Semen analysis definitions: Azoospermia: 0 sperms  Semen analysis (n=129):  23 (18% azoospermic)  Chemotherapy + radiotherapy: 16 (33%) azoospermic  Chemotherapy only: 5 (14%) azoospermic  Surgery (excluding brain surgery): 1 (6.3%) azoospermic  Radiotherapy to testis: 1 (100%) azoospermic  Prepubertal (≤10 years at diagnosis (n=67): 10 (15%) azoospermic  Chemotherapy + radiotherapy: 7 (27%) azoospermic  Chemotherapy only: 2 (10%) azoospermic  Radiotherapy to testes 1 (100%) azoospermic  Sterilizing doses of cisplatin/alkylating agents:  + radiotherapy (n=14): 9 (64%) azoospermic  - radiotherapy (n=5): 4 (80%) azoospermic  Non-sterilizing doses of cisplatin/alkylating agents:  + radiotherapy (n=21): 7 (33%) azoospermic  - radiotherapy (n=19): 1 (5.3%) azoospermic | No | Sterilizing thresholds for chemotherapeutic agents were determined based on previous reports. For cisplatin, cumulative doses ≥500 mg/m^2^ were considered sterilizing |
| Tromp K, Claessens JJM, Knijnenburg SL, van der Pal HJH, van Leeuwen FE, Caron HN, et al (2011). Reproductive status in adult male long-term survivors of childhood cancer. *Human reproduction* 26, 1775-1783. | | | | | |
| Single-centre cohort study | 565 male childhood cancer survivors  Age at diagnosis: Median 7.8 (0-17.8) years  Age at follow-up: Median 21 (18-46) years  Diagnoses: Leukaemia (n=125), lymphoma (n=154), kidney tumour (n=64), brain/CNS tumour (n=47), bone tumour (n=53), soft tissue sarcoma (n=70), neuroblastoma (n=19), endocrine tumours (n=5), testicular tumour (n=9), other (n=19) | Radiotherapy only (n=4), chemotherapy only (n=127), surgery only (n=29), radiotherapy + chemotherapy (n=95), radiotherapy + surgery (n=30), chemotherapy + surgery (n=172), radiotherapy + chemotherapy + surgery (n=108)  Chemotherapy:  Alkylating agents only (n=34), anti-metabolites only (n=1), vinca alkaloids (n=67), alkylating agents + anti-metabolites (n=8), alkylating agents + vinca-alkaloids (n=170), antimetabolites + vinca-alkaloids (n=86), alkylating agents + anti-metabolites + vinca-alkaloids (n=124), others (n=9) | Outcome definitions:  FSH reference value: ≤10.0 U/l  LH reference value: ≤15.0 U/l  Testosterone reference value: ≥11.0 nmol/l  FSH (n=488):  ≤10.0 U/l: 327 (67.0%)  >10.0 U/l: 161 (33.0%)  LH (n=489):  ≤15.0 U/l: 475 (97.1%)  >15.0 U/l: 14 (2.9%)  Testosterone (n=460):  <11.0 nmol/l: 57 (12.4%)  ≥11.0 nmol/l: 403 (87.6%)  Pregnancy outcome:  73 men reported partner pregnancies.  56 were able to conceive naturally, 3 by *in vitro* fertilization, 10 by artificial inseminaton and 4 unknown. | No | Univariate and multivariate regression analyses did not identify cisplatin/carboplatin as significant risk factors for elevated FSH (Univariate p=0.251, Multivariate p=0.085).  Univariate linear regression only identified total body irradiation as a significant risk factor for decreased testosterone. |
| Odagiri K, Omura M, Hata M, Aida N, Niwa T, Ogino I, et al (2012). Treatment outcomes, growth height, and neuroendocrine functions in patients with intracranial germ cell tumors treated with chemoradiation therapy. *International journal of radiation oncology, biology and physics* 84: 632-638. | | | | | |
| Single-centre retrospective study | 22 patients (14 male, 8 female)  Age at diagnosis: Median 11.5 (6-19) years  Diagnosis: Intracranial germ cell tumour | Chemotherapy:  Carboplatin and etoposide (n=18)  Ifosfamide, carboplatin and etoposide (n=2)  Vincristine, etoposide, cyclophosphamide and cisplatin (n=2)  Radiotherapy:  Whole body irradiation (n=17)  Craniospinal irradiation (n=5) | Neuroendocrine dysfunction:  Gonadotropin deficiency from initial diagnosis: 2  Gonadotropin deficiency at last assessment but not at initial diagnosis: 4 | No | All patients with gonadotropin deficiency had tumours adjacent to the hypothalamic-pituitary axis. |
| Reinmuth S, Hohmann C, Rendtorff R, Balcerek M, Holzhausen S, Muller A, et al (2013). Impact of chemotherapy and radiotherapy in childhood on fertility in adulthood: the FeCt-survey of childhood cancer survivors in Germany. *Journal of cancer research and clinical oncology* 139, 2071-2078. | | | | | |
| Multi-centre cohort study | 618 childhood cancer survivors (234 male, 384 female)  Age at diagnosis: Median 10 (0-15) years  Age at follow-up: Median 30 (19-43) years  Diagnoses: Intracranial germ-cell tumour, brain tumour, liver tumour, nephroblastoma, neuroblastoma, extracranial germ-cell tumour, soft tissue tumour, osteosarcoma, Ewing sarcoma, non-Hodgkin lymphoma, acute myeloid leukaemia, acute, lymphoblastic leukaemia | Only data for chemotherapeutic medications suspected of causing infertility shown  Chemotherapy:  Cyclophosphamide (Male):  1-4999 mg/m^2^ (n=171)  ≥5000 mg/m^2^ (n=34)  Carboplatin/Cisplatin (Male):  1-1999/1-499 mg/m^2^ (n=25)  ≥2000/≥500 mg/m^2^ (n=5)  Etoposide:  1-4999 mg/m^2^ (n=27)  ≥5000 mg/m^2^ (n=4)  Radiotherapy:  No radiation or non-pelvic radiation (n=226)  Pelvic radiation 14-60 Gy (n=6) | Outcome definitions (Male):  Fertile/Probably fertile: Fathered a child, caused pregnancy or fertility test normal  Probably infertile: Tried to father a child unsuccessfully for at least 24 months (if partner not infertile) or abnormal fertility test result  Fertility status (Male):  Fertile/Probably fertile: 177 (75.6%)  Probably infertile: 57 (24.4%) | Yes | Postpubertal/peripubertal male patients were more likely to develop infertility compared to prepubertal patients. This difference was significant under univariable analysis (p=0.011) but not after multivariable analysis.  Carboplatin/cisplatin exposure was associated with a higher risk of developing infertility among male patients compared to a reference group of patients not receiving platinum agents; however this increased risk was not significant |
| Green DM, Liu W, Kutteh WH, Ke RW, Shelton KC, Sklar CA, et al (2014). Cumulative alkylating agent exposure and semen parameters in adult survivors of childhood cancer: a report from the St Jude Lifetime Cohort Study. *The Lancet Oncology* 15, 1215-1223. | | | | | |
| Multi-centre cohort study | 214 male patients  Age at diagnosis: Median 7 (0.01-20.3) years  Age at follow-up: Median 29 (18.4-56.1) years  Diagnoses: ALL (n=70), Acute Myeloid Leukaemia (n=5), Ewing sarcoma (n=5), Hodgkin’s lymphoma (n=2), germ cell tumour (n=2), melanoma (n=2), histiocytosis (n=2), liver malignancies (n=2), non-Hodgkin lymphoma (n=53), neuroblastoma (n=26), osteosarcoma (n=32), retinoblastoma (n=7), rhabdomyosarcoma (n=4), soft tissue sarcoma (n=3) | Chemotherapy:  Cyclophosphamide (n=195), Ifosfamide (n=26), Procarbazine (n=2), Chlormethine (n=1), Busulfan (n=3), Cisplatin (n=48), Carboplatin (n=22), Dacarbazine (n=3) | Semen analysis definitions:  Normospermic: >15x10^6^/ml  Oligospermic: <15x10^6^/ml  Azoospermic: 0/ml  Semen analysis:  Normospermia: 102 (48%)  Oligospermia: 59 (28%)  Azoospermia: 53 (25%)  Cyclophosphamide equivalent dose correlated negatively with sperm concentration (p<0.0001) | No | Treatment with cisplatin did not increase prevalence of azoospermia in subgroup treated for neuroblastoma/osteosarcoma (n=20), all of whom also received alkylator treatment (Statistical analysis not shown). |
| Green DM, Nolan VG, Goodman PJ, Whitton JA, Srivastava D, Leisenring WM, et al (2014). The cyclophosphamide equivalent dose as an approach for quantifying alkylating agent exposure: a report from the Childhood Cancer Survivor Study. *Pediatric blood and cancer* 61, 53-67. | | | | | |
| Multi-centre cohort study | 4579 male childhood cancer survivors  Age at diagnosis: 5-20 years  Diagnoses: Leukemia (n=1592), CNS tumour (n=662), Hodgkin disease (n=546), non-Hodgkin’s lymphoma (n=518), Wilms tumour (n=341), Neuroblastoma (n=164), Soft tissue sarcoma (n=348), Osteosarcoma (n=408) | Chemotherapy:  Agents included in cyclophosphamide equivalent dose (CED) calculation:  Cyclophosphamide, ifosfamide, procarbazine, chlorambucil, BCNU, CCNU, melphalan, Thio-tepa, nitrogen mustard, busulfan  Agents not included in CED calculation:  Actinomycin D (n=868), cisplatin (n=202), cytosine arabinoside (n=1138), daunorubicin (n=631), doxorubicin (n=1371), vinblastine (n=119), vincristine (n=3228), VM-26 (n=198), VP-16 (n=230) | Fatherhood:  Effect of cisplatin on fatherhood was evaluated individually (Statistical data not shown) | No | Of the patients who received cisplatin, 180 had no partners pregnant, while 22 had partners pregnant. |
| Wasilewski-Masker K, Seidel KD, Leisenring W, Mertens AC, Shnorhavorian M, Ritenour CW, et al (2014). Male infertility in long-term survivors of pediatric cancer: a report from the childhood cancer survivor study. *Journal of cancer survivorship: research and practice* 8, 437-447. | | | | | |
| Multi-centre cohort study | 1622 male childhood cancer survivors  Age at diagnosis: Mean 9 years  Age at study: Mean 37.2  Diagnoses: Acute Lymphoblastic Leukaemia (n=500), Acute Myeloid Leukaemia/Other leukemias (n=35), CNS tumours (n=138), Hodgkin lymphoma (n=259), Non-Hodgkin lymphoma (n=179), Kidney tumours (n=132), Neuroblastoma (n=81), soft tissue sarcoma (n=145), Ewing sarcoma (n=48), osteosarcoma (n=100), Other bone tumours (n=5) | Testicular irradiation:  <4Gy: 875 (58.5%)  ≥4 Gy: 114 (7.6%)  Total body irradiation:  24 (1.6%)  Exposure to chemotherapeutic agents was summed and an alkylating agent dose was calculated | Outcome definitions:  Infertility defined as positive response to question ‘Has a female partner ever had difficulty (it took more than a year) becoming pregnant by you?’  Infertility:  46% of survivors, 17.5% of sibling control group (p<0.001)  After multivariable analysis:  Alkylating Agent Dose (AAD) ≥3, surgical excision of organ of genital tract, testicular radiation dose ≥4 Gy were statistically significant independent risk factors for infertility | Yes | Platinum-containing agents were evaluated but not included in multivariable analysis due to lack of independent association with infertility |
| Brignardello E, Felicetti F, Castiglione A, Nervo A, Biasin E, Ciccone G, et al (2016). Gonadal status in long-term male survivors of childhood cancer. *Journal of cancer research and clinical oncology* 142, 1127-1132. | | | | | |
| Single-centre cohort study | 199 male childhood cancer survivors  Age at diagnosis:  0-10 (n=102)  ≥10 (n=97)  Diagnoses:  Acute Lymphoblastic Leukaemia (n=72), Hodgkin’s lymphoma (n=40), Non-Hodgkins Lymphoma (n=21), Acute Myeloid Leukaemia (n=12), brain tumours (n=28), sarcomas (n=15), others (n=11), | Radiotherapy (n=125):  Total body irradiation (n= 33)  Cranial (n=38)  Chemotherapy (n=187):  Alkylating agents (n=147)  Alkylating agents + platinum based (n=23)  Other combinations (n=17)  HSCT (n=48) | Gonadal dysfunction (n=92)  *Diagnosis of spermatogenesis damage*:  68 (34.17%)  *Primary hypogonadism:*  16 (8.04%)  *Central hypogonadism:*  13 (6.53%)  All patients treated with total body irradiation had gonadal dysfunction  Exposure to radiotherapy is associated with increased risk of gonadal dysfunction (adjusted OR=8.72)  Exposure to alkylators + platinum-based agents is associated with increased risk of gonadal dysfunction compared to only receiving alkylators (adjusted OR=9.22) | No |  |
| Chemaitilly W, Armstrong GT, Gajjar A, Hudson MM (2016). Hypothalamic-Pituitary Axis Dysfunction in Survivors of Childhood CNS Tumors: Importance of Systematic Follow-Up and Early Endocrine Consultation. *Journal of clinical oncology* 34, 4315-4319. | | | | | |
| Case study | 1 male patient  Age at diagnosis: 2.8 years  Diagnosis: Glioma | Chemotherapy:  Carboplatin, vincristine, temozolomide, procarbazine, lomustine, thioguanine | Tanner staging  Tanner 4 for pubic hair and penile size, Tanner 2 for testes (4.5 ml)  Plasma testosterone: Consistent with Tanner 4 (8.9 nmol/L) | No | The patient presented with hydrocephalus initially and had a residual suprasellar lesion. Contrast in testicular size and penile size/pubic hair staging may reflect effect of gonadotoxic agents |
| Chow EJ, Stratton KL, Leisenring WM, Oeffinger KC, Sklar CA, Donaldson SS, et al (2016). Pregnancy after chemotherapy in male and female survivors of childhood cancer treated between 1970 and 1999: a report from the Childhood Cancer Survivor Study cohort. *The Lancet Oncology* 17, 567-576. | | | | | |
| Multi-centre cohort study | 10938 childhood cancer survivors (5640 male, 5298 female)  Age at diagnosis (Male):  <5 (n=2085),  5-9 (n=1254),  10-14 (n=1287),  15-20 (n=1014)  Diagnoses (Male): Acute Lymphoblastic Leukaemia (n=1123), Other leukemias (n=300), Central Nervous System tumour (n=799), Hodgkin’s lymphoma (n=634), Kidney tumour (n=385), neuroblastoma (n=493), soft-tissue sarcoma (n=352), bone tumour (n=754) | Chemotherapy (Male): Busulfan (n=62), Carmustine (n=144), Carboplatin (n=158), Cisplatin (n=455), Chlorambucil (n=5), Chlormethine (n=244), Cyclophosphamide (n=2549), Dacarbazine (n=225), Ifosfamide (n=320), Lomustine (n=69), Melphalan (n=40), Procarbazine (n=432), Temozolomide (n=1), Thiotepa (n=21), Unknown (n=64)  Radiotherapy (Male): Neck (n=135), Chest (n=644), Arms or legs (n=148)  Sterilising procedure (Male) (n=144) | Siring pregnancy  Treatment of male patients with upper tertile of cisplatin doses (≥488 mg/m^2^) significantly associated with reduced likelihood of siring pregnancy (p=0.0023)  Siring livebirths  Treatment of male patients with cisplatin doses ≥355 mg/m^2^ significantly associated with reduced likelihood of siring livebirths (p=0.038 for doses 355-487 mg/m^2^, p=0.0019 for doses ≥488 mg/m^2^) | Yes |  |
| Isaksson S, Bogefors K, Stahl O, Eberhard J, Giwercman YL, Leijonhufvud I, et al (2018). High risk of hypogonadism in young male cancer survivors. *Clinical endocrinology* 88, 432-441. | | | | | |
| Multi-centre cohort study | 427 male childhood cancer survivors (CCS)  165 testicular cancer survivors  Age at diagnosis (CCS only): Median 9.6 (IQR 5.4-15) years  Age at study (CCS only): Median 33.7 (Interquartile range: 30.2-40.1) years  Diagnoses (CCS only): Leukaemia (n=27), intracranial tumour (n=28), lymphoma (n=21), testicular cancer (n=6), Wilms tumour (n=8), bone tumour (n=6), other (n=29) | Surgery:  Brain surgery (n=15)  Other (n=20)  Chemotherapy (n=29)  Radiotherapy:  Radiotherapy (RT) to brain (n=12)  RT to brain + chemotherapy (n=16)  RT to testes (n=5)  RT other (n=5)  RT other + chemotherapy (n=23) | Outcome definitions:  Primary hypogonadism: Total testosterone (TT) <10 nmol/L, LH and FSH >10 IU/L with FSH > LH or TT <10 nmol/L, LH ≤10 IU/L and FSH >10 IU/L  Secondary hypogonadism: TT <10 nmol/L, LH and FSH ≤10 IU/L  Compensated hypogonadism:  TT ≥10 nmol/L, LH >10 IU/L or ongoing androgen replacement therapy  CCS had increased risk of developing hypogonadism compared to controls (p=0.025)  Risk of hypogonadism is increased in testicular cancer (p=0.027) or leukemia patients (p=0.008) | Yes | There were no analyses of effects of cisplatin in the CCS group; however, >4 cycles of cisplatin (>400 mg/m^2^) in testicular cancer survivors was significantly associated with increased risk of hypogonadism (p=0.015). The testicular cancer survivors comprised of adults (Mean age at diagnosis 30.8, standard deviation: 7.2 years) |
| Utriainen P, Suominen A, Makitie O, Jahnukainen K (2019). Gonadal Failure Is Common in Long-Term Survivors of Childhood High-Risk Neuroblastoma Treated With High-Dose Chemotherapy and Autologous Stem Cell Rescue. *Frontiers in endocrinology* 10, 555. | | | | | |
| Multi-centre cohort study | 20 patients (11 females, 9 males)  Age at diagnosis: Median 1.6 (0.2-3.6) years  Age at HSCT:  Median 2.3 (1.0-4.1) years  Age at follow-up:  Median 21.7 (15.9-30.1) years  Diagnosis:  Neuroblastoma | Radiotherapy:  Local irradiation (n=14)  Total body irradiation + chemotherapy (n=10)  Chemotherapy:  COD (Cyclophosphamide, vincristine and dacarbazine)  AAP (Cisplatin and doxorubicin)  Etoposide, carboplatin, thio-tepa/melphalan  Other chemotherapy combinations  Surgery:  Removal of original tumour (n=20) | Outcome definitions:  Gonadal failure:  Absent pubertal development, history of increased gonadotrophins after spontaneous puberty requiring hormone replacement therapy or small post-pubertal testis size  Gonadal failure:  6 male survivors had gonadal failure  Cumulative cisplatin dose did not correlate with testicular size.  All survivors who did not have gonadal failure did not undergo total body irradiation | Yes | 1 male patient had fathered a child (cisplatin and etoposide as induction chemotherapy, followed by high-dose melphalan). Patients may have received Total Body Irradiation (TBI) or Haematopoietic Stem Cell Transplant (HSCT).  Male survivors (n=6) had significantly higher FSH (26.3 v 3.6 IU; p<0.001) and lower cumulative testicular volumes (8.5 v 39 mls, p<0.001) compared with healthy controls. 3/5 patients in the non-TBI group had testicular volumes >15mls, whereas 4/4 patients receiving TBI had testicular volumes <10mls. No correlation between the cumulative cisplatin dose and the testicular volume. 8/9 patients entered puberty spontaneously (3 early), whilst one patient in the TBI group required pubertal induction. All 4 patients in the TBI group required testosterone replacement, whilst 0/5 in the no-TBI group required testosterone |
